# Supplementary material for: Homozygous EPRS1 missense variant causing hypomyelinating leukodystrophy-15 alters variant-distal mRNA m6A site accessibility
Source: Nat Commun. 2024 May 20;15:4284. doi: 10.1038/s41467-024-48549-x (PMC11106242; doi:10.1038/s41467-024-48549-x)
Supplement: Supplementary file 1 — Supplementary Information [file 41467_2024_48549_MOESM1_ESM.pdf]

Supplementary material for:

Homozygous EPRS1 missense variant causing hypomyelinating leukodystrophy-15 alters variant-distal mRNA m^6A site accessibility

Debjit Khan, Iyappan Ramachandiran, Kommireddy Vasu, Arnab China, Krishnendu Khan, Fabio Cumbo, Dalia Halawani, Fulvia Terenzi, Isaac Zin, Briana Long, Gregory Costain, Susan Blaser, Amanda Carnevale, Valentin Gogonea, Ranjan Dutta, Daniel Blankenberg, Grace Yoon and Paul L. Fox

Correspondence to: foxp@ccf.org, grace.yoon@utoronto.ca

This file includes

Supplementary Figures 1 to 17 (raw data supplied in figshare repository: https://doi.org/10.6084/m9.figshare.25607931)

Supplementary Tables 1 to 4

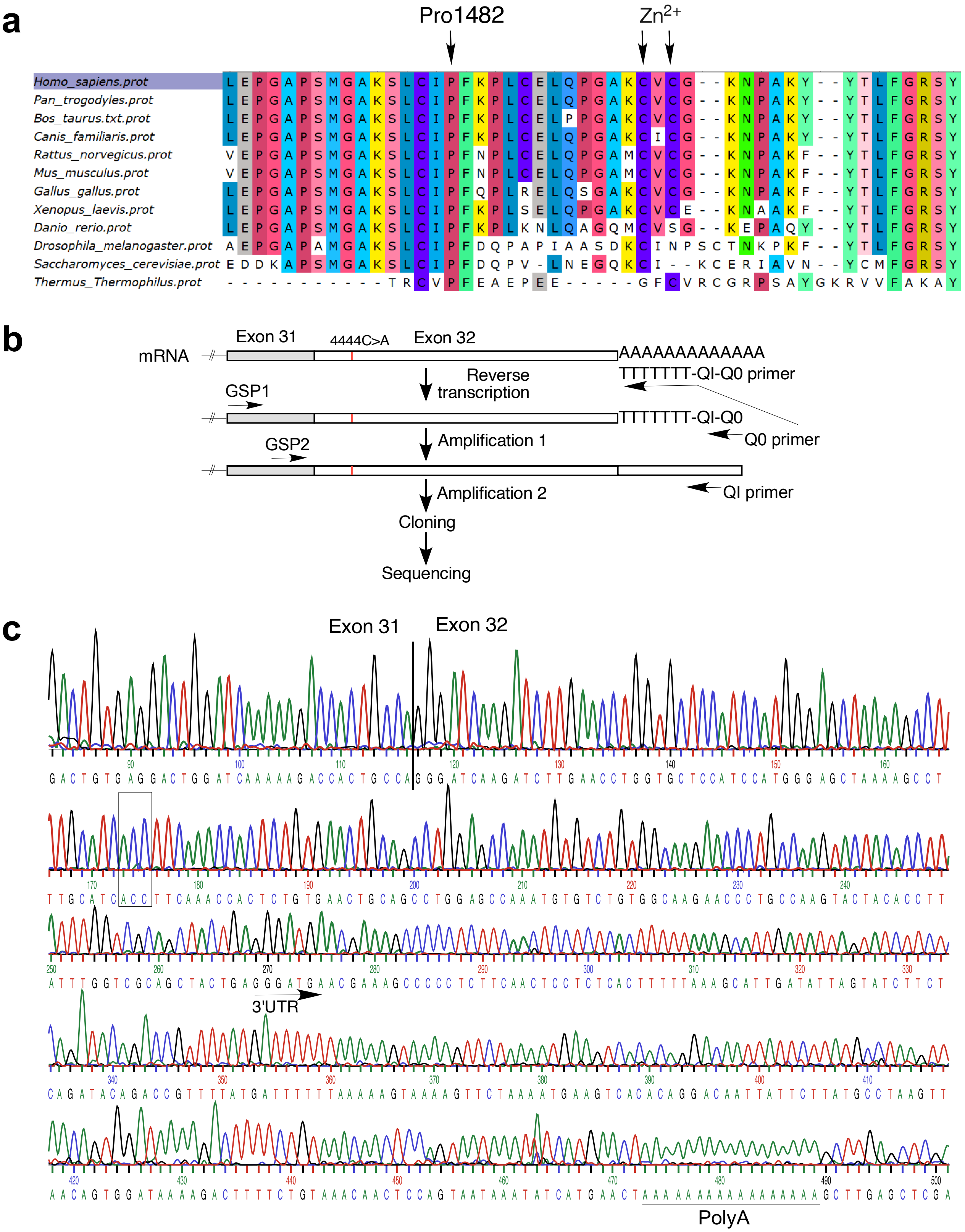

Supplementary Fig. 1 | Evolutionary conservation of EPRS1 Pro1482, and 3'RACE analysis of influence of the c.4444C>A mutation on splicing and polyadenylation. a Clustal Omega generated alignment of reference sequences of the EPRS1 protein in *Drosophila* and higher organisms or ProRS in yeast and lower organisms. Arrows indicates the position of Pro1482 and Zn^{2+} binding sites in human EPRS1. Alignment by Clustal Omega was visualized using Unipro Ugene. **b** Schematic representation of 3' RACE in patient LCLs. Exon 31-32 boundary is shown. Point mutation is indicated by red line. The 52-nt Q0-QI-TTTTTT primer is used to reverse transcribe cellular mRNAs and gene-specific primer 1 (GSP1) and primer 2 (GSP2) are used in sequential amplifications to generate sequence-specific product. **c** Chromatogram of exon 31-32 of *EPRS1* mRNA showing boundary, 3'UTR, polyA tail, and Pro1482Thr codon (boxed).

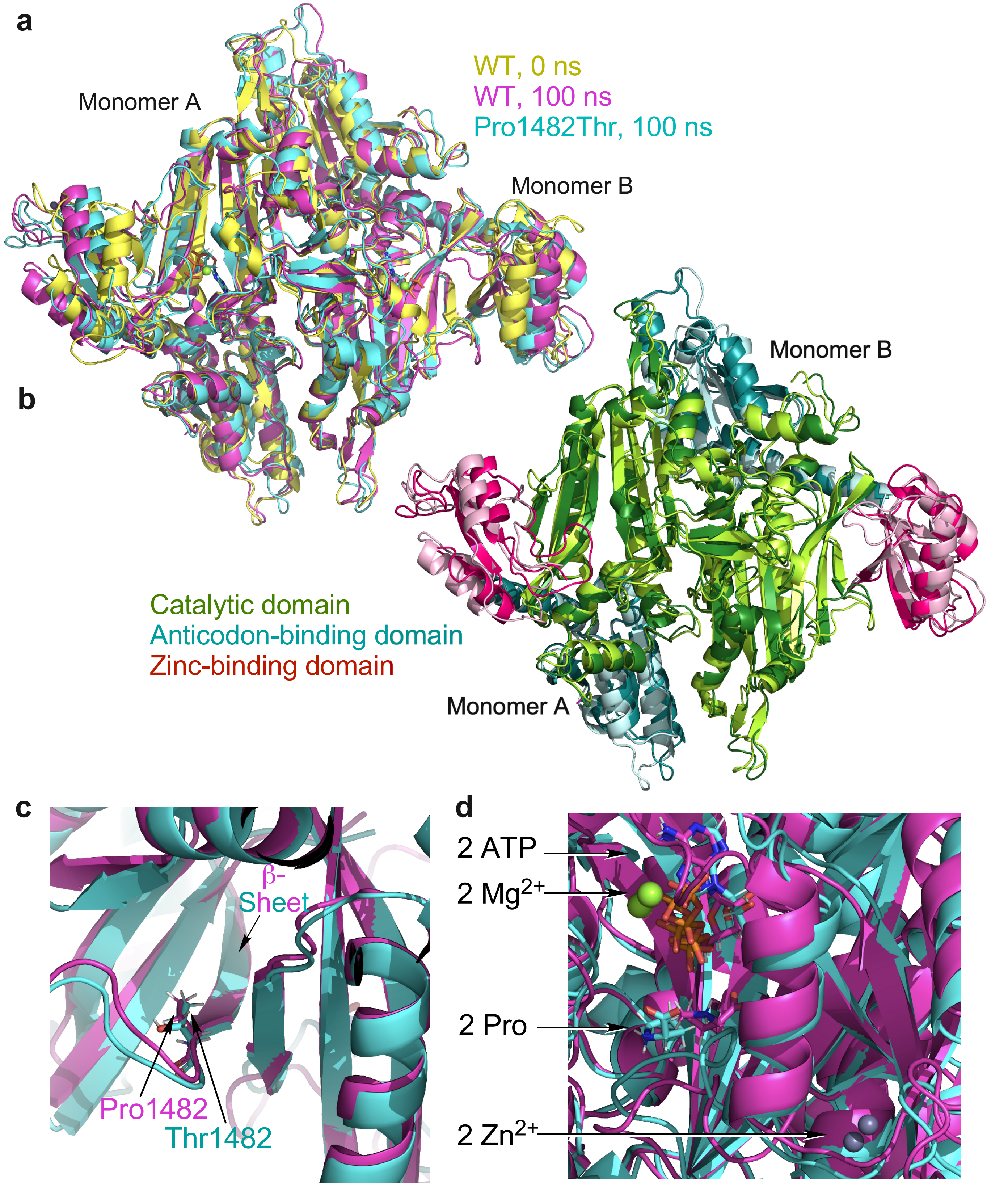

Supplementary Fig. 2 | Molecular dynamic simulation of WT and Pro1482Thr ProRS dimer. **a** Overlay of ProRS dimer crystal structure (yellow) with structures following 100 ns MDS of WT (pink) and variant (cyan). b Overlay of ProRS dimer crystal structure color-coded by domain: catalytic domain (green), anti-codon-binding domain (cyan) and Zinc-binding domain (pink). Lighter shades represent WT ProRS, darker shades represent the variant. **c** Enlargement of ProRS monomer B overlay, highlighting variant site amino acids at the 3'end of a b-sheet; colors as in (**a**). **d** Enlargement of ProRS monomer B overlay, highlighting locations of ATP, proline, and Mg2+ and Zn^{2+} ions; colors as in (**a**).

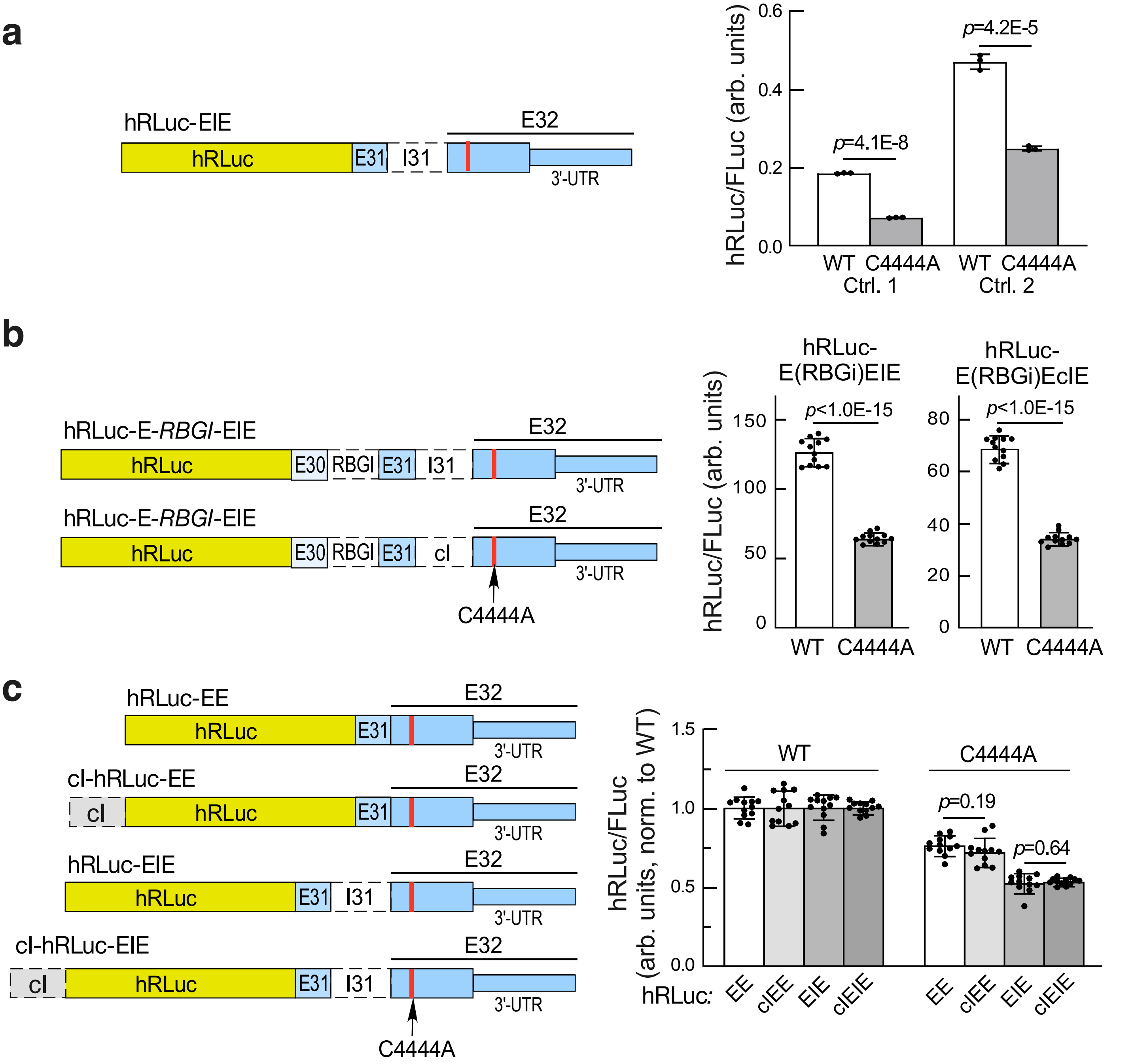

Supplementary Fig. 3 | Effect of intron placement on reporter expression. a Effect of C4444A mutation on chimeric hRLuc-EIE reporter (left) in control LCLs (right). Mean ± SD, n = 3 biological replicates; *p*-values are from unpaired two-tailed *t*-test. **b** Effect of replacement of I31 with alternate intron, cI, in triple-exon reporter (hRLuc-E-RBGI-EcIE, left). Normalized reporter activities of reporters with and without C4444A mutation in HEK293T cells (right). Mean ± SD, n = 12 biological replicates; *p*-values are from unpaired two-tailed *t*-test. **c** Non-synergy of a 5'intron (i.e., cI) in the 5'UTR of hRLuc-EE reporter (left, top two schematics) with the HLD-causing c.4444C>A variant in HEK293T cells (right). Evidence of synergy only when an intervening intron (e.g., I31) is present between exons 31 and 32. (left, bottom two schematics), highlights a specific role of this exon-exon junction. Mean ± SD, n = 12 biological replicates; *p*-values are from unpaired two-tailed *t*-test.

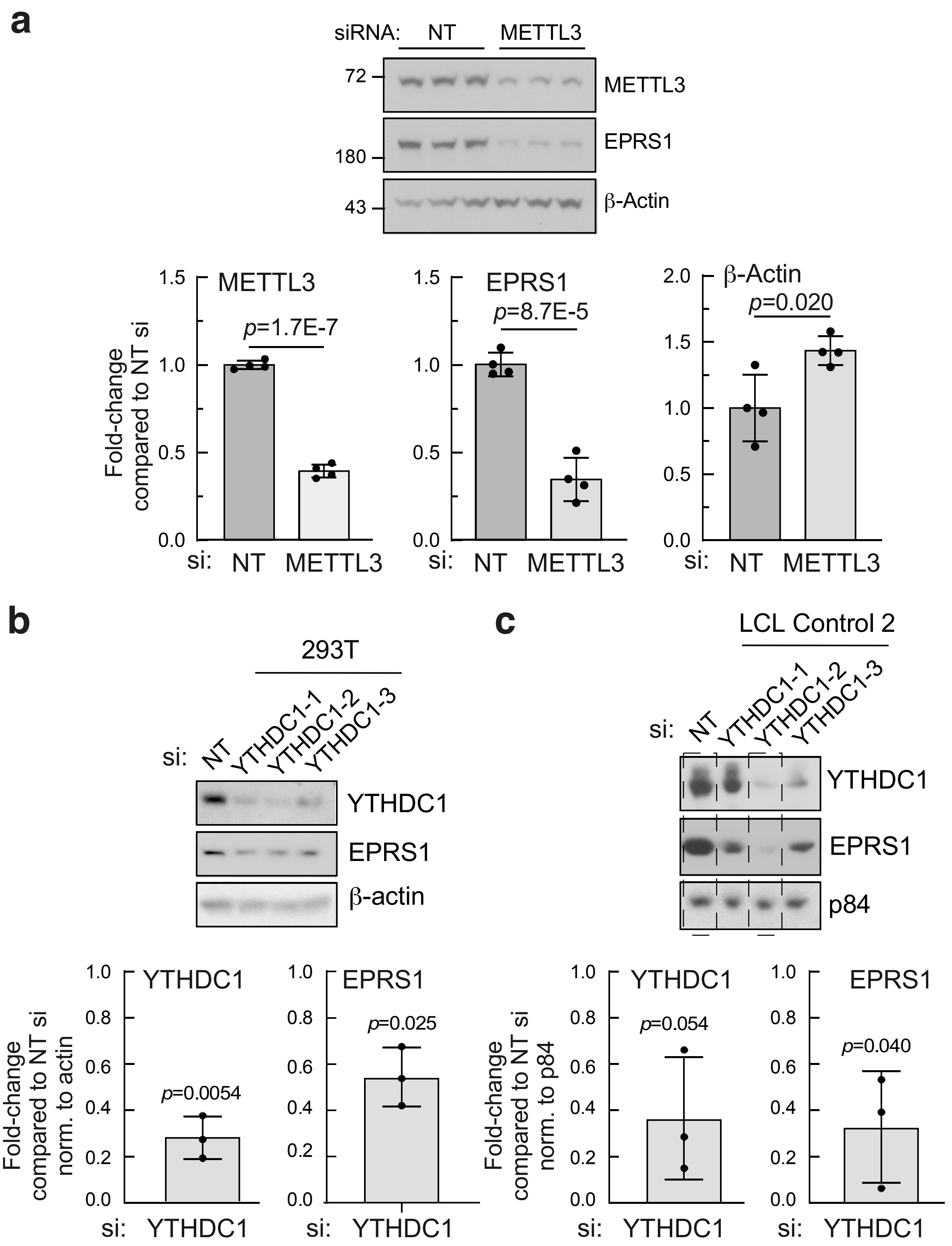

Supplementary Fig. 4 | Effect of knockdown of nuclear m^{6}A writer METTL3 and nuclear m^{6}A reader YTHDC1 on EPRS1 expression. a Effect of METTL3 knockdown on EPRS1 in 293T cells (top). Densitometric quantification across 4 biological replicates (top panel and Fig. 3d) in bottom three panels; *p*-values are from unpaired two-tailed *t*-test. **b,c** Effect of YTHDC1 knockdown on EPRS1 in 293T cells **(b)** and LCL control 2, with highlighted regions also shown in Fig. 3e (**c**). Densitometric quantification of three individual siRNA-mediated knockdowns in **b,c** (bottom panels). *p*-values from one-sample *t*-tests (two-tailed) to measure statistically significant deviation from fold-change = 1 (i.e., no change).

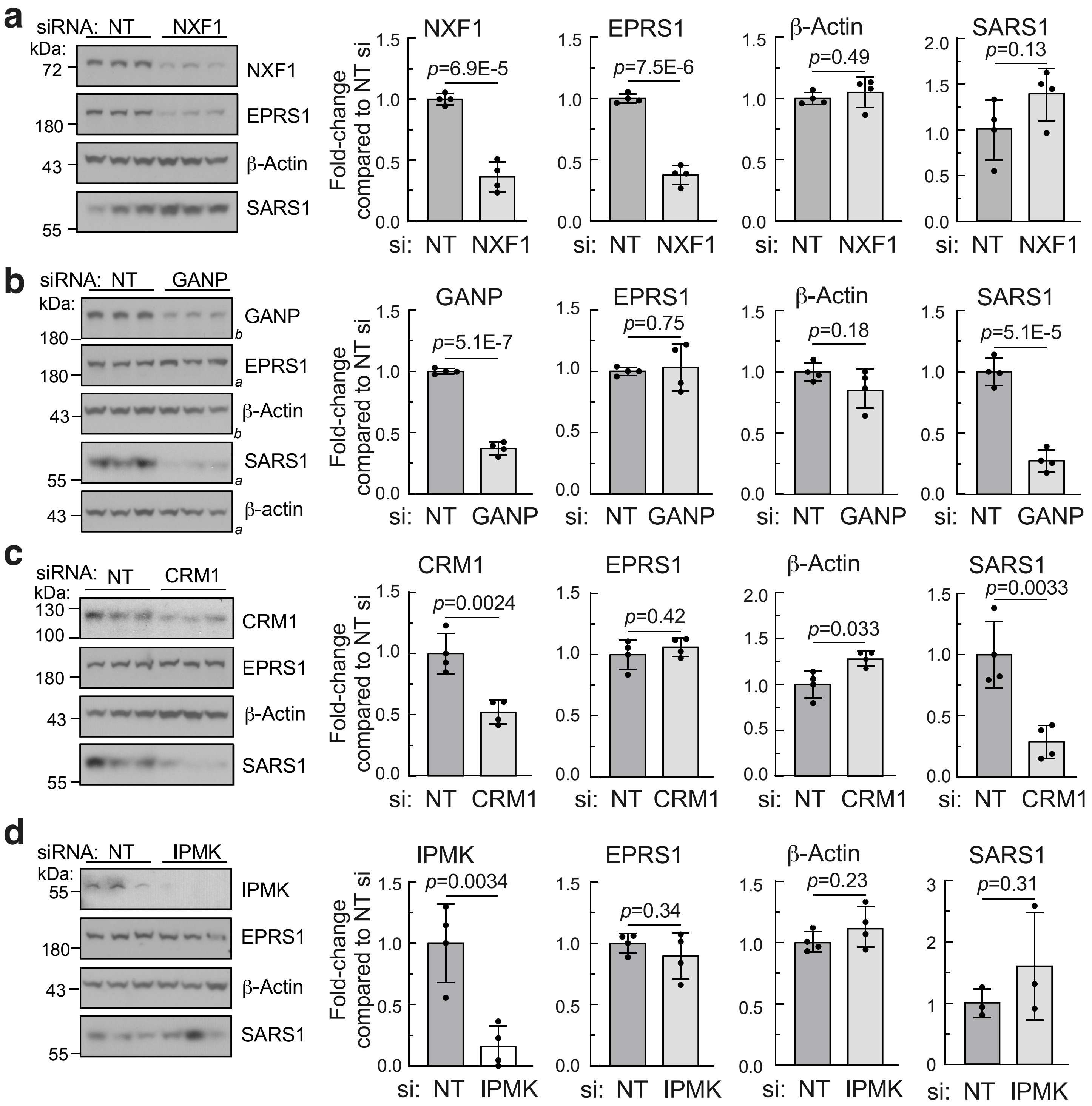

Supplementary Fig. 5 | Effect of knockdown of specific nuclear export pathways on EPRS1. a-d Effect of NXF1, GANP, CRM1 and IPMK knockdown on EPRS1 in 293T cells (left panels). Densitometric quantification across 4 biological replicates (left panels and Fig. 3f) in right 4 panels; *p*-values are from unpaired two-tailed *t*-test. In **b**, *a* and *b* refer to two different gels run, with loading control (β-actin) shown and quantitated from both gels.

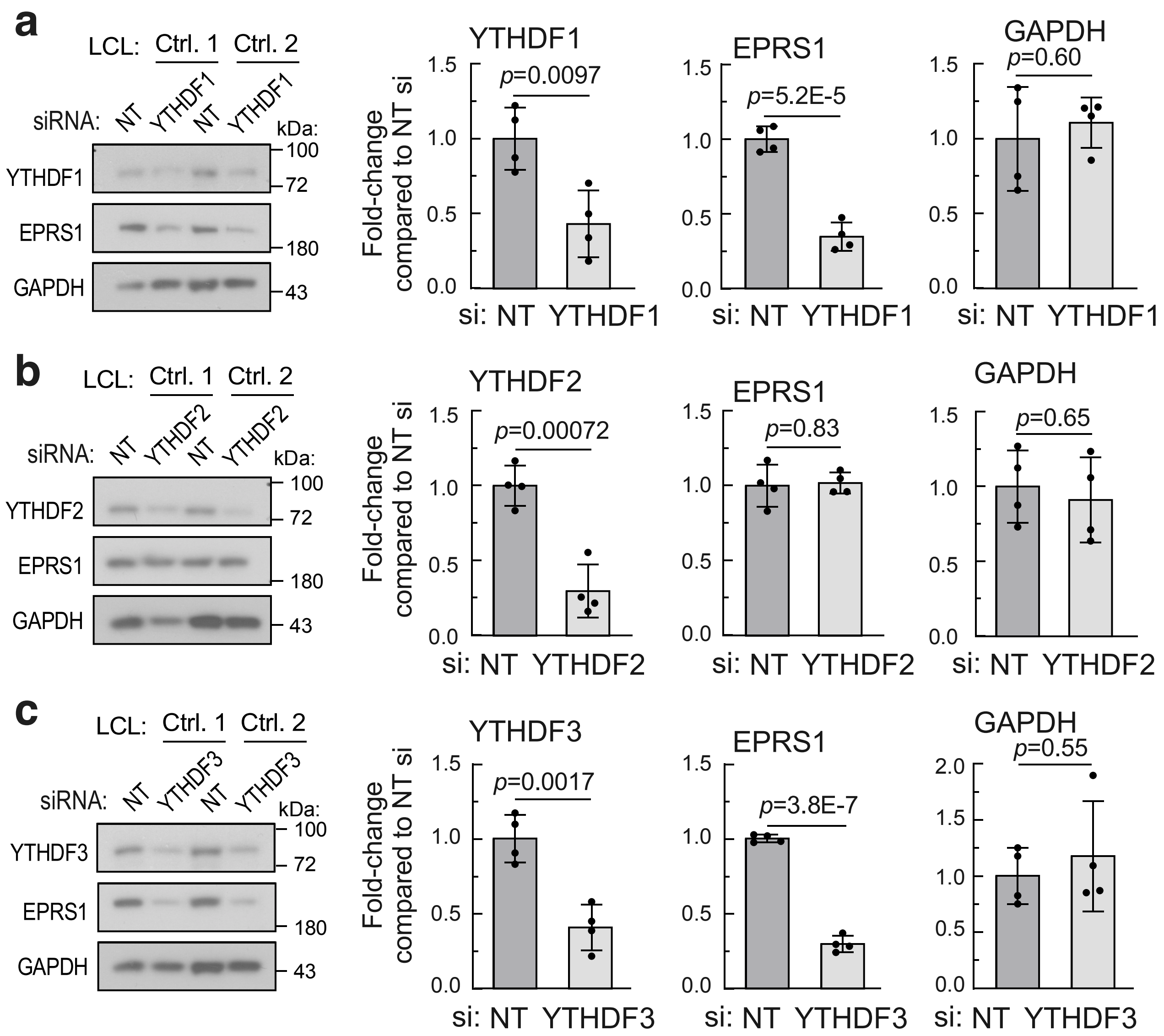

Supplementary Fig. 6 | Influence of YTHDF family of cytoplasmic m⁶A readers on EPRS1 expression. a-c LCLs were subjected to siRNA-mediated knockdown targeting YTHDF1 (**a**), YTHDF2 (**b**) and YTHDF3 (**c**). Densitometric quantification across 4 biological replicates (left panels and Fig. 4g), two each for two control LCLs. *p*-values are from unpaired two-tailed *t*-test.

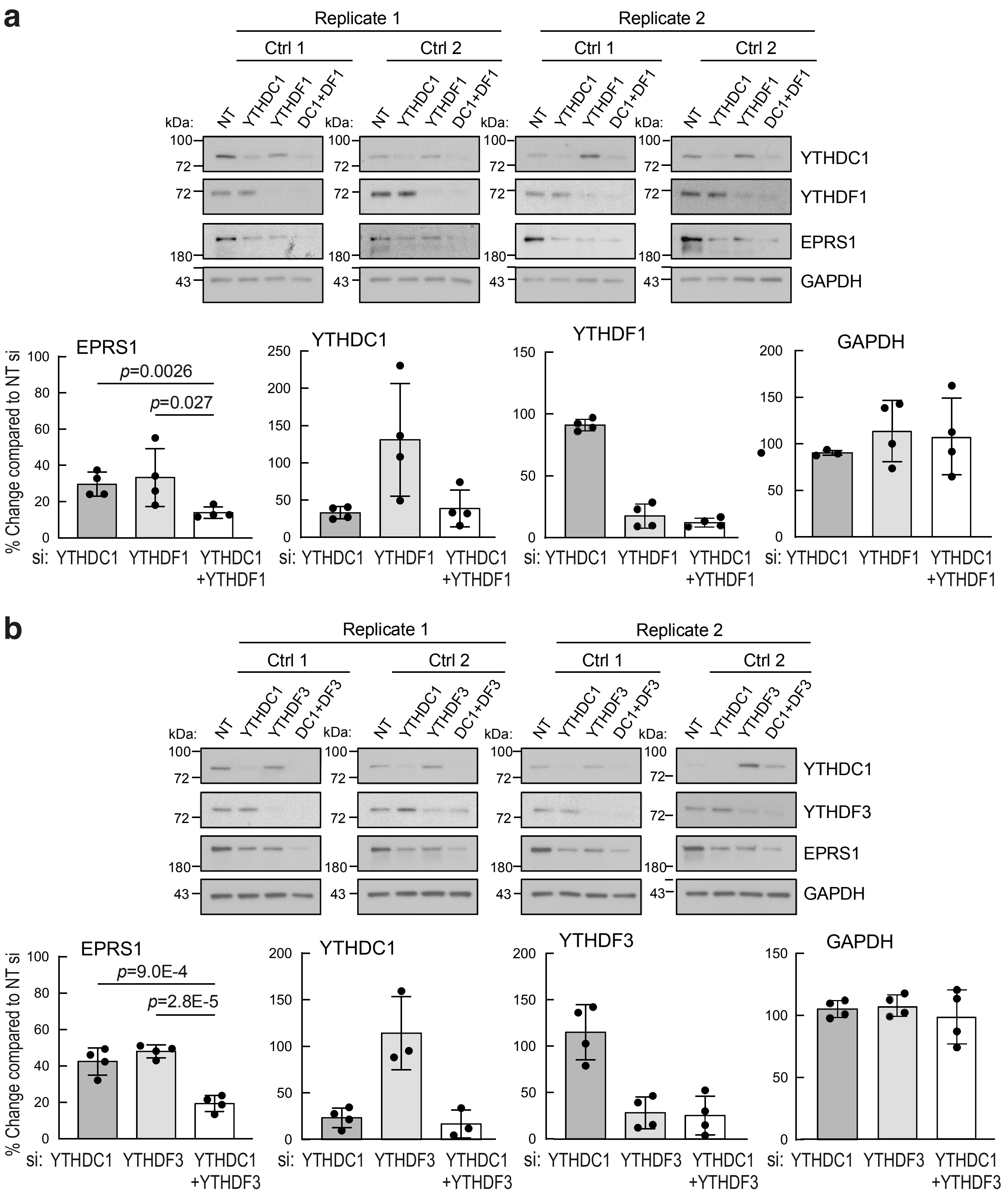

Supplementary Fig. 7 | Additive effect of combined knockdown of YTHDC1 and YTHDF1/YTHDF3 on EPRS1 expression. a-b LCLs were subjected to siRNA mediated knockdown of YTHDC1 and YTHDF1 (**a**) or YTHDC1 and YTHDF3 (**b**). Densitometric quantification across 4 biological replicates, two each for each control LCL, in bottom panels. *p*-values from unpaired *t*-test, one-tailed to measure statistically significant decrease in EPRS1 expression compared to single knockdowns. Outlier corrections used throughout (Grubbs' test at α=0.05) removed single outliers in siYTHDF3 and siYTHDC1+siYTHDF3 groups for YTHDC1 quantification in **b**.

```
hRLuc        ATGGCTTCCAAGGTGTACGACCCCGAGCAACGCAAACGCATGATCACTGGGCCTCAGTGG   60
DLRLT184S    ATGGCTTCCAAGGTGTACGACCCCGAGCAACGCAAACGCATGATCACTGGGCCTCAGTGG   60

hRLuc        TGGGCTCGCTGCAAGCAAATGAACGTGCTGGACTCCTTCATCAACTACTATGATTCCGAG   120
DLRLT184S    TGGGCTCGCTGCAAGCAAATGAACGTGCTCGATTCCTTCATCAACTACTATGATTCCGAG   120

hRLuc        AAGCACGCCGAGAACGCCGTGATTTTTCTGCATGGTAACGCTGCCTCCAGCTACCTGTGG   180
DLRLT184S    AAGCACGCCGAGAACGCCGTGATTTTTCTGCATGGTAACGCTGCCTCCAGCTACCTGTGG   180

hRLuc        AGGCACGTCGTGCCTCACATCGAGCCCGTGGCTAGATGCATCATCCCTGATCTGATCGGA   240
DLRLT184S    AGGCACGTCGTGCCTCACATCGAGCCCGTGGCTAGATGCATCATCCCTGATCTGATCGGA   240

hRLuc        ATGGGTAAGTCCGGCAAGAGCGGGAATGGCTCATATCGCCTCCTGGATCACTACAAGTAC   300
DLRLT184S    ATGGGTAAGTCCGGCAAGAGCGGGAATGGCTCATATCGCCTCCTGGATCACTACAAGTAC   300

hRLuc        CTCACCGCTTGGTTCGAGCTGCTGAACCTTCCAAAGAAAATCATCTTTGTGGGCCACGAC   360
DLRLT184S    CTCACCGCTTGGTTCGAGCTGCTCAATCTTCCAAAGAAAATCATCTTTGTGGGCCACGAC   360

hRLuc        TGGGGGGCTTGTCTGGCCTTTCACTACTCCTACGAGCACCAAGACAAGATCAAGGCCATC   420
DLRLT184S    TGGGGGGCTTGTCTGGCCTTTCACTACTCCTACGAGCACCAGATAAGATCAAGGCCATC    420

hRLuc        GTCCATGCTGAGAGTGTCGTGGACGTGATCGAGTCCTGGGACGAGTGGCCTGACATCGAG   480
DLRLT184S    GTCCATGCTGAGAGTGTCGTGGACGTGATCGAGTCCTGGGACGAGTGGCCCGATATCGAG   480

hRLuc        GAGGATATCGCCCTGATCAAGAGCGAAGAGGGCGAGAAAATGGTGCTTGAGAATAACTTC   540
DLRLT184S    GAGGATATCGCCCTGATCAAGAGCGAAGAGGGCGAGAAAATGGTGCTTGAGAATAATTTC   540
                   Thr
hRLuc        TTCGTCGAGACCATGCTCCCAAGCAAGATCATGCGGAAACTGGAGCCTGAGGAGTTCGCT   600
DLRLT184S    TTCGTCGAGTCCATGCTCCCAAGCAAGATCATGCGGAAGCTGGAGCCTGAGGAGTTCGCT   600
                   Ser
hRLuc        GCCTACCTGGAGCCATTCAAGGAGAAGGGCGAGGTTAGACGGCCTACCCTCTCCTGGCCT   660
DLRLT184S    GCCTACCTGGAGCCATTCAAGGAGAAGGGCGAGGTTAGACGGCCTACCCTCTCCTGGCCT   660

hRLuc        CGCGAGATCCCTCTCGTTAAGGGAGGCAAGCCCGACGTCGTCCAGATTGTCCGCAACTAC   720
DLRLT184S    CGCGAGATCCCTCTCGTTAAGGGAGGCAAGCCCGACGTCGTCCAGATTGTCCGCAACTAC   720

hRLuc        AACGCCTACCTTCGGGCCAGCGACGATCTGCCTAAGATGTTCATCGAGTCCGACCCTGGG   780
DLRLT184S    AACGCCTACCTTCGGGCCAGCGACGATCTGCCTAAGATGTTCATCGAGTCCGACCCTGGG   780

hRLuc        TTCTTTTCCAACGCTATTGTCGAGGGAGCTAAGAAGTTCCCTAACACCGAGTTCGTGAAG   840
DLRLT184S    TTCTTTTCCAACGCTATTGTCGAGGGAGCTAAGAAGTTCCCCAATACCGAGTTCGTGAAG   840

hRLuc        GTGAAGGGCCTCCACTTCAGCCAGGAGGACGCTCCAGATGAAATGGGTAAGTACATCAAG   900
DLRLT184S    GTGAAGGGCCTCCACTTCAGCCAGGAGGACGCTCCAGATGAAATGGGTAAGTACATCAAG   900

hRLuc        AGCTTCGTGGAGCGCGTGCTGAAGAACGAGCAG   933
DLRLT184S    AGCTTCGTGGAGCGCGTGCTGAAGAACGAGCAG   933
```

Supplementary Fig. 8 | Mutations introduced into hRLuc to inactivate DRACH sites. Synonymous mutations (boxed) were introduced except for a requisite Thr-to-Ser mutation (boxed with labels).

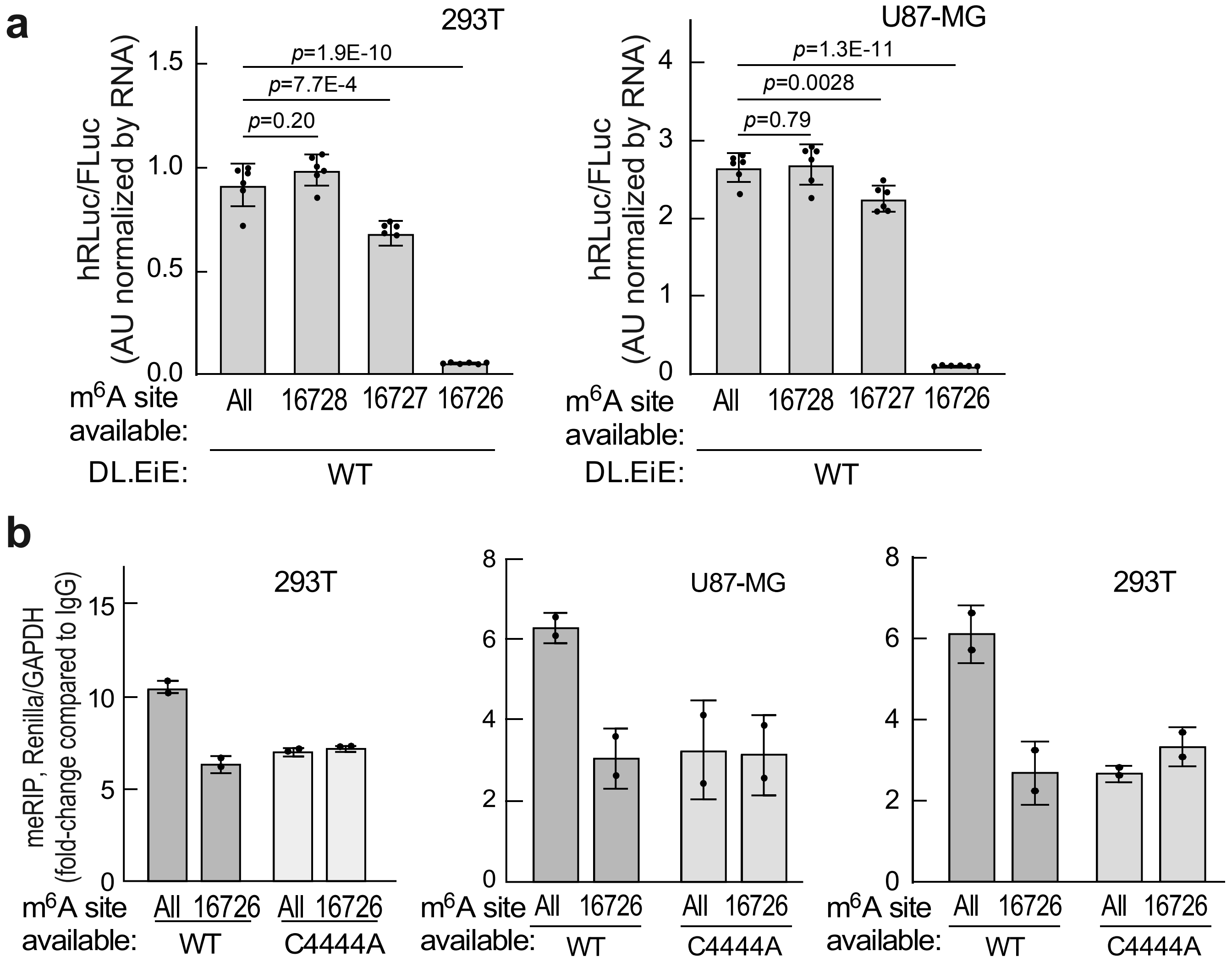

Supplementary Fig. 9 | Identification of functional m^{6}A sites in exons 31-32 of *EPRS1* mRNA. a Normalized expression of DRACH$^-$ hRLuc reporter in 293T (left) and U87-MG cells (right) following pairwise mutation of m^{6}A sites. Mean ± SD, n = 6 biological replicates in each cell line; *p*-values are from unpaired two-tailed *t*-test. Also refer to Fig. 4b. **b** Detection of m^{6}A modification of reporters by meRIP-RT-qPCR. Mean ± SD, n = 2 biological replicates each experiment. Also refer to Fig. 4c.

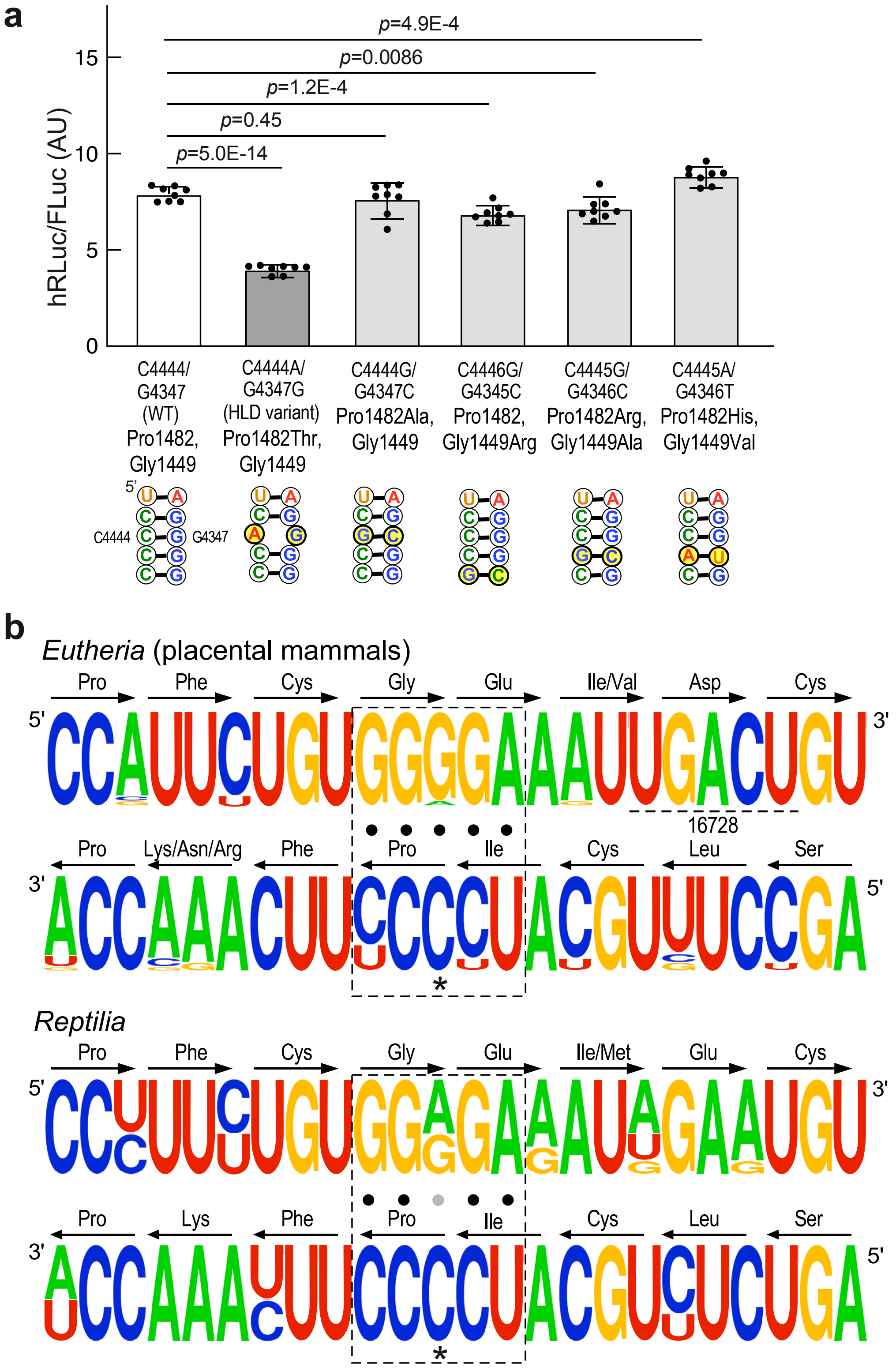

Supplementary Fig. 10 | Role of 5-bp stem in expression of c.4444C>A EPRS1 variant in patient LCLs. a Effect of mutations in the 5-bp stem surrounding C4444A site on hRLuc reporter expression in 293T cells. Mean ± SD, n = 8 biological replicates; *p*-values from unpaired, two-tailed *t*-test. **b** Sequence logo representations of bp conservation of the 5-bp stem surrounding the C4444A variant site (*) in human EPRS1 (dashed boxes). Displayed are placental mammals (top, 16728 m^6A site dashed underline) and reptilia (bottom); •, strong bp conservation, •, moderate bp conservation.

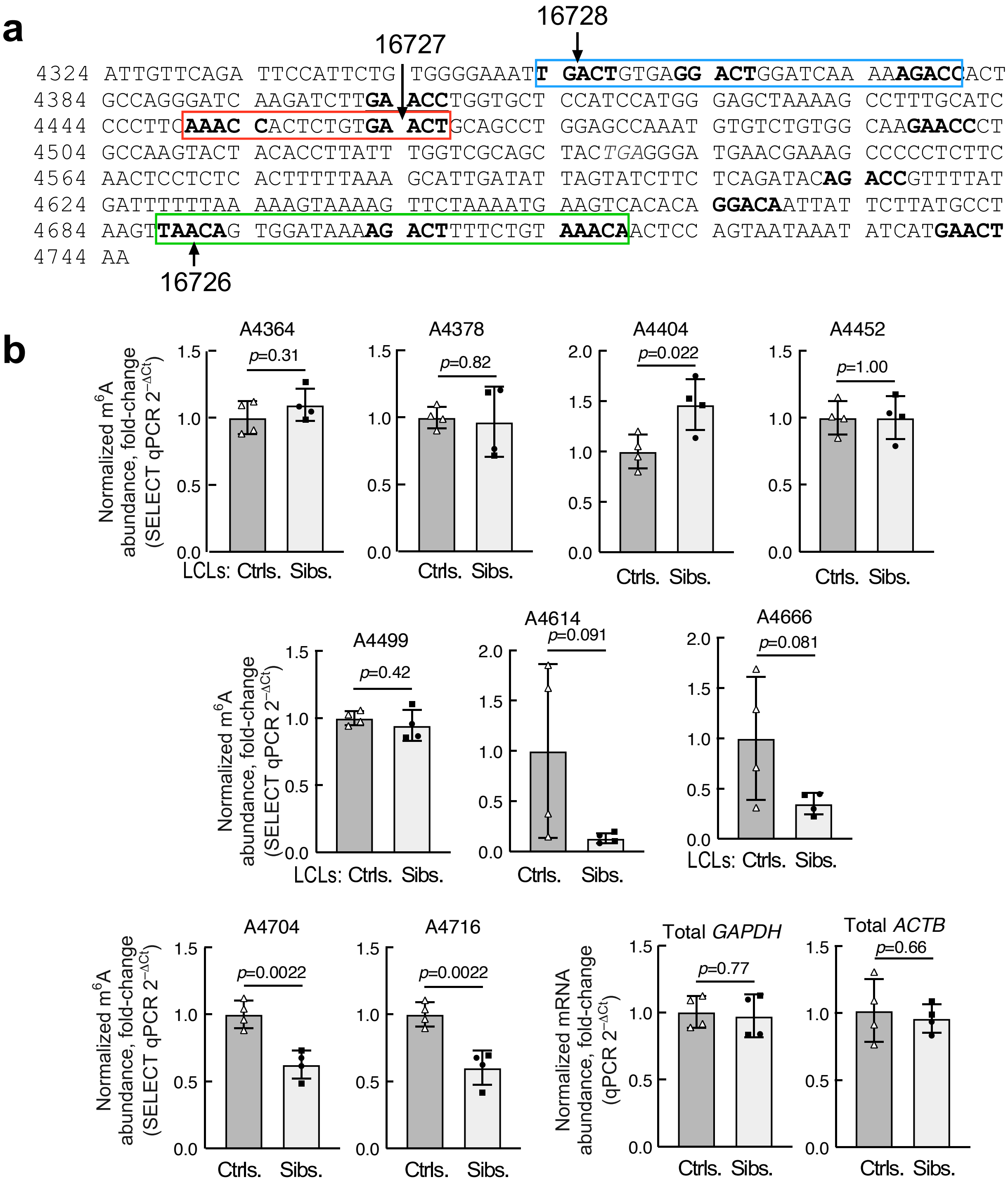

Supplementary Fig. 11 | SELECT-qPCR reveals differential m^{6}A methylation at specific DRACH sequences in the last two exons of *EPRS1* mRNA in patient LCLs. a Nucleotide sequence of the last two exons of *EPRS1* (start codon considered as 1, SNV site at 4444, stop codon italicized). All DRACH sequences (bold) and three potential polymethylated regions are highlighted (colored boxes). **b** SELECT-qPCR analysis of specific adenosine residues of underlined DRACH sequences shown in **a**. Two panels at bottom-right show RT-qPCR analysis of *GAPDH* and *ACTB* mRNAs in control and patient LCLs. Also refer to Fig. 5d. Mean ± SD, n = 4 pooled biological replicates for control and sibling LCLs; *p*-values are from unpaired two-tailed *t*-test.

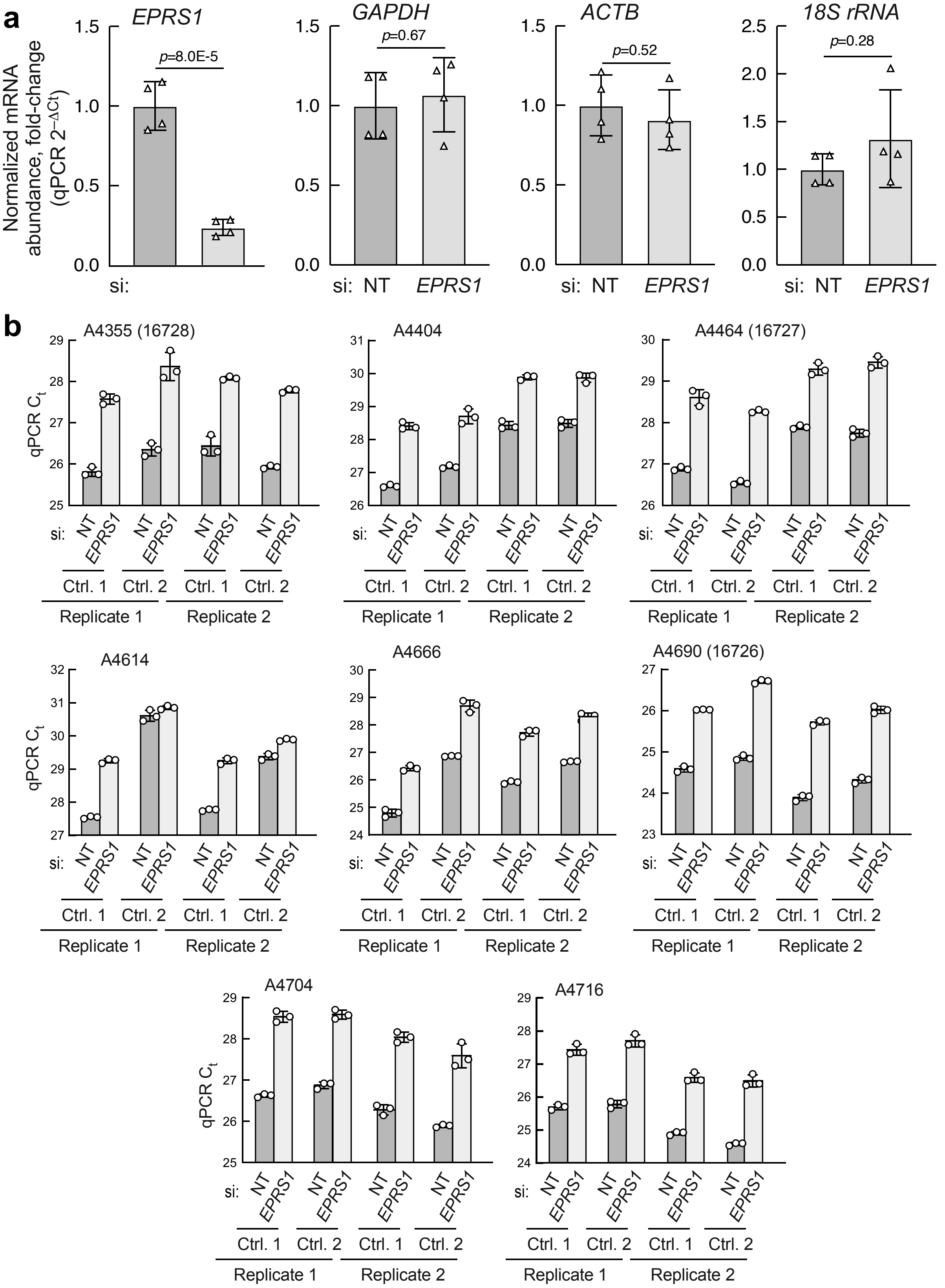

Supplementary Fig. 12 | EPRS1 specificity of SELECT-qPCR probe-pairs. a Control LCLs were subjected to *EPRS1* knockdown and confirmed by RT-qPCR from total RNA isolated from NT (non-targeting) siRNA nucleofected cells. **b** SELECT-qPCR was performed for relevant sites from RNA isolated from *EPRS1*-knockdown control LCLs and compared to NT-siRNA nucleofected control LCLs.

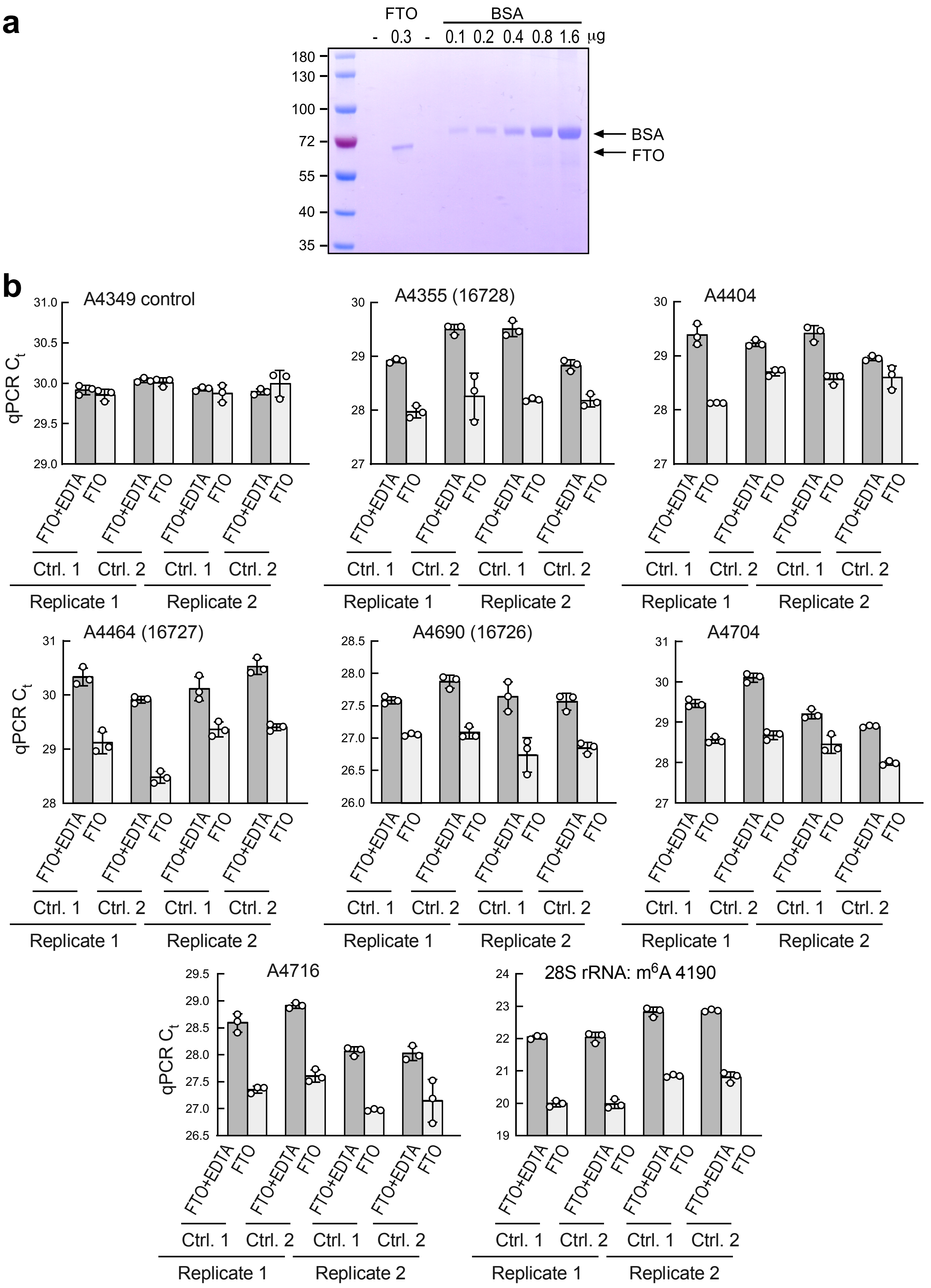

Supplementary Fig. 13 | m^{6}A specificity of SELECT-qPCR probe-pairs. a FTO demethylase was resolved on 4-12% SDS-PAGE to confirm integrity and concentration by Imperial stain. **b** SELECT-qPCR was performed for relevant sites from total RNA isolated from two control LCLs demethylated with FTO and compared to SELECT reactions done with EDTA pre-quenching.

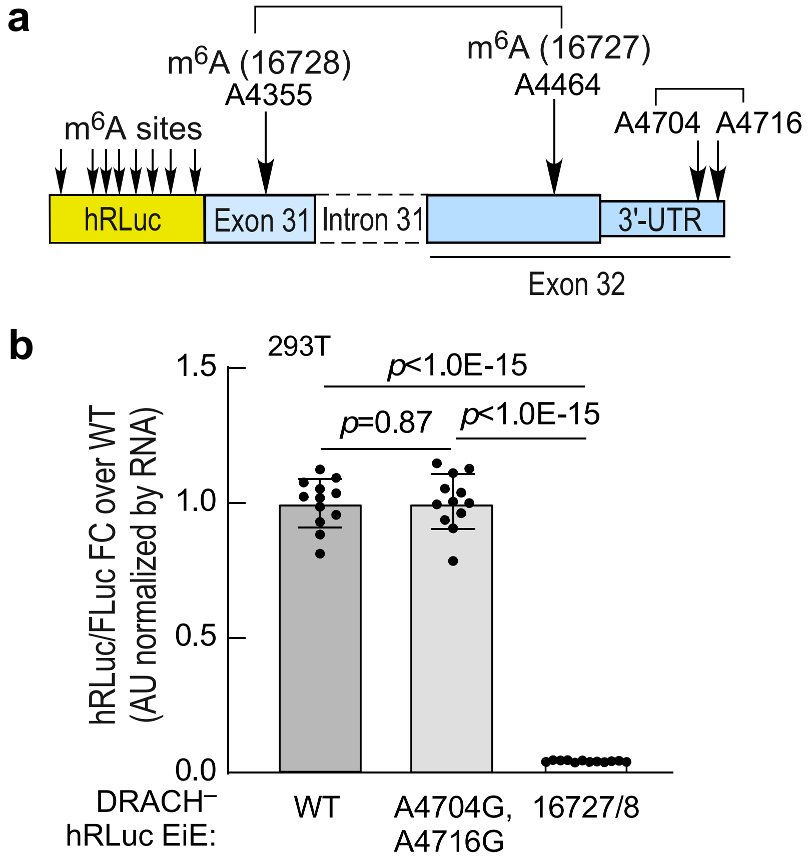

Supplementary Fig. 14 | DRACH sequences A4704 and A4716 are dispensable for EPRS1 reporter expression. a Schematic of pairwise mutations of EPRS1 DRACH sites in the background of DRACH⁻ hRLuc reporter. **b** Normalized expression of DRACH⁻ hRLuc reporter in HEK293T cells following pairwise mutation of m^{6}A sites A4704/A4716 or sites 16728/16727. Mean ± SD, n = 12 biological replicates; *p*-values are from unpaired two-tailed *t*-test.

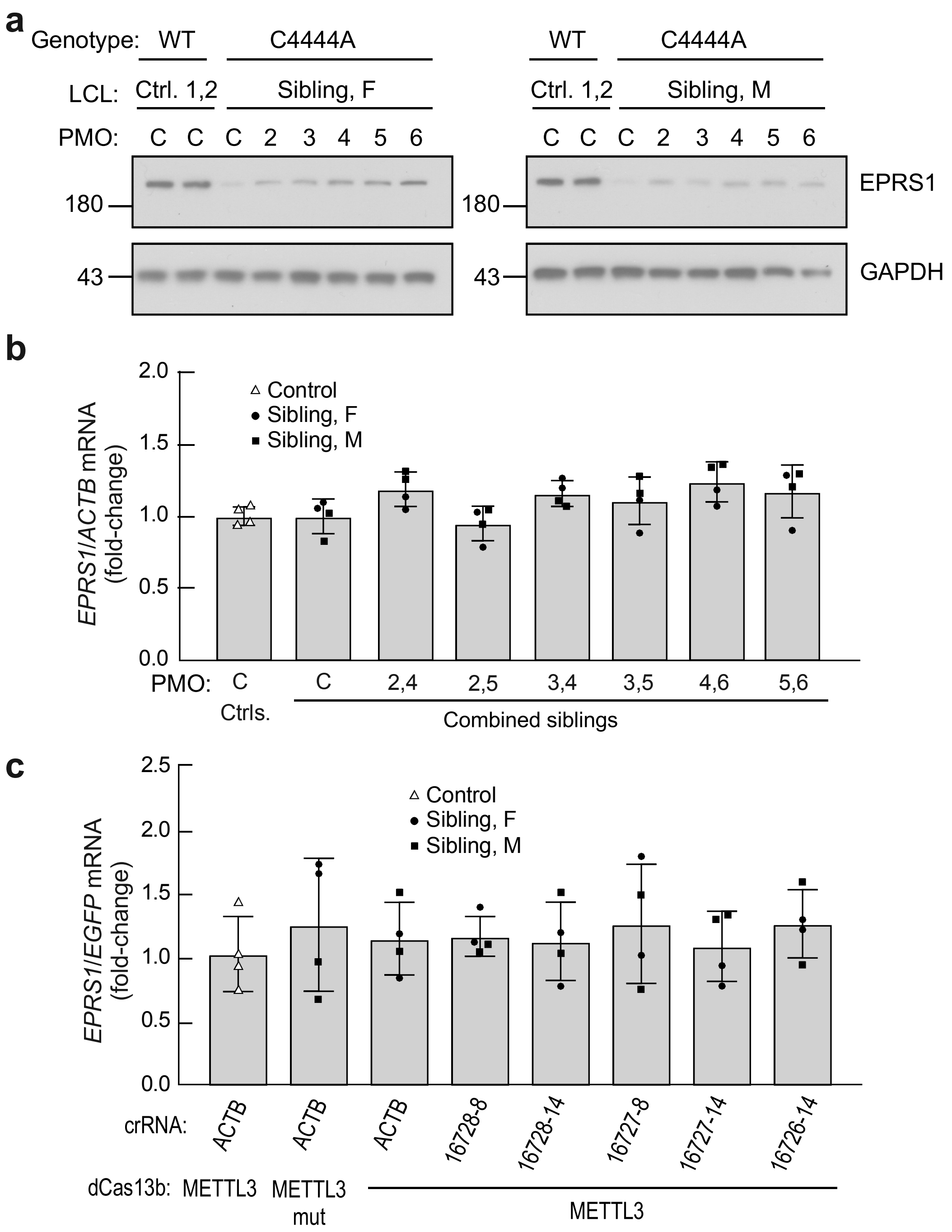

Supplementary Fig. 15 | Effect of PMOs on variant EPRS1 expression. a LCLs from controls, parents, and female (left) and male (right) siblings were incubated with control or singleton antisense PMOs, and EPRS1 expression determined by immunoblot. **b** Effect of pairwise addition of PMOs on EPRS1 mRNA in LCLs from controls and HLD siblings as determined by RT-qPCR. Mean ± SD, n = 4 male and female siblings pooled. **c** Effect of METTL3-dCas13b on *EPRS1* mRNA in LCLs from controls and HLD siblings as determined by RT-qPCR. Mean ± SD, n = 4 male and female siblings pooled.

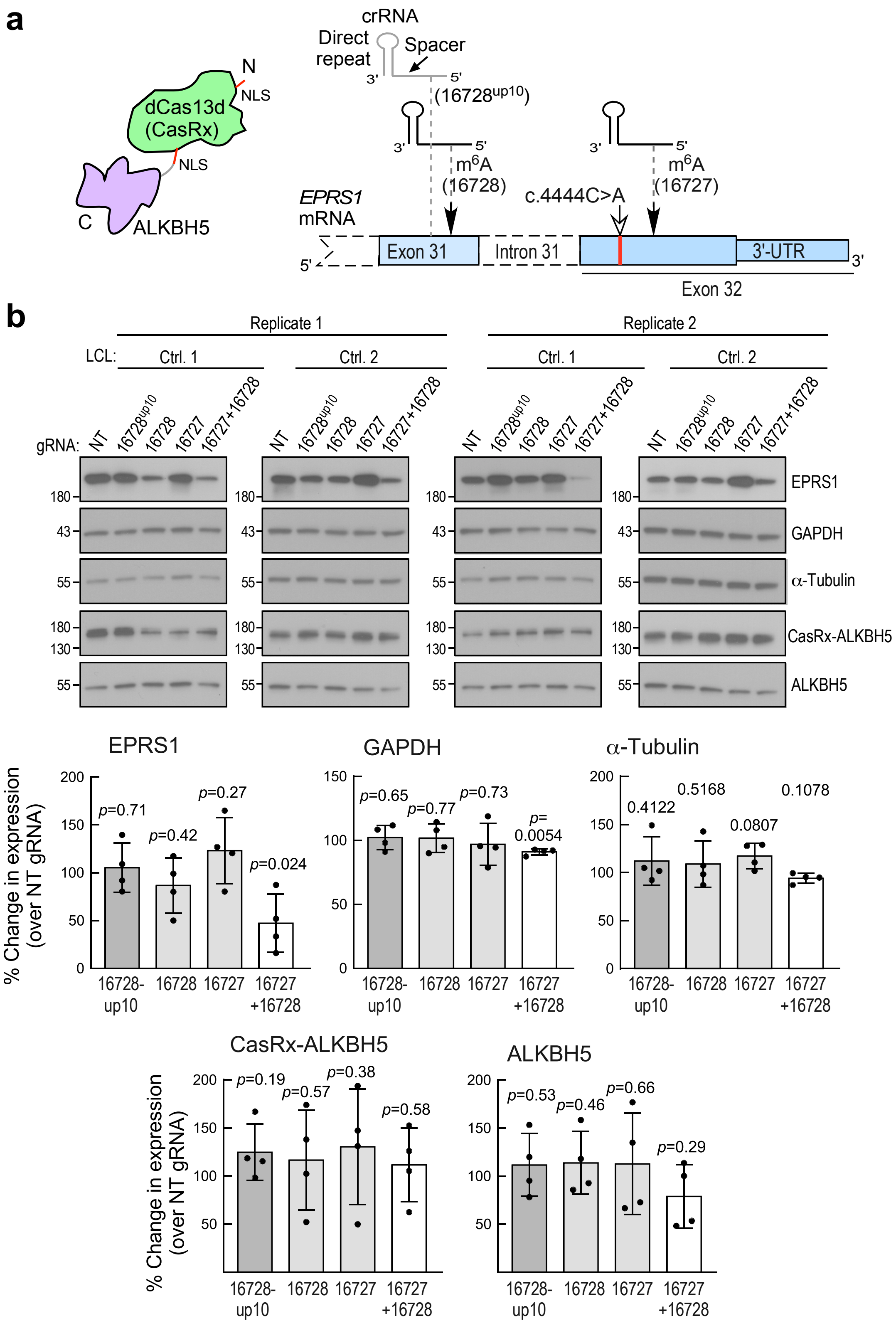

Supplementary Fig. 16 | Forced demethylation of sites 16728 and 16727 decreases EPRS1 expression in control LCLs. a Application of RfxdCas13d (dCasRx) fused to ALKBH5 demethylase and NLSs (left). crRNAs with direct repeats were targeted at m^{6}A sites 16728 and 16727 in *EPRS1* mRNA exons 31-32, or to a non-DRACH site 10-nt upstream to 16728 (16728^{up10}) (right). An EPRS1 non-targeting crRNA was used as control (not shown in schematic) **b** Control LCLs were nucleofected with crRNAs and chimeric dCasRx-ALKBH5 for 72 h, and protein expression determined by immuno-blot. Densitometric quantification represents mean ± SD, across 4 biological replicates, two each for two control LCLs; *p*-values from one-sample *t*-test (two-tailed) to measure statistically significant deviation from 100%.

a

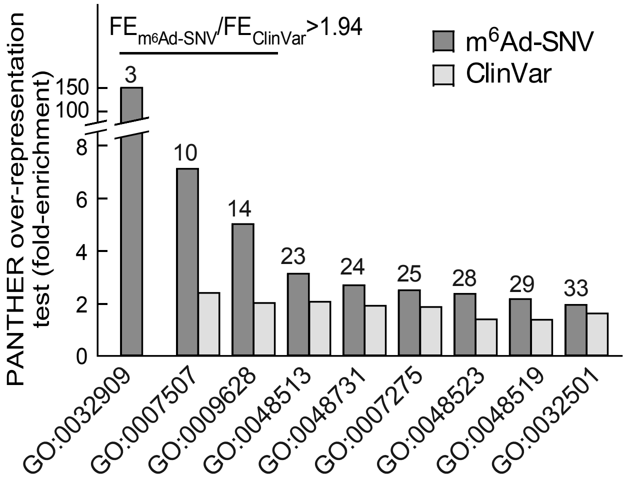

GO biological processes clustering terms:

GO:0032909	Regulation of TGFß2 production
GO:0007507	Heart development
GO:0009628	Response to abiotic stimulus
GO:0048513	Animal organ development
GO:0048731	System development
GO:0007275	Multicellular organism development
GO:0048523	Negative regulation of cellular process
GO:0048519	Negative regulation of biological process
GO:0032501	Multicellular organismal process

b

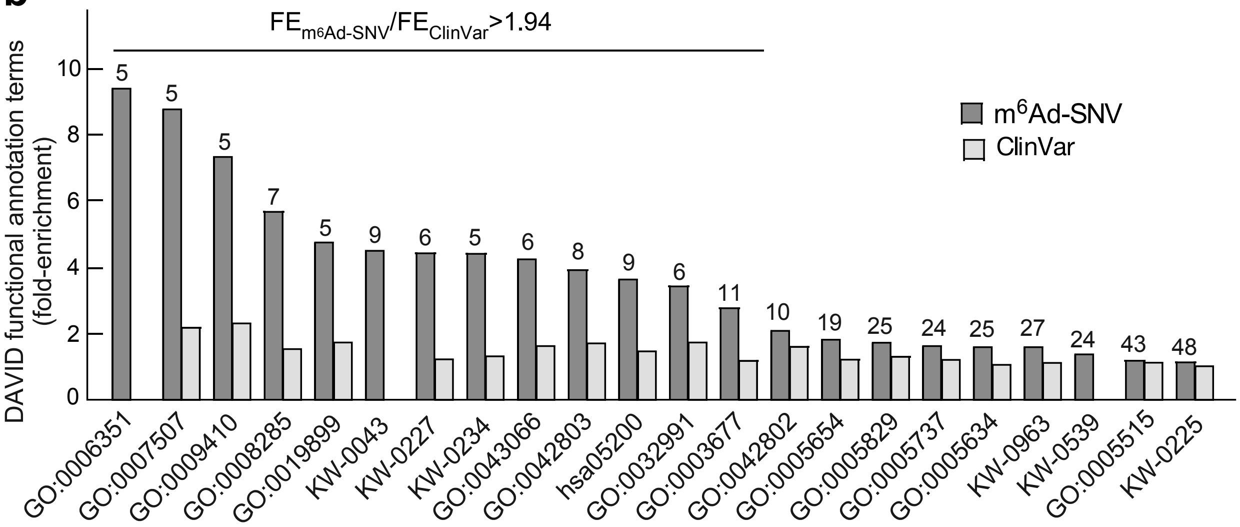

Annotation clustering terms:

GO:0006351	Transcription, DNA-templated
GO:0007507	Heart development
GO:0009410	Response to xenobiotic stimulus
GO:0008285	Negative regulation of cell proliferation
GO:0019899	Enzyme binding
KW-0043	Tumor suppressor
KW-0227	DNA damage
KW-0234	DNA repair
GO:0043066	Negative regulation of apoptotic process
GO:0042803	Protein homodimerization activity
hsa05200	Pathways in cancer
GO:0032991	Macromolecular complex
GO:0003677	DNA binding
GO:0042802	Identical protein binding
GO:0005654	Nucleoplasm
GO:0005829	Cytosol
GO:0005737	Cytoplasm
GO:0005634	Nucleus
KW-0963	Cytoplasm
KW-0539	Nucleus
GO:0005515	Protein binding
KW-0225	Disease variant

Supplementary Fig. 17 | Gene ontology analysis of m^{6}Ad-SNV-containing genes. a PANTHER statistical over-representation test of gene ontology (biological processes) for m^{6}Ad-SNV-containing genes plotted as fold-enrichment (FE, observed number/expected number), and fold-enrichment of same GO terms from all ClinVar genes. **b** DAVID functional annotation clustering for m^{6}Ad-SNV-containing genes, considered at a cut-off of $p < 0.04$. Enrichment of same terms from all ClinVar genes also plotted. All terms with fold fold-enrichment > 1.94 in the m^{6}Ad-SNV-affected genes compared to ClinVar genes are highlighted with vertical bar; the number of m^{6}Ad-SNV-containing genes for each term is shown above the relevant bar.

Supplementary Table 1 | Clinical characteristics of siblings with homozygous c.4444C>A; p.(Pro1482Thr) *EPRS1* variant

	**Proband (Sibling 1)**	**Sibling 2**
Sex (M/F)	Male	Female
Age at last review	18 years	16 years
Pregnancy	Unremarkable	Symmetric intrauterine growth restriction
Birth/gestational age	Uncomplicated spontaneous vaginal delivery at 39 weeks	Vaginal delivery after induction of labor at 37 weeks
Birth weight	2.9 kg (-2.3 SD)*	N/A
Birth length	46.5 cm (-3.6 SD)*	N/A
Birth head circumference	31.75 cm (-3.1 SD)*	N/A
Development	Severe global delays – sat at 8 months, walked at 34 months, spoke first words at 18 months. Deteriorated at age 4 years during a prolonged diarrheal illness, with onset of rotatory nystagmus and loss of ambulation	Severe global delays - sat at 8 months, walked at 21 months, spoke first words at 18 months. Developed rotatory nystagmus at 2 years during a prolonged diarrheal illness. She became non-ambulatory at 15 years
Current motor function	Fully dependent on a wheelchair for ambulation, with ongoing slow progression of spasticity and weakness	Primary mode of ambulation is a wheelchair but able to use a walker. Progressive bilateral lower limb spasticity and weakness
Current language function	Spoke with single words and had an estimated vocabulary of up to 25 words	Spoke with single words or short sentences
Current functional status	Dependent for all activities of daily living	Dependent for all activities of daily living
Current cognitive ability	Estimated to be at the level of <2 years, could follow simple one step commands	Able to name numbers, letters, colors, and spell her name
Seizures	None	Two “episodes” at age 12 but none since
Growth	Failure to thrive	Poor weight gain and constipation
Endocrine	No reported issues	Achieved menarche at 13 years, diagnosed with premature ovarian insufficiency at 15 years, treated with levonorgestrel-ethinyl estradiol
Current head circumference	18 years, 50 cm (-3.5 SD)	16 years, 47 cm (-4.8 SD)
Current height	18 years, 163.5 cm (5th centile)	16 years, 141 cm (-3.1 SD)
Current weight	18 years, 36.4 kg (-4 SD)	16 years, 31.4 kg (-4.2 SD)
Cranial nerve exam	Pronounced rotary nystagmus and saccadic smooth pursuit, normal extraocular movements, dysarthria, no bulbar signs	Pronounced rotary nystagmus and saccadic smooth pursuit, normal extraocular movements, left esotropia, dysarthria, no bulbar signs
Motor exam	Axial hypotonia and appendicular hypertonia with spasticity. Able to generate movements against gravity but not against resistance. Hyperreflexic with extensor plantar response and clonus bilaterally	Axial hypotonia and appendicular hypertonia with spasticity. Able to generate movements against gravity but clear weakness with added resistance. Hyperreflexic, with extensor plantar response and clonus bilaterally
Coordination/cerebellar exam	Head titubation, ataxia and action tremor of both hands, with pronounced dysmetria	Head titubation, ataxia and action tremor of both hands, with pronounced dysmetria
Brain MRI	Microcephaly with diffuse parenchymal volume loss, thin corpus callosum, and global hypomyelination, with progressive loss of myelination. MR spectroscopy was normal	Microcephaly and global hypomyelination with progressive myelin loss. MR spectroscopy was normal
Evoked potentials	Visual, somatosensory, and brainstem auditory evoked potentials at 4 years revealed delayed cortical responses	At 3 years, visual and somatosensory evoked potentials revealed delayed cortical responses, brainstem auditory evoked potentials were normal
Electroencephalogram	Not indicated	Abnormal background but no epileptiform discharges
Nerve conduction studies and electromyography	Normal at 4 and 13 years	Normal at 12 years

*Measured at 3 wk, growth parameters at birth not available, SD – standard deviation, N/A – not available

Supplementary Table 2 | Selected ancillary clinical investigations for siblings with homozygous c.4444C>A; p.(Pro1482Thr) *EPRS1* variant

	**Proband (Sibling 1)**	**Sibling 2**
Metabolic laboratory investigations	Ammonia, lactate, vitamin B12, vitamin E, homovanillic acid (HVA)/vanillylmandelic acid (VMA), plasma amino acids, very long chain fatty acids, phytanic acid, urine organic acids and mitochondrial respiratory chain complex enzyme activity ratios (in fibroblasts) all normal	Ammonia, lactate, vitamin B12, vitamin E, thyroid function tests, plasma amino acids, very long chain fatty acids, phytanic acid, urine organic acids all normal
SNP-based chromosomal microarray analysis	Maternally inherited 248 kb loss at 6q16.1 of no apparent clinical significance, as well as multiple genomic regions with absence of heterozygosity.	A maternally inherited 248 kb loss at 6q16.1, a 22 kb loss at 9q33.1, and a paternally inherited 388 kb gain at 10q26.13, which were of no apparent clinical significance. There were also multiple genomic regions with absence of heterozygosity.
Genetic testing	Targeted genetic testing for Pelizaeus-Merzbacher disease and related HLDs (sequencing and deletion/duplication analyses of *PLP1*, *GJC2*, *AIMP1*, and *HSPD1*) and sequencing and deletion testing of the mitochondrial genome was negative	Targeted genetic testing for Pelizaeus-Merzbacher disease and related HLDs (sequencing and deletion/duplication analyses of *PLP1*, *AIMP1*, *HSPD1*, and *FAM126A*) was negative. Sequencing and deletion testing of the mitochondrial genome was negative
Clinical exome sequencing	Homozygous for a novel predicted damaging missense variant c.4444C>A; p.(Pro1482Thr) in the gene *EPRS1* [NM_004446.2:]. Both parents and unaffected brother are heterozygous.	Homozygous for a novel predicted damaging missense variant c.4444C>A; p.(Pro1482Thr) in the gene *EPRS1* [NM_004446.2:]. Both parents and unaffected brother are heterozygous.

Supplementary Table 3 | Predicted m^{6}Ad-SNVs

Gene Symbol	Transcript_ID	ClinVar ID
ABCG8	NM_001357321.2	336092
AIP	NM_003977.4	485069
BBS10	NM_024685.4	310487
BMPR1A	NM_001406566.1	486801
BRAT1	NM_001350626.2	540173
BRAT1	NM_152743.4	540173
BRCA2	NM_001406722.1	409527
CACNB2	NM_201597.3	136649
CACNB2	NM_201597.3	698890
CBS	NM_001321072.1	413352
CBS	NM_001178009.3	413352
CDKN1B	NM_004064.5	404269
CDKN1B	NM_004064.5	536845
CFAP418	NM_177965.4	31194
CFAP418	NM_001363260.1	31194
CLN5	NM_001366624.2	527741
CTNS	NM_001374496.1	322845
CTNS	NM_001031681.3	322846
DKC1	NM_001142463.3	580161
DNAI1	NM_012144.4	454834
EARS2	NM_001308211.1	318541
ENSG00000256591	NM_017841.4	463828
ENSG00000267618	NM_001142571.2	141707
ENSG00000267618	NM_001142571.2	630954
ENSG00000273269	NM_001305626.1	458192
FBN1	NM_001406716.1	519710
FGD4	NM_001370298.3	543382
FH	NM_000143.4	460345
FHL1	NM_001159702.3	11559
FKTN	NM_001351498.2	36137
FKTN	NM_001198963.2	364489
FKTN	NM_001351498.2	459218
FKTN	NM_001198963.2	459218
GLE1	NM_001003722.2	6465
GNRHR	NM_001012763.2	349452
GUSB	NM_000181.4	92588
HNRNPA1	NM_002136.4	135603
HYCC1	NM_032581.4	359768
IDUA	NM_000203.5	100723
IFT74	NM_001099223.3	254276
IRF2BP2	NM_001077397.1	446216
IRF2BP2	NM_182972.3	446216
KCTD7	NM_153033.5	469108
KCTD7	NM_001167961.2	469108
KIRREL3	NM_032531.4	2888
MCL1	NM_182763.3	623464
MEN1	NM_001407150.1	305312
MEN1	NM_001407152.1	305312
MSH2	NM_001406648.1	90999
MSH2	NM_001406648.1	91000
MSH2	NM_001406648.1	387732
MSH2	NM_001406657.1	489918
MSMO1	NM_001017369.3	222976
NR4A2	NM_006186.4	331658
PALB2	NM_001407304.1	126741
PALB2	NM_001407309.1	126741
PALB2	NM_001407304.1	126745
PALB2	NM_001407309.1	126745
PALB2	NM_001407304.1	141936
PALB2	NM_001407309.1	141936
PALB2	NM_001407304.1	182776
PALB2	NM_001407309.1	182776
PALB2	NM_001407304.1	182777
PALB2	NM_001407309.1	182777
PALB2	NM_001407310.1	229790
PALB2	NM_001407304.1	234009
PALB2	NM_001407309.1	234009
PALB2	NM_001407304.1	378321
PALB2	NM_001407309.1	378321
PALB2	NM_001407310.1	460993
PALB2	NM_001407304.1	484208
PALB2	NM_001407309.1	484208
PALB2	NM_001407304.1	530116
PALB2	NM_001407310.1	530116
PALB2	NM_001407309.1	530116
PALB2	NM_001407310.1	530122
PALB2	NM_001407304.1	628361
PALB2	NM_001407309.1	628361
PLEKHG5	NM_001265594.3	581116
PMS2	NM_001406877.1	480318
PMS2	NM_001406875.1	480318
PMS2	NM_001406871.1	480318
PRICKLE1	NM_001144881.2	1291049
SDHB	NM_003000.3	412459
SIK1	NM_173354.5	476102
SMAD3	NM_001407012.1	213777
SMAD4	NM_001407041.1	492457
SMAD4	NM_001407041.1	630655
SP110	NM_080424.4	334890
SP110	NM_001378446.1	334890
SP110	NM_001378446.1	577709
TGFBI	NM_000358.3	350898
THRB	NM_001252634.2	12564
THRB	NM_001374827.1	12564
TIMM8A	NM_004085.4	11321
TSC2	NM_001406686.1	49370
TSC2	NM_001370405.1	49370
TSC2	NM_001406686.1	65302
TSC2	NM_001370405.1	65302
TSC2	NM_001406686.1	468159
TSC2	NM_001370405.1	468159
TSC2	NM_001406686.1	486616
TSC2	NM_001370405.1	486616
TSC2	NM_001406686.1	535891
TSC2	NM_001370405.1	535891
TSC2	NM_001406686.1	536004
TSC2	NM_001370405.1	536004
TSC2	NM_001406686.1	655006
TSC2	NM_001370405.1	655006
TSC2	NM_001406686.1	1006099
TSC2	NM_001370405.1	1006099
TSC2	NM_001406686.1	1019286
TSC2	NM_001370405.1	1019286
TXNRD2	NM_001352303.2	264270
VCL	NM_014000.3	487634
VHL	NM_198156.3	2224
VHL	NM_000551.4	2224

Supplementary Table 4 | Reagents, plasmids, oligonucleotides, and software

REAGENT or RESOURCE	SOURCE	IDENTIFIER
**Antibodies** *(WB: Western blot, RIP: RNA Immunoprecipitation, MeRIP-Methylated RNA Immunoprecipitation)*		
Rabbit polyclonal anti-EPRS1 linker: WB (1:100000)	Lerner Research Institute (LRI) Core	KD validated and tested for WB in[1,2]
Rabbit polyclonal anti-KARS1: WB (1:2000)	Proteintech	14951-1-AP, RRID:AB_2128158, KD/KO validated by seller
Rabbit polyclonal anti-LARS1: WB (1:1000)	Proteintech	21146-1-AP, RRID:AB_10733878, KD/KO validated by seller
Rabbit polyclonal anti-IARS1: WB (1:1000)	Proteintech	26942-1-AP, RRID:AB_2880693, tested for WB in multiple human cell lines by seller
Rabbit polyclonal anti-EEF1E1 (AIMP3): WB (1:1000)	Proteintech	10805-1-AP, RRID:AB_2097140, KD/KO validated by seller
Mouse monoclonal anti-FLAG [M2]: IP (2 μg per mg input protein), WB (1:2000)	Millipore Sigma	F1804, RRID:AB_262044, tag-specificity validated by seller
Mouse monoclonal anti-SARS1 [C-2]: WB (1:1000)	Santa Cruz	sc-271032, RRID:AB_10610742, tested for WB in multiple human cell lines by seller
Rabbit monoclonal anti-METTL3 [EPR18810]: WB (1:1000-1:4000)	Abcam	ab195352, AB_2721254, KO validated by seller
Rabbit polyclonal anti-YTHDC1/YT521 Antibody, affinity purified: WB (1:1000-1:2000), RIP (2 μg per mg input protein)	Bethyl Labs	A305-096A, RRID:AB_2631491, KO and RIP validated in[3]
Rabbit polyclonal anti-YTHDC1 Antibody: WB, RIP (2 μg per mg input protein)	Proteintech	14392-1-AP, RRID:AB_2878052, KD/KO and RIP validated by seller
Rabbit polyclonal anti-YTHDC2 Antibody: WB, RIP (2 μg per mg input protein)	Proteintech	27779-1-AP, RRID:AB_2880970, KD/KO validated by seller and RIP validated in[4]
Rabbit polyclonal anti-YTHDF1 Antibody: WB (1:2000-1:8000), RIP (2 μg per mg input protein)	Proteintech	17479-1-AP, RRID:AB_2217473, KD/KO and RIP validated by seller
Rabbit polyclonal anti-YTHDF2 Antibody: WB (1:2000-1:4000), RIP (2 μg per mg input protein)	Proteintech	24744-1-AP, RRID:AB_2687435, KD/KO and RIP validated by seller
Rabbit polyclonal anti-YTHDF3 Antibody: WB (1:4000-1:8000), RIP (2 μg per mg input protein)	Proteintech	25537-1-AP, RRID:AB_2847817, KD/KO and RIP validated by seller
Mouse monoclonal anti-Nuclear Matrix Protein p84 antibody [5E10]: WB (1:2000)	GeneTex	GTX70220, RRID:AB_372637, KD/KO validated by seller
Rabbit monoclonal anti-NXF1 [EPR8009]: WB (1:1000-1:2000)	Abcam	ab129160, RRID:AB_11142853, KD validated in[5]
Rabbit polyclonal anti-GANP Antibody: WB (1:1000)	Proteintech	11054-1-AP, RRID:AB_2266280, KD/KO validated by seller
Rabbit polyclonal anti-CRM1 Antibody: WB (1:4000-1:20000)	Novus	NB100-79802, RRID:AB_1108371, KD/KO validated by seller
Rabbit polyclonal anti-IPMK Antibody [N1C2]: WB (1:500-1:1000)	GeneTex	GTX104954, RRID:AB_1950594, KD validated by seller
Rabbit polyclonal anti-RARS1: WB (1:2000)	Proteintech	27344-1-AP, RRID:AB_2880849, KD/KO validated by seller for WB in human cell lines.

REAGENT or RESOURCE	SOURCE	IDENTIFIER
Mouse monoclonal anti-NARS1 [G-8]: WB (1:1000)	Santa Cruz	sc-271059, RRID:AB_10613799, Seller tested for WB in multiple human cell lines
Rabbit polyclonal anti-EMAP II (AIMP1): WB (1:1000)	Proteintech	11091-1-AP, RRID:AB_2182590, KD/KO validated by seller for WB in human cell lines.
Rabbit polyclonal anti-METTL14: WB (1:2000)	Proteintech	26158-1-AP, RRID:AB_2800447, KD/KO validated by seller for WB in human cell lines.
Rabbit polyclonal anti-ALKBH5: WB (1:2000-1:4000)	Proteintech	16837-1-AP, RRID:AB_2242665, KD/KO validated by seller for WB in human cell lines.
Mouse monoclonal anti-Nuclear Matrix Protein p84 antibody [5E10]: WB (1:2000)	GeneTex	GTX70220, RRID:AB_372637, KD/KO validated by seller for WB in human cell lines.
Mouse monoclonal anti-β-actin-HRP: WB (1:10000-1:20000)	Proteintech	HRP-60008, RRID:AB_2819183, Seller tested for WB in multiple human and mouse cell lines.
Mouse monoclonal anti-α-tubulin-HRP: WB (1:10000-1:20000)	Proteintech	HRP-66031, RRID:AB_2687491, Seller tested for WB in multiple human and mouse cell lines.
Rabbit polyclonal anti-m^6A antibody, affinity purified: MeRIP (1 μg per 30 μg total RNA)	Synaptic Systems	202 003, RRID:AB_2279214, KD validated by seller.
Mouse monoclonal anti-GAPDH-HRP: WB (1:10000-1:20000)	Proteintech	HRP-60004, RRID:AB_2737588, Seller tested for WB in multiple human and mouse cell lines.
Rabbit Monoclonal IgG XP Isotype control [DA1E]: IP	Cell Signaling Technology	3900, RRID:AB_1550038
Normal Mouse IgG Control: IP	Santa Cruz	sc-2025, RRID:AB_737182
Donkey Anti-Rabbit-IgG-HRP: WB	Cytiva LifeSciences	NA934, RRID:AB_772206
Sheep Anti-Mouse-IgG-HRP: WB	Cytiva LifeSciences	NA931, RRID:AB_772210
Rabbit polyclonal anti-METTL 16: WB (1:500)	Proteintech	19924-1-AP, RRID:AB_10639364, KD/KO validated in [6].
Rabbit polyclonal anti-FTO: WB (!:2000)	Proteintech	27226-1-AP, RRID:AB_2880809, KD/KO validated by seller for WB in human cell lines.
**Bacterial and viral strains**		
Escherichia coli DH5-α	NEB	C2987I, C2988J
Escherichia coli BL21-CodonPlus (DE3)-RIPL	Agilent	230280
Epstein Barr Virus B95-8 (ATCC CRL-1612)	Accegen	ABC-TC0065
**Experimental models: Cell lines**		
Human: control LCLs 1,2	Genomic Medicine Biorepository LRI	N/A
Human: Control LCL 3, Parent LCLs, Sibling LCLs	This paper	N/A
Human: 293 F (HEK293F)	Thermo Fisher	11625019
Human: HEK293T	LRI Cell Culture Core (ATCC: CRL-3216)	N/A

REAGENT or RESOURCE	SOURCE	IDENTIFIER
Human: U87-MG	LRI Cell Culture Core (ATCC: HTB-14)	N/A
**Media and reagents**		
BD Vacutainer Specialty Tubes with ACD solution A	BD Biosciences	364606
Protein A Dynabeads	Thermo Fisher	10008D
Protein G Dynabeads	Thermo Fisher	10009D
rRNAsin	Promega	N2511
RNaseOUT	Thermo Fisher	10777019
Proteinase K	Thermo Fisher	AM2546
RNase-free DNase set	Qiagen	79254
TRIzol	Thermo Fisher	15596018
TRIzol LS	Thermo Fisher	10296028
RIPA buffer	Millipore-Sigma	R0278
Passive Lysis Buffer	Promega	E1941
^{35}S-L-Methionine-Cysteine	Perkin Elmer	NEG77200
^{14}C-Proline	Perkin Elmer	NEC285E050
^{14}C-Glutamic acid	Perkin Elmer, American Radiolabeled Chemicals	NEC290E050, ARC-0165A
Total yeast tRNA	Millipore-Sigma/Roche	10109495001
Endo-Porter PEG	Gene Tools	OT-EP-PEG-1
Ambion RNase-free buffer kit	Thermo Fisher	AM9010
Ficoll-Paque Plus density gradient media	Cytiva LifeSciences	17144002
RPMI 1640, with L-glutamine	Wisent Bioproducts	350-000-CL
RPMI 1640, with L-glutamine	LRI Cell Culture Core	10-500 RPMI
DMEM, high glucose, with L-glutamine and sodium pyruvate	LRI Cell Culture Core	11-500 DMEM
EMEM, with non-essential amino acids; 2 mM L-glutamine; 1 mM sodium pyruvate; 1.5 g/L sodium bicarbonate	LRI Cell Culture Core	99BJ500CUST (ATCC 30-2003)
OptiMEM-I Reduced Serum Medium	Thermo Fisher	31985062
HyClone characterized fetal bovine serum, Canadian origin	Cytiva LifeSciences	SH30396.03
Fetal bovine serum (heat-inactivated)	Gemini Biosciences	110-106
Fetal bovine serum (non-heat inactivated)	Sigma	F0926
Lipofectamine 2000	Thermo Fisher	11668019
Lipofectamine RNAiMAX	Thermo Fisher	13778030
T4 DNA Ligase	NEB	M0202S
Gel filtration standards	Bio-Rad	1511901
HisTrap HP column	Cytiva LifeSciences	17524701
Superdex 200 10/300 GL column	Cytiva LifeSciences	28990944
Dialyzed FBS	Thermo Fisher	A3382001
Anti-DYKDDDDK G1 affinity resin	GenScript	L00432

REAGENT or RESOURCE	SOURCE	IDENTIFIER
3X FLAG peptide	Millipore Sigma	F4799
Bst 2.0 DNA polymerase	NEB	M0537S
SplintR Ligase	NEB	M0375S
Recombinant FTO demethylase	ActiveMotif	31572
**Critical commercial assays and kits**		
SuperScript III One-Step RT-PCR System	Thermo Fisher	12574018
QuikChange II site directed mutagenesis kit	Agilent	200523
Q5 site directed mutagenesis Kit	New England Biolabs	E0554S
HiFi DNA Assembly Kit	New England Biolabs	E2621S
AgPath-ID One Step RT-PCR Kit	Thermo Fisher	AM1005
RNeasy Mini Kit	Qiagen	74004
Luciferase assay system	Promega	E1500
Renilla Glo luciferase assay	Promega	E2710
PARIS Kit	Thermo Fisher	AM1921
Cell Line Nucleofector Kit V	Lonza	VCA-1003
PowerUp SYBR Green Master Mix	Thermo Fisher	A25742
**Oligonucleotides: Primers, primer-probe sets, SELECT-qPCR probes and primers, siRNAs and PMOs**		
Renilla luciferase (hRLuc) forward primer: 5' TCCAGATTGTCCGCAACTAC 3' (Sense); hRLuc reverse primer: 5' CTTCTTAGCTCCCTCGACAATAG 3' (AntiSense); hRLuc Probe: 5' 6-FAM/CCAGCGACG/ZEN/ATCTGCCTA AGATGTT/3' IABkFQ (Sense)	Integrated DNA Technologies	Customized PrimeTime probe set,[2]
Q0–QI–T primer: 5' CCAGTGAGCAGAGTGACGAGG¬ACT CGAGCTCAAGCTTTTTTTTTTTTTTTTT T 3'	Integrated DNA Technologies	Customized primer, this paper
Q0: 5' CCAGTGAGCAGAGTGACG 3'	Integrated DNA Technologies	Customized primer, this paper
QI: 5' GAGGACTCGAGCTCAAGC 3'	Integrated DNA Technologies	Customized primer, this paper
EPRS1 Taqman Assay set (Hs01114843_m1)	Thermo Fisher	4448892
ACTB Taqman Assay set (Hs01060665_g1)	Thermo Fisher	4331182
18S rRNA Taqman Assay set (Hs99999901_s1)	Thermo Fisher	4331182
18S rRNA Taqman Assay set (Hs03003631_g1)	Thermo Fisher	4331182
GAPDH Taqman Assay set (Hs02758991_g1)	Thermo Fisher	4331182
GFP Taqman Assay set (Mr04329676_mr)	Thermo Fisher	4331182

REAGENT or RESOURCE	SOURCE	IDENTIFIER
Luciferase (FLuc) Taqman Assay set (Mr03987587_mr)	Thermo Fisher	4331182
Silencer Select Negative control #1	Thermo Fisher	4390483
Silencer Select METTL3 siRNA, AssayID: s32143	Thermo Fisher	4392420
Silencer Select YTHDC1 siRNA, AssayID: s40756 (siYTHDC1-1)	Thermo Fisher	4392420
Silencer Select YTHDC1 siRNA, AssayID: s40758 (siYTHDC1-2)	Thermo Fisher	4392420
Silencer Select YTHDC1 siRNA, AssayID: n372359 (siYTHDC1-3)	Thermo Fisher	4390771
Silencer Select YTHDF1 siRNA, AssayID: s29745	Thermo Fisher	4392420
Silencer Select YTHDF2 siRNA, AssayID: s28148	Thermo Fisher	4392420
Silencer Select YTHDF3 siRNA, AssayID: s48464	Thermo Fisher	4392420
Silencer Select YTHDC2 siRNA, AssayID: s35020	Thermo Fisher	4392420
Silencer Select NXF1 siRNA, AssayID: s20532	Thermo Fisher	4392420
Silencer Select MCM3AP (GANP) siRNA, AssayID: s16987	Thermo Fisher	4392420
Silencer Select XPO1 (CRM1) siRNA, AssayID: s14937	Thermo Fisher	4392420
Silencer Select IPMK siRNA, AssayID: s48412	Thermo Fisher	4392420
Silencer Select EPRS1 siRNA, AssayID: s4767	Thermo Fisher	4392420
PMO2: 5' ATTTCCCCACAGAATGGAATCTGAA 3'	Gene Tools	This paper
PMO3: 5' CAATTTCCCCACAGAATGGAATCTG 3'	Gene Tools	This paper
PMO4: 5' TCACAGAGTGGTTTGAAGGTGATGC 3'	Gene Tools	This paper
PMO5: 5' ACAGAGTGGTTTGAAGGTGATGCAA 3'	Gene Tools	This paper
PMO6: 5' CCTCACTCCCCACAGAATGGAATCT 3'	Gene Tools	This paper
Standard Control morpholino: 5' CCTCTTACCTCAGTTACAATTTATA 3'	Gene Tools	PCO-StandardControl-100
SELECT EPRS1 A4355 down probe: 5'phosCAATTTCCCCACAGAATGGcag aggctgagtcgctgcat 3'	Integrated DNA Technologies	Customized primer, this paper
SELECT EPRS1 A4355 up probe: 5'tagccagtaccgtagtgcgtgTTTTTGATCC AGTCCTCACAG 3'	Integrated DNA Technologies	Customized primer, this paper
SELECT EPRS1 A4364 down probe: 5'phosCCTCACAGTCAATTTCCCCcag aggctgagtcgctgcat 3'	Integrated DNA Technologies	Customized primer, this paper
SELECT EPRS1 A4364 up probe: 5'tagccagtaccgtagtgcgtgGCAGTGGTC TTTTTGATCCAG 3'	Integrated DNA Technologies	Customized primer, this paper

REAGENT or RESOURCE	SOURCE	IDENTIFIER
SELECT EPRS1 A4378 down probe: 5'phosCTTTTTGATCCAGTCCTCACAcagaggctgagtcgctgcat 3'	Integrated DNA Technologies	Customized primer, this paper
SELECT EPRS1 A4378 up probe: 5'tagccagtaccgtagtgcgtgTCTTGATCCCTGGCAGTGG 3'	Integrated DNA Technologies	Customized primer, this paper
SELECT EPRS1 A4404 down probe: 5'phosTCAAGATCTTGATCCCTGGCAcagaggctgagtcgctgcat 3'	Integrated DNA Technologies	Customized primer, this paper
SELECT EPRS1 A4404 up probe: 5'tagccagtaccgtagtgcgtgCCATGGATGGAGCACCAGG 3'	Integrated DNA Technologies	Customized primer, this paper
SELECT EPRS1 A4452 downWT probe: 5'phosTTGAAGGGGATGCAAAGGCTcagaggctgagtcgctgcat 3' (for control LCLs only)	Integrated DNA Technologies	Customized primer, this paper
SELECT EPRS1 A4452 downSNV probe: 5'phosTTGAAGGTGATGCAAAGGCTcagaggctgagtcgctgcat 3' (for patient LCLs only)	Integrated DNA Technologies	Customized primer, this paper
SELECT EPRS1 A4452 up probe: 5'tagccagtaccgtagtgcgtgGGCTGCAGTTCACAGAGTGG 3'	Integrated DNA Technologies	Customized primer, this paper
SELECT EPRS1 A4464 downWT probe: 5'phosTCACAGAGTGGTTTGAAGGGGcagaggctgagtcgctgcat 3' (for control LCLs only)	Integrated DNA Technologies	Customized primer, this paper
SELECT EPRS1 A4464 downSNV probe: 5'phosTCACAGAGTGGTTTGAAGGTGcagaggctgagtcgctgcat 3' (for patient LCLs only)	Integrated DNA Technologies	Customized primer, this paper
SELECT EPRS1 A4464 up probe: 5'tagccagtaccgtagtgcgtgATTTGGCTCCAGGCTGCAG 3'	Integrated DNA Technologies	Customized primer, this paper
SELECT EPRS1 A4499 down probe: 5'phosTCTTGCCACAGACACATTTGcagaggctgagtcgctgcat 3'	Integrated DNA Technologies	Customized primer, this paper
SELECT EPRS1 A4499 up probe: 5'tagccagtaccgtagtgcgtgAGGTGTAGTACTTGGCAGGG 3'	Integrated DNA Technologies	Customized primer, this paper
SELECT EPRS1 A4614 down probe: 5'phosCTGTATCTGAGAAGATACTAATATCAATGCcagaggctgagtcgctgcat 3'	Integrated DNA Technologies	Customized primer, this paper
SELECT EPRS1 A4614 up probe: 5'tagccagtaccgtagtgcgtgAGAACTTTTACTTTTTAAAAAATCATAAAACGG 3'	Integrated DNA Technologies	Customized primer, this paper
SELECT EPRS1 A4666 down probe: 5'phosCCTGTGTGACTTCATTTTAGAACTTTTACcagaggctgagtcgctgcat 3'	Integrated DNA Technologies	Customized primer, this paper
SELECT EPRS1 A4666 up probe: 5'tagccagtaccgtagtgcgtgACTGTTAACTTAGGCATAAGAATAATTG 3'	Integrated DNA Technologies	Customized primer, this paper
SELECT EPRS1 A4690 down probe: 5'phosTAACTTAGGCATAAGAATAATTGTCCTcagaggctgagtcgctgcat 3'	Integrated DNA Technologies	Customized primer, this paper

REAGENT or RESOURCE	SOURCE	IDENTIFIER
SELECT EPRS1 A4690 up probe: 5'tagccagtaccgtagtgcgtgTTTACAGAAAAGTCTTTTATCCACTG 3'	Integrated DNA Technologies	Customized primer, this paper
SELECT EPRS1 A4704 down probe: 5'phosCTTTTATCCACTGTTAACTTAGGCATAAGcagaggctgagtcgctgcat 3'	Integrated DNA Technologies	Customized primer, this paper
SELECT EPRS1 A4704 up probe: 5'tagccagtaccgtagtgcgtgTTATTACTGGAGTTGTTTACAGAAAAG 3'	Integrated DNA Technologies	Customized primer, this paper
SELECT EPRS1 A4716 down probe: 5'phosTTACAGAAAAGTCTTTTATCCACTGTcagaggctgagtcgctgcat 3'	Integrated DNA Technologies	Customized primer, this paper
SELECT EPRS1 A4716 up probe: 5'tagccagtaccgtagtgcgtgTTAGTTCATGATATTTATTACTGGAGTTG 3'	Integrated DNA Technologies	Customized primer, this paper
SELECT EPRS1 A4740 down probe: 5'phosTCATGATATTTATTACTGGAGTTGcagaggctgagtcgctgcat 3' *[non-specific amplification]*	Integrated DNA Technologies	Customized primer, this paper
SELECT EPRS1 A4740 up probe: 5'tagccagtaccgtagtgcgtgTTTTTTTTTTTTTTTTTTTTTTTTTTTAG 3' [*non-specific amplification]*	Integrated DNA Technologies	Customized primer, this paper
SELECT EPRS1 A4349 down probe: 5'phosCCCCACAGAATGGAATCTGcagaggctgagtcgctgcat 3' (non-DRACH control)	Integrated DNA Technologies	Customized primer, this paper
SELECT EPRS1 A4349 up probe: 5'tagccagtaccgtagtgcgtgATCCAGTCCTCACAGTCAATT 3' (non-DRACH control)	Integrated DNA Technologies	Customized primer, this paper
SELECT EPRS1 A4469 down probe: 5'phosGCAGTTCACAGAGTGGTTTGcagaggctgagtcgctgcat 3' (non-DRACH control)	Integrated DNA Technologies	Customized primer, this paper
SELECT EPRS1 A4469 up probe: 5'tagccagtaccgtagtgcgtgAGACACATTTGGCTCCAGGC 3' (non-DRACH control)	Integrated DNA Technologies	Customized primer, this paper
SELECT 28S rRNA m^{6}A4190 down probe: 5'phosTACCGTTTGACAGGTGTAcagaggctgagtcgctgcat 3'	Integrated DNA Technologies	Customized primer, this paper
SELECT 28S rRNA m^{6}A4190 up probe: 5'tagccagtaccgtagtgcgtgCGCCTTAGGACACCTGCG 3'	Integrated DNA Technologies	Customized primer, this paper
SELECT qPCR Forward primer: 5' ATGCAGCGACTCAGCCTCTG 3'	Integrated DNA Technologies	[7]
SELECT qPCR Reverse primer: 5' TAGCCAGTACCGTAGTGCGTG 3'	Integrated DNA Technologies	[7]
**Recombinant DNA**		
Plasmid: pCMV10-3xFLAG-EPRS1	[8]	N/A
Plasmid: pCMV10-3xFLAG-EPRS1-Pro1482Thr	This paper	N/A
Plasmid: PF0721 (hRLuc-EIE)	This paper	N/A

REAGENT or RESOURCE	SOURCE	IDENTIFIER
Plasmid: PF0722 (hRLuc-EIE c.4444C>A)	This paper	N/A
Plasmid: PF0701 (hRLuc-EE)	This paper	N/A
Plasmid: PF0702 (hRLuc-EE c.4444C>A)	This paper	N/A
Plasmid: PF0741 (hRLuc-E-*RBGI*-EIE)	This paper	N/A
Plasmid: PF0742 (hRLuc-E-*RBGI*-EIE c.4444C>A)	This paper	N/A
Plasmid: PF0921 (hRLuc-EcIE)	This paper	N/A
Plasmid: PF0922 (hRLuc-EcIE c.4444C>A)	This paper	N/A
Plasmid: PF0941 (hRLuc-E-*RBGI*-EcIE)	This paper	N/A
Plasmid: PF0942 (hRLuc-E-*RBGI*-EcIE c.4444C>A)	This paper	N/A
Plasmid: PF0821 (cI-hRLuc-EIE)	This paper	N/A
Plasmid: PF0822 (cI-hRLuc-EIE c.4444C>A)	This paper	N/A
Plasmid: PF0801 (cI-hRLuc-EE)	This paper	N/A
Plasmid: PF0802 (cI-hRLuc-EE c.4444C>A)	This paper	N/A
Plasmid: DLRL0721 (DRACH^{-} hRLuc-EIE)	This paper	N/A
Plasmid: DLRL0722 (DRACH^{-} hRLuc-EIE c.4444C>A)	This paper	N/A
Plasmid: DLRL0721 16728-16727 double mutant	This paper	N/A
Plasmid: DLRL0721 16727-16726 double mutant	This paper	N/A
Plasmid: DLRL0721 16728-16726 double mutant	This paper	N/A
Plasmid: DLRL0722 comp. U (G4347U)	This paper	N/A
Plasmid: DLRL0722 comp. C (G4347C)	This paper	N/A
Plasmid: DLRL0722 comp. A (G4347A)	This paper	N/A
Plasmid: DLRL0721 C4444G, G4347C	This paper	N/A
Plasmid: DLRL0721 C4446G, G4345C	This paper	N/A
Plasmid: DLRL0721 C4445G, G4346C	This paper	N/A
Plasmid: DLRL0721 C4445A, G4346T	This paper	N/A
Plasmid: DLRL0721 (Gln1444Ile, Ile1445Gln, and Pro1446)	This paper	N/A
Plasmid: DLRL0722 (Gln1444Ile, Ile1445Gln, and Pro1446) "16727 unmask"	This paper	N/A
Plasmid: DLRL0721 (Ile1451Ile and Ile1481His)	This paper	N/A
Plasmid: DLRL0722 (Ile1451Ile and Ile1481His) "16728 unmask"	This paper	N/A
Plasmid: DLRL0721 A4704G, A4716G	This paper	N/A
Plasmid: pGL3-control (Firefly)	Promega	E1741
Plasmid: pCMV-dCas13-M3nls	Addgene	RRID:Addgene_155366
Plasmid: pCMV-dCas13-inactive M3nls	Addgene	RRID:Addgene_157854

REAGENT or RESOURCE	SOURCE	IDENTIFIER
Plasmid: pU6-PspCas13b-gRNA-Actb1216	Addgene	RRID:Addgene_155368
Plasmid: pU6-PspCas13b-gRNA-EPRS1-16728-8 (spacer: 5' CCACAGAATGGAATCTGAACAATCTTTCCA 3')	This paper	N/A
Plasmid: pU6-PspCas13b-gRNA-EPRS1-16728-14 (spacer: 5' AATGGAATCTGAACAATCTTTCCAGAATCT 3')	This paper	N/A
Plasmid: pU6-PspCas13b-gRNA-EPRS1-16727-8 (spacer: 5' TGGTTTGAAGGTGATGCAAAGGCTTTTAGC 3')	This paper	N/A
Plasmid: pU6-PspCas13b-gRNA-EPRS1-16727-14 (spacer: 5' GAAGGTGATGCAAAGGCTTTTAGCTCCCAT 3')	This paper	N/A
Plasmid: pU6-PspCas13b-gRNA-EPRS1-16726-14 (spacer: 5' GAATAATTGTCCTGTGTGACTTCATTTTAG 3')	This paper	N/A
Plasmid: pTRC-HisB-ERS	[8]	N/A
Plasmid: pTRC-HisB-PRS WT	[8]	N/A
Plasmid: pTRC-HisB-PRS P1482T	This paper	N/A
Plasmid: pMSCV-dCasRx-ALKBH5-PURO	Addgene	RRID:Addgene_175582
Plasmid: pXR003	Addgene	RRID:Addgene_109053
Plasmid: pXR003-NT (spacer: 5' CGTCTGGCCTTCCTGTAGCCAGCTTTCATC 3')	Sequence from [9]	N/A
Plasmid: pXR003-EPRS1-16728^{up10} (spacer: 5' AGAATGGAATCTGAACAATCTTTCCAGAAT 3')	This paper	N/A
Plasmid: pXR003-EPRS1-16728 (spacer: 5' TTTTGATCCAGTCCTCACAGTCAATTTCCC 3')	This paper	N/A
Plasmid: pXR003-EPRS1-16727 (spacer: 5' CATTTGGCTCCAGGCTGCAGTTCACAGAGT 3')	This paper	N/A
**Software and algorithms**		
MFOLD	[10]	http://www.unafold.org/mfold/applications/rna-folding-form.php
ImageJ	[11]	https://imagej.nih.gov/ij/
Fiji	[12]	https://fiji.sc/
Prism 9, Prism 10	GraphPad	https://www.graphpad.com/scientific-software/prism/
RNAstructure	[13]	https://rna.urmc.rochester.edu/RNAstructure.html

REAGENT or RESOURCE	SOURCE	IDENTIFIER
WebLogo	[14]	https://weblogo.berkeley.edu/logo.cgi
Clustal Omega	[15]	https://www.ebi.ac.uk/Tools/msa/clustalo/
ViennaRNA RNAfold	[16]	https://www.tbi.univie.ac.at/RNA/
GROMACS	[17]	https://www.gromacs.org
SWISS-MODEL	[18]	https://swissmodel.expasy.org
Pymol	[19]	https://pymol.org/2/
Python 3	[20]	https://dl.acm.org/doi/book/10.5555/1593511
PANTHER 17.0	[21]	https://www.pantherdb.org/
DAVID Bioinformatics Resources	[22]	https://david.ncifcrf.gov/summary.jsp

References

1. Jia, J., Arif, A., Ray, P.S. & Fox, P.L. WHEP domains direct noncanonical function of glutamyl-prolyl tRNA synthetase in translational control of gene expression. *Mol. Cell* **29**, 679-690 (2008).
2. Khan, D. *et al.* A viral pan-end RNA element and host complex define a SARS-CoV-2 regulon. *Nat. Commun.* **14**, 3385 (2023).
3. Tan, B. *et al.* RNA N^6-methyladenosine reader YTHDC1 is essential for TGF-beta-mediated metastasis of triple negative breast cancer. *Theranostics* **12**, 5727-5743 (2022).
4. Saito, Y. *et al.* YTHDC2 control of gametogenesis requires helicase activity but not m^6A binding. *Genes Dev.* **36**, 180-194 (2022).
5. Roundtree, I.A. *et al.* YTHDC1 mediates nuclear export of N^6-methyladenosine methylated mRNAs. *Elife* **6**, e31311 (2017).
6. Tang, J. *et al.* METTL16-mediated translation of CIDEA promotes non-alcoholic fatty liver disease progression via m^6A-dependent manner. *PeerJ* **10**, e14379 (2022).
7. Xiao, Y. *et al.* An elongation- and ligation-based qPCR amplification method for the radiolabeling-free detection of locus-specific N^6-methyladenosine modification. *Angew. Chem. Int. Ed. Engl.* **57**, 15995-16000 (2018).
8. Halawani, D. *et al.* Structural control of caspase-generated glutamyl-tRNA synthetase by appended noncatalytic WHEP domains. *J. Biol. Chem.* **293**, 8843-8860 (2018).
9. Xia, Z. *et al.* Epitranscriptomic editing of the RNA N6-methyladenosine modification by dCasRx conjugated methyltransferase and demethylase. *Nucleic Acids Res.* **49**, 7361-7374 (2021).
10. Zuker, M. Mfold web server for nucleic acid folding and hybridization prediction. *Nucleic Acids Res.* **31**, 3406-15 (2003).
11. Schneider, C.A., Rasband, W.S. & Eliceiri, K.W. NIH Image to ImageJ: 25 years of image analysis. *Nat. Methods* **9**, 671-5 (2012).
12. Schindelin, J. *et al.* Fiji: an open-source platform for biological-image analysis. *Nat. Methods* **9**, 676-82 (2012).
13. Reuter, J.S. & Mathews, D.H. RNAstructure: software for RNA secondary structure prediction and analysis. *BMC Bioinformatics* **11**, 129 (2010).
14. Crooks, G.E., Hon, G., Chandonia, J.M. & Brenner, S.E. WebLogo: a sequence logo generator. *Genome Res.* **14**, 1188-90 (2004).
15. Sievers, F. & Higgins, D.G. Clustal Omega for making accurate alignments of many protein sequences. *Protein Sci.* **27**, 135-145 (2018).
16. Lorenz, R. *et al.* ViennaRNA Package 2.0. *Algorithms Mol. Biol.* **6**, 26 (2011).
17. Pronk, S. *et al.* GROMACS 4.5: a high-throughput and highly parallel open source molecular simulation toolkit. *Bioinformatics* **29**, 845-54 (2013).
18. Schwede, T., Kopp, J., Guex, N. & Peitsch, M.C. SWISS-MODEL: An automated protein homology-modeling server. *Nucleic Acids Res.* **31**, 3381-5 (2003).
19. Seeliger, D. & de Groot, B.L. Ligand docking and binding site analysis with PyMOL and Autodock/Vina. *J. Comput. Aided Mol. Des.* **24**, 417-22 (2010).
20. Van Rossum, G. & Drake, F.L. *Python 3 Reference Manual.*, (CreateSpace, Scotts Valley, CA, 2009).
21. Thomas, P.D. *et al.* PANTHER: Making genome-scale phylogenetics accessible to all. *Protein Sci.* **31**, 8-22 (2022).
22. Huang da, W., Sherman, B.T. & Lempicki, R.A. Systematic and integrative analysis of large gene lists using DAVID bioinformatics resources. *Nat. Protoc.* **4**, 44-57 (2009).
