# Supplementary material for: Homozygous EPRS1 missense variant causing hypomyelinating leukodystrophy-15 alters variant-distal mRNA m6A site accessibility
Source: Nat Commun. 2024 May 20;15:4284. doi: 10.1038/s41467-024-48549-x (PMC11106242; doi:10.1038/s41467-024-48549-x)
Supplement: Supplementary file 3 — Description of Additional Supplementary Files [file 41467_2024_48549_MOESM3_ESM.pdf]

Description of Additional Supplementary Files

File Name: Supplementary Software 1

Description: The zip archive 'm6Ad-SNV-prediction.zip' contains:

1. a README.txt file containing a short description of the content of the archive and a brief instruction about how to run the pipeline;

an "input" folder with the BED file retrieved from the UCSC Genome Browser with whole gene regions;

2. an "output" folder with the results of the pipeline;

3. a "src" folder with the actual software pipeline.

Please note that:

A. the "input" folder contains just the BED file, but the pipeline automatically retrieves (i) the GENCODE annotation to extract the protein-coding regions, (ii) the ClinVar VCF file, (iii) the RMVar information about the m6A modifications, and (iv) the human genome version GRCh38. All these files will be automatically downloaded in the "input" folder;

B. the "src" folder contains a "pipeline.sh" file for an automatic execution of the pipeline, from the retrieval of the files listed above to the actual algorithm described in the manuscript. It may be easier for a reader to just execute this single file. The README.txt explains what the "pipeline.sh" does in particular, including a list of software requirements that a reader must install for its execution.
