# Supplementary material for: Homozygous EPRS1 missense variant causing hypomyelinating leukodystrophy-15 alters variant-distal mRNA m6A site accessibility
Source: Nat Commun. 2024 May 20;15:4284. doi: 10.1038/s41467-024-48549-x (PMC11106242; doi:10.1038/s41467-024-48549-x)
Supplement: Supplementary file 4 — Supplementary Software 1 [file 41467_2024_48549_MOESM4_ESM.zip › m6Ad-SNV-prediction/output/index/index.html]

Target sites


## Target index

Click on the IDs to inspect reference and alternate structures

| ClinVar ID | Chromosome | Start | End | Strand | Gene Symbol | Transcript ID | Minimum free energy (reference) | DRACH sites (reference) | Alternate position | Minimum free energy (alternate) | DRACH sites (alternate) | Structure state | deltaG | Synonymous | Free DRA sites (reference) | Free DRA sites (alternate) | Free RAC sites (reference) | Free RAC sites (alternate) | Free ACH sites (reference) | Free ACH sites (alternate) | Free DRAC sites (reference) | Free DRAC sites (alternate) | Free RACH sites (reference) | Free RACH sites (alternate) | Free DRACH sites (reference) | Free DRACH sites (alternate) |
| --- | --- | --- | --- | --- | --- | --- | --- | --- | --- | --- | --- | --- | --- | --- | --- | --- | --- | --- | --- | --- | --- | --- | --- | --- | --- | --- |
| 581116 | chr1 | 6467490 | 6468192 | - | PLEKHG5 | NM\_001265594.3 | -100.7 | 8 | 6468153 | -103.1 | 8 | paired-unpaired | -2.4 | no | 1 | 1 | 0 | 0 | 2 | 2 | 0 | 0 | 0 | 0 | 2 | 0 |
| 412459 | chr1 | 17018780 | 17022679 | - | SDHB | NM\_003000.3 | -58.6 | 7 | 17022612 | -56.8 | 7 | unpaired-paired | 1.8 | no | 0 | 0 | 1 | 0 | 2 | 2 | 0 | 0 | 0 | 0 | 0 | 0 |
| 623464 | chr1 | 150577263 | 150578864 | - | MCL1 | NM\_182763.3 | -71.4 | 6 | 150577397 | -71.7 | 6 | unpaired-paired | -0.3 | no | 0 | 0 | 0 | 0 | 2 | 1 | 1 | 0 | 0 | 0 | 0 | 1 |
| 446216 | chr1 | 234607036 | 234607286 | - | IRF2BP2 | NM\_001077397.1 | -70.4 | 5 | 234607249 | -69.0 | 5 | paired-unpaired | 1.4 | no | 1 | 1 | 0 | 0 | 0 | 0 | 1 | 1 | 0 | 0 | 0 | 1 |
| 446216 | chr1 | 234607036 | 234607286 | - | IRF2BP2 | NM\_182972.3 | -70.4 | 5 | 234607249 | -69.0 | 5 | paired-unpaired | 1.4 | no | 1 | 1 | 0 | 0 | 0 | 0 | 1 | 1 | 0 | 0 | 0 | 1 |
| 460345 | chr1 | 241497727 | 241500443 | - | FH | NM\_000143.4 | -39.4 | 9 | 241497890 | -39.4 | 9 | paired-unpaired | 0.0 | no | 2 | 1 | 0 | 0 | 0 | 0 | 1 | 1 | 0 | 0 | 2 | 2 |
| 336092 | chr2 | 43877676 | 43878013 | + | ABCG8 | NM\_001357321.2 | -78.0 | 8 | 43877786 | -77.7 | 8 | paired-unpaired | 0.3 | no | 0 | 1 | 0 | 0 | 2 | 0 | 0 | 0 | 0 | 0 | 0 | 2 |
| 458192 | chr2 | 47160675 | 47161843 | - | ENSG00000273269 | NM\_001305626.1 | -51.7 | 7 | 47161796 | -50.9 | 7 | unpaired-paired | 0.8 | yes | 0 | 0 | 0 | 0 | 0 | 0 | 0 | 0 | 1 | 1 | 0 | 0 |
| 489918 | chr2 | 47466689 | 47475155 | + | MSH2 | NM\_001406657.1 | -56.8 | 6 | 47466700 | -59.3 | 6 | paired-unpaired | -2.5 | no | 0 | 0 | 0 | 0 | 0 | 0 | 0 | 0 | 0 | 0 | 2 | 2 |
| 90999 | chr2 | 47480745 | 47482616 | + | MSH2 | NM\_001406648.1 | -46.3 | 6 | 47480795 | -46.5 | 6 | paired-unpaired | -0.2 | no | 1 | 3 | 0 | 0 | 0 | 1 | 0 | 0 | 0 | 0 | 2 | 1 |
| 91000 | chr2 | 47480745 | 47482616 | + | MSH2 | NM\_001406648.1 | -46.3 | 6 | 47480795 | -50.4 | 6 | paired-unpaired | -4.1 | no | 1 | 1 | 0 | 0 | 0 | 0 | 0 | 1 | 0 | 0 | 2 | 2 |
| 387732 | chr2 | 47480745 | 47482616 | + | MSH2 | NM\_001406648.1 | -46.3 | 6 | 47480796 | -45.2 | 6 | paired-unpaired | 1.1 | yes | 1 | 4 | 0 | 0 | 0 | 0 | 0 | 0 | 0 | 0 | 2 | 1 |
| 331658 | chr2 | 156325643 | 156325893 | - | NR4A2 | NM\_006186.4 | -64.4 | 8 | 156325882 | -66.9 | 8 | unpaired-paired | -2.5 | yes | 0 | 0 | 0 | 1 | 0 | 0 | 2 | 1 | 1 | 1 | 0 | 0 |
| 577709 | chr2 | 230169023 | 230172101 | - | SP110 | NM\_001378446.1 | -74.1 | 9 | 230169144 | -72.3 | 9 | unpaired-paired | 1.8 | no | 1 | 1 | 0 | 0 | 1 | 1 | 0 | 0 | 0 | 0 | 1 | 2 |
| 334890 | chr2 | 230169023 | 230170656 | - | SP110 | NM\_080424.4 | -66.7 | 9 | 230169144 | -64.0 | 9 | paired-unpaired | 2.7 | no | 0 | 0 | 0 | 0 | 0 | 0 | 0 | 0 | 1 | 0 | 2 | 1 |
| 334890 | chr2 | 230169023 | 230172101 | - | SP110 | NM\_001378446.1 | -74.1 | 9 | 230169144 | -71.3 | 9 | unpaired-paired | 2.8 | no | 1 | 1 | 0 | 0 | 1 | 1 | 0 | 0 | 0 | 0 | 1 | 1 |
| 2224 | chr3 | 10149815 | 10150065 | + | VHL | NM\_198156.3 | -68.3 | 7 | 10149819 | -68.5 | 7 | unpaired-paired | -0.2 | no | 0 | 2 | 0 | 0 | 1 | 0 | 1 | 0 | 0 | 1 | 1 | 1 |
| 2224 | chr3 | 10149815 | 10150065 | + | VHL | NM\_000551.4 | -68.3 | 7 | 10149819 | -68.5 | 7 | unpaired-paired | -0.2 | no | 0 | 2 | 0 | 0 | 1 | 0 | 1 | 0 | 0 | 1 | 1 | 1 |
| 12564 | chr3 | 24122783 | 24123033 | - | THRB | NM\_001252634.2 | -56.4 | 5 | 24122897 | -55.0 | 5 | paired-unpaired | 1.4 | no | 1 | 1 | 0 | 0 | 0 | 1 | 0 | 0 | 1 | 0 | 0 | 0 |
| 12564 | chr3 | 24122783 | 24123033 | - | THRB | NM\_001374827.1 | -56.4 | 5 | 24122897 | -55.0 | 5 | paired-unpaired | 1.4 | no | 1 | 1 | 0 | 0 | 0 | 1 | 0 | 0 | 1 | 0 | 0 | 0 |
| 100723 | chr4 | 1004096 | 1004493 | + | IDUA | NM\_000203.5 | -93.6 | 3 | 1004305 | -94.4 | 3 | paired-unpaired | -0.8 | no | 0 | 0 | 0 | 0 | 0 | 1 | 0 | 0 | 0 | 1 | 0 | 0 |
| 349452 | chr4 | 67740488 | 67744581 | - | GNRHR | NM\_001012763.2 | -50.2 | 8 | 67740694 | -48.9 | 8 | paired-unpaired | 1.3 | yes | 1 | 1 | 0 | 0 | 1 | 0 | 0 | 0 | 0 | 0 | 2 | 2 |
| 222976 | chr4 | 165341796 | 165342046 | + | MSMO1 | NM\_001017369.3 | -45.7 | 7 | 165341800 | -45.7 | 7 | paired-unpaired | 0.0 | no | 0 | 1 | 0 | 0 | 0 | 0 | 0 | 0 | 1 | 1 | 1 | 2 |
| 350898 | chr5 | 136062662 | 136063326 | + | TGFBI | NM\_000358.3 | -36.1 | 2 | 136063298 | -36.5 | 2 | paired-unpaired | -0.4 | no | 0 | 0 | 0 | 0 | 1 | 0 | 0 | 0 | 0 | 0 | 0 | 0 |
| 540173 | chr7 | 2537968 | 2538218 | - | BRAT1 | NM\_001350626.2 | -116.0 | 5 | 2538140 | -116.3 | 5 | unpaired-paired | -0.3 | no | 0 | 0 | 0 | 0 | 0 | 1 | 0 | 0 | 2 | 1 | 0 | 0 |
| 540173 | chr7 | 2537968 | 2538218 | - | BRAT1 | NM\_152743.4 | -116.0 | 5 | 2538140 | -116.3 | 5 | unpaired-paired | -0.3 | no | 0 | 0 | 0 | 0 | 0 | 1 | 0 | 0 | 2 | 1 | 0 | 0 |
| 480318 | chr7 | 5973298 | 5977593 | - | PMS2 | NM\_001406877.1 | -59.4 | 10 | 5973471 | -58.5 | 10 | paired-unpaired | 0.9 | yes | 0 | 0 | 1 | 0 | 0 | 0 | 0 | 1 | 1 | 2 | 1 | 1 |
| 480318 | chr7 | 5973298 | 5977593 | - | PMS2 | NM\_001406875.1 | -59.4 | 10 | 5973471 | -58.5 | 10 | paired-unpaired | 0.9 | yes | 0 | 0 | 1 | 0 | 0 | 0 | 0 | 1 | 1 | 2 | 1 | 1 |
| 480318 | chr7 | 5973298 | 5977593 | - | PMS2 | NM\_001406871.1 | -59.4 | 10 | 5973471 | -58.5 | 10 | paired-unpaired | 0.9 | yes | 0 | 0 | 1 | 0 | 0 | 0 | 0 | 1 | 1 | 2 | 1 | 1 |
| 359768 | chr7 | 22945488 | 22945738 | - | HYCC1 | NM\_032581.4 | -52.0 | 8 | 22945600 | -50.6 | 8 | paired-unpaired | 1.4 | no | 1 | 2 | 1 | 1 | 0 | 1 | 0 | 0 | 0 | 0 | 2 | 1 |
| 92588 | chr7 | 65960796 | 65961046 | - | GUSB | NM\_000181.4 | -58.1 | 7 | 65960907 | -57.5 | 7 | paired-unpaired | 0.6 | no | 0 | 1 | 0 | 0 | 1 | 1 | 1 | 1 | 0 | 0 | 1 | 1 |
| 469108 | chr7 | 66639082 | 66639332 | + | KCTD7 | NM\_153033.5 | -97.5 | 6 | 66639131 | -98.2 | 6 | unpaired-paired | -0.7 | no | 0 | 0 | 0 | 0 | 1 | 0 | 1 | 1 | 0 | 0 | 1 | 1 |
| 469108 | chr7 | 66639079 | 66640494 | + | KCTD7 | NM\_001167961.2 | -73.5 | 6 | 66639131 | -74.0 | 6 | paired-unpaired | -0.5 | no | 0 | 0 | 0 | 1 | 0 | 0 | 0 | 0 | 0 | 0 | 1 | 0 |
| 31194 | chr8 | 95247516 | 95247766 | - | CFAP418 | NM\_177965.4 | -59.7 | 8 | 95247712 | -57.8 | 8 | paired-unpaired | 1.9 | no | 0 | 1 | 0 | 0 | 0 | 0 | 0 | 0 | 1 | 1 | 2 | 1 |
| 31194 | chr8 | 95247516 | 95247766 | - | CFAP418 | NM\_001363260.1 | -59.7 | 8 | 95247712 | -57.8 | 8 | paired-unpaired | 1.9 | no | 0 | 1 | 0 | 0 | 0 | 0 | 0 | 0 | 1 | 1 | 2 | 1 |
| 254276 | chr9 | 27060620 | 27062836 | + | IFT74 | NM\_001099223.3 | -44.3 | 5 | 27062617 | -43.4 | 5 | paired-unpaired | 0.9 | no | 0 | 1 | 0 | 0 | 0 | 0 | 0 | 1 | 0 | 0 | 1 | 0 |
| 454834 | chr9 | 34517416 | 34520856 | + | DNAI1 | NM\_012144.4 | -78.2 | 6 | 34520701 | -80.1 | 7 | paired-unpaired | -1.9 | no | 1 | 2 | 0 | 0 | 1 | 1 | 0 | 0 | 1 | 0 | 0 | 0 |
| 459218 | chr9 | 105619978 | 105635186 | + | FKTN | NM\_001351498.2 | -50.1 | 8 | 105635056 | -48.6 | 8 | paired-unpaired | 1.5 | yes | 2 | 1 | 0 | 0 | 1 | 3 | 0 | 1 | 0 | 0 | 2 | 1 |
| 459218 | chr9 | 105635050 | 105640223 | + | FKTN | NM\_001198963.2 | -59.0 | 5 | 105635056 | -57.0 | 5 | unpaired-paired | 2.0 | yes | 0 | 0 | 0 | 0 | 0 | 0 | 0 | 0 | 1 | 1 | 1 | 0 |
| 364489 | chr9 | 105635050 | 105640223 | + | FKTN | NM\_001198963.2 | -59.0 | 5 | 105635066 | -57.0 | 5 | unpaired-paired | 2.0 | yes | 0 | 0 | 0 | 0 | 0 | 0 | 0 | 0 | 1 | 1 | 1 | 0 |
| 36137 | chr9 | 105619978 | 105635186 | + | FKTN | NM\_001351498.2 | -50.1 | 8 | 105635175 | -53.8 | 8 | paired-unpaired | -3.7 | no | 2 | 1 | 0 | 0 | 1 | 2 | 0 | 0 | 0 | 0 | 2 | 2 |
| 6465 | chr9 | 128540274 | 128541270 | + | GLE1 | NM\_001003722.2 | -52.2 | 5 | 128541124 | -52.7 | 5 | unpaired-paired | -0.5 | no | 0 | 0 | 0 | 0 | 1 | 0 | 0 | 0 | 0 | 0 | 0 | 2 |
| 698890 | chr10 | 18539574 | 18539824 | + | CACNB2 | NM\_201597.3 | -59.1 | 4 | 18539706 | -57.5 | 4 | paired-unpaired | 1.6 | yes | 0 | 0 | 0 | 0 | 1 | 1 | 0 | 0 | 0 | 0 | 1 | 1 |
| 136649 | chr10 | 18539574 | 18539824 | + | CACNB2 | NM\_201597.3 | -59.1 | 4 | 18539706 | -57.4 | 4 | paired-unpaired | 1.7 | no | 0 | 0 | 0 | 0 | 1 | 1 | 0 | 0 | 0 | 0 | 1 | 1 |
| 487634 | chr10 | 74114896 | 74118269 | + | VCL | NM\_014000.3 | -71.6 | 5 | 74118143 | -70.2 | 5 | paired-unpaired | 1.4 | no | 1 | 1 | 0 | 0 | 1 | 0 | 0 | 0 | 0 | 0 | 1 | 1 |
| 486801 | chr10 | 86923482 | 86923819 | + | BMPR1A | NM\_001406566.1 | -67.7 | 7 | 86923595 | -68.2 | 7 | unpaired-paired | -0.5 | no | 4 | 2 | 0 | 0 | 0 | 0 | 0 | 0 | 0 | 0 | 0 | 0 |
| 463828 | chr11 | 61438094 | 61446171 | + | ENSG00000256591 | NM\_017841.4 | -64.2 | 6 | 61446034 | -65.4 | 6 | paired-unpaired | -1.2 | no | 2 | 1 | 0 | 0 | 0 | 1 | 1 | 1 | 1 | 0 | 1 | 2 |
| 305312 | chr11 | 64804233 | 64804483 | - | MEN1 | NM\_001407150.1 | -83.2 | 6 | 64804245 | -82.3 | 6 | unpaired-paired | 0.9 | no | 0 | 1 | 1 | 0 | 1 | 2 | 0 | 0 | 0 | 1 | 0 | 0 |
| 305312 | chr11 | 64804233 | 64804483 | - | MEN1 | NM\_001407152.1 | -83.2 | 6 | 64804245 | -82.3 | 6 | unpaired-paired | 0.9 | no | 0 | 1 | 1 | 0 | 1 | 2 | 0 | 0 | 0 | 1 | 0 | 0 |
| 485069 | chr11 | 67490843 | 67491093 | + | AIP | NM\_003977.4 | -95.1 | 4 | 67490963 | -94.7 | 4 | paired-unpaired | 0.4 | no | 0 | 0 | 0 | 1 | 0 | 0 | 0 | 0 | 0 | 1 | 0 | 1 |
| 2888 | chr11 | 126424479 | 126424729 | - | KIRREL3 | NM\_032531.4 | -91.4 | 4 | 126424726 | -91.9 | 4 | paired-unpaired | -0.5 | no | 0 | 0 | 0 | 0 | 1 | 1 | 0 | 0 | 0 | 0 | 1 | 0 |
| 536845 | chr12 | 12718286 | 12721127 | + | CDKN1B | NM\_004064.5 | -51.6 | 3 | 12718869 | -48.2 | 3 | paired-unpaired | 3.4 | no | 0 | 0 | 0 | 0 | 0 | 0 | 0 | 0 | 0 | 0 | 2 | 2 |
| 404269 | chr12 | 12718286 | 12721127 | + | CDKN1B | NM\_004064.5 | -51.6 | 3 | 12718879 | -51.5 | 3 | paired-unpaired | 0.1 | no | 0 | 0 | 0 | 0 | 0 | 0 | 0 | 0 | 0 | 0 | 2 | 2 |
| 543382 | chr12 | 32640383 | 32640633 | + | FGD4 | NM\_001370298.3 | -73.4 | 8 | 32640431 | -71.2 | 8 | paired-unpaired | 2.2 | no | 0 | 1 | 0 | 0 | 1 | 3 | 0 | 0 | 0 | 0 | 1 | 0 |
| 1291049 | chr12 | 42459708 | 42459958 | - | PRICKLE1 | NM\_001144881.2 | -43.3 | 6 | 42459718 | -45.2 | 6 | paired-unpaired | -1.9 | no | 0 | 0 | 0 | 0 | 1 | 2 | 0 | 0 | 1 | 1 | 2 | 2 |
| 135603 | chr12 | 54283873 | 54284644 | + | HNRNPA1 | NM\_002136.4 | -58.0 | 6 | 54283944 | -62.3 | 6 | unpaired-paired | -4.3 | no | 3 | 2 | 0 | 0 | 0 | 0 | 0 | 0 | 0 | 0 | 2 | 1 |
| 310487 | chr12 | 76345712 | 76345962 | - | BBS10 | NM\_024685.4 | -42.5 | 12 | 76345726 | -43.2 | 12 | paired-unpaired | -0.7 | no | 1 | 0 | 0 | 0 | 0 | 1 | 1 | 1 | 1 | 1 | 2 | 2 |
| 409527 | chr13 | 32398620 | 32398870 | + | BRCA2 | NM\_001406722.1 | -42.7 | 7 | 32398637 | -40.1 | 7 | unpaired-paired | 2.6 | no | 0 | 0 | 0 | 0 | 0 | 0 | 0 | 0 | 1 | 1 | 3 | 3 |
| 527741 | chr13 | 76996006 | 77000543 | + | CLN5 | NM\_001366624.2 | -47.9 | 7 | 77000466 | -49.5 | 7 | paired-unpaired | -1.6 | no | 3 | 0 | 0 | 0 | 0 | 1 | 0 | 0 | 0 | 1 | 1 | 1 |
| 519710 | chr15 | 48410889 | 48411139 | - | FBN1 | NM\_001406716.1 | -32.9 | 10 | 48411003 | -32.3 | 10 | unpaired-paired | 0.6 | no | 1 | 0 | 0 | 0 | 0 | 1 | 2 | 0 | 0 | 1 | 4 | 4 |
| 213777 | chr15 | 67187483 | 67190636 | + | SMAD3 | NM\_001407012.1 | -81.4 | 5 | 67190486 | -81.0 | 5 | unpaired-paired | 0.4 | no | 0 | 0 | 0 | 0 | 1 | 0 | 0 | 0 | 0 | 0 | 1 | 1 |
| 1006099 | chr16 | 2088460 | 2088710 | + | TSC2 | NM\_001406686.1 | -83.3 | 4 | 2088493 | -85.2 | 4 | paired-unpaired | -1.9 | no | 0 | 0 | 0 | 0 | 0 | 0 | 0 | 1 | 0 | 0 | 0 | 3 |
| 1006099 | chr16 | 2088460 | 2088710 | + | TSC2 | NM\_001370405.1 | -83.3 | 4 | 2088493 | -85.2 | 4 | paired-unpaired | -1.9 | no | 0 | 0 | 0 | 0 | 0 | 0 | 0 | 1 | 0 | 0 | 0 | 3 |
| 486616 | chr16 | 2088460 | 2088710 | + | TSC2 | NM\_001406686.1 | -83.3 | 4 | 2088497 | -83.9 | 4 | paired-unpaired | -0.6 | no | 0 | 0 | 0 | 0 | 0 | 0 | 0 | 1 | 0 | 0 | 0 | 3 |
| 486616 | chr16 | 2088460 | 2088710 | + | TSC2 | NM\_001370405.1 | -83.3 | 4 | 2088497 | -83.9 | 4 | paired-unpaired | -0.6 | no | 0 | 0 | 0 | 0 | 0 | 0 | 0 | 1 | 0 | 0 | 0 | 3 |
| 655006 | chr16 | 2088460 | 2088710 | + | TSC2 | NM\_001406686.1 | -83.3 | 4 | 2088497 | -83.1 | 4 | paired-unpaired | 0.2 | no | 0 | 0 | 0 | 0 | 0 | 0 | 0 | 1 | 0 | 0 | 0 | 3 |
| 655006 | chr16 | 2088460 | 2088710 | + | TSC2 | NM\_001370405.1 | -83.3 | 4 | 2088497 | -83.1 | 4 | paired-unpaired | 0.2 | no | 0 | 0 | 0 | 0 | 0 | 0 | 0 | 1 | 0 | 0 | 0 | 3 |
| 536004 | chr16 | 2088460 | 2088710 | + | TSC2 | NM\_001406686.1 | -83.3 | 4 | 2088498 | -82.3 | 4 | paired-unpaired | 1.0 | no | 0 | 0 | 0 | 0 | 0 | 0 | 0 | 1 | 0 | 0 | 0 | 2 |
| 536004 | chr16 | 2088460 | 2088710 | + | TSC2 | NM\_001370405.1 | -83.3 | 4 | 2088498 | -82.3 | 4 | paired-unpaired | 1.0 | no | 0 | 0 | 0 | 0 | 0 | 0 | 0 | 1 | 0 | 0 | 0 | 2 |
| 49370 | chr16 | 2088460 | 2088710 | + | TSC2 | NM\_001406686.1 | -83.3 | 4 | 2088498 | -84.4 | 4 | paired-unpaired | -1.1 | no | 0 | 0 | 0 | 0 | 0 | 0 | 0 | 1 | 0 | 0 | 0 | 2 |
| 49370 | chr16 | 2088460 | 2088710 | + | TSC2 | NM\_001370405.1 | -83.3 | 4 | 2088498 | -84.4 | 4 | paired-unpaired | -1.1 | no | 0 | 0 | 0 | 0 | 0 | 0 | 0 | 1 | 0 | 0 | 0 | 2 |
| 535891 | chr16 | 2088460 | 2088710 | + | TSC2 | NM\_001406686.1 | -83.3 | 4 | 2088516 | -85.3 | 4 | paired-unpaired | -2.0 | no | 0 | 0 | 0 | 0 | 0 | 0 | 0 | 1 | 0 | 0 | 0 | 3 |
| 535891 | chr16 | 2088460 | 2088710 | + | TSC2 | NM\_001370405.1 | -83.3 | 4 | 2088516 | -85.3 | 4 | paired-unpaired | -2.0 | no | 0 | 0 | 0 | 0 | 0 | 0 | 0 | 1 | 0 | 0 | 0 | 3 |
| 468159 | chr16 | 2088460 | 2088710 | + | TSC2 | NM\_001406686.1 | -83.3 | 4 | 2088519 | -81.4 | 4 | paired-unpaired | 1.9 | no | 0 | 1 | 0 | 0 | 0 | 1 | 0 | 1 | 0 | 0 | 0 | 0 |
| 468159 | chr16 | 2088460 | 2088710 | + | TSC2 | NM\_001370405.1 | -83.3 | 4 | 2088519 | -81.4 | 4 | paired-unpaired | 1.9 | no | 0 | 1 | 0 | 0 | 0 | 1 | 0 | 1 | 0 | 0 | 0 | 0 |
| 1019286 | chr16 | 2088460 | 2088710 | + | TSC2 | NM\_001406686.1 | -83.3 | 4 | 2088539 | -83.3 | 4 | paired-unpaired | 0.0 | no | 0 | 1 | 0 | 0 | 0 | 1 | 0 | 1 | 0 | 0 | 0 | 0 |
| 1019286 | chr16 | 2088460 | 2088710 | + | TSC2 | NM\_001370405.1 | -83.3 | 4 | 2088539 | -83.3 | 4 | paired-unpaired | 0.0 | no | 0 | 1 | 0 | 0 | 0 | 1 | 0 | 1 | 0 | 0 | 0 | 0 |
| 65302 | chr16 | 2088460 | 2088710 | + | TSC2 | NM\_001406686.1 | -83.3 | 4 | 2088549 | -84.5 | 4 | paired-unpaired | -1.2 | no | 0 | 1 | 0 | 0 | 0 | 1 | 0 | 1 | 0 | 0 | 0 | 0 |
| 65302 | chr16 | 2088460 | 2088710 | + | TSC2 | NM\_001370405.1 | -83.3 | 4 | 2088549 | -84.5 | 4 | paired-unpaired | -1.2 | no | 0 | 1 | 0 | 0 | 0 | 1 | 0 | 1 | 0 | 0 | 0 | 0 |
| 318541 | chr16 | 23525026 | 23525276 | - | EARS2 | NM\_001308211.1 | -54.6 | 6 | 23525229 | -53.4 | 6 | unpaired-paired | 1.2 | yes | 0 | 0 | 0 | 0 | 1 | 1 | 0 | 0 | 1 | 0 | 1 | 1 |
| 126745 | chr16 | 23603358 | 23603608 | - | PALB2 | NM\_001407304.1 | -48.7 | 6 | 23603525 | -48.6 | 6 | paired-unpaired | 0.1 | yes | 1 | 2 | 0 | 0 | 1 | 1 | 0 | 0 | 0 | 0 | 0 | 0 |
| 126745 | chr16 | 23603358 | 23603608 | - | PALB2 | NM\_001407309.1 | -48.7 | 6 | 23603525 | -48.6 | 6 | paired-unpaired | 0.1 | yes | 1 | 2 | 0 | 0 | 1 | 1 | 0 | 0 | 0 | 0 | 0 | 0 |
| 234009 | chr16 | 23603358 | 23603608 | - | PALB2 | NM\_001407304.1 | -48.7 | 6 | 23603545 | -48.3 | 6 | paired-unpaired | 0.4 | no | 1 | 2 | 0 | 0 | 1 | 1 | 0 | 0 | 0 | 0 | 0 | 0 |
| 234009 | chr16 | 23603358 | 23603608 | - | PALB2 | NM\_001407309.1 | -48.7 | 6 | 23603545 | -48.3 | 6 | paired-unpaired | 0.4 | no | 1 | 2 | 0 | 0 | 1 | 1 | 0 | 0 | 0 | 0 | 0 | 0 |
| 378321 | chr16 | 23603358 | 23603608 | - | PALB2 | NM\_001407304.1 | -48.7 | 6 | 23603546 | -49.9 | 6 | paired-unpaired | -1.2 | yes | 1 | 2 | 0 | 0 | 1 | 1 | 0 | 0 | 0 | 0 | 0 | 0 |
| 378321 | chr16 | 23603358 | 23603608 | - | PALB2 | NM\_001407309.1 | -48.7 | 6 | 23603546 | -49.9 | 6 | paired-unpaired | -1.2 | yes | 1 | 2 | 0 | 0 | 1 | 1 | 0 | 0 | 0 | 0 | 0 | 0 |
| 182777 | chr16 | 23603358 | 23603608 | - | PALB2 | NM\_001407304.1 | -48.7 | 6 | 23603546 | -48.7 | 6 | paired-unpaired | 0.0 | no | 1 | 2 | 0 | 0 | 1 | 1 | 0 | 0 | 0 | 0 | 0 | 0 |
| 182777 | chr16 | 23603358 | 23603608 | - | PALB2 | NM\_001407309.1 | -48.7 | 6 | 23603546 | -48.7 | 6 | paired-unpaired | 0.0 | no | 1 | 2 | 0 | 0 | 1 | 1 | 0 | 0 | 0 | 0 | 0 | 0 |
| 182776 | chr16 | 23603358 | 23603608 | - | PALB2 | NM\_001407304.1 | -48.7 | 6 | 23603568 | -48.8 | 6 | paired-unpaired | -0.1 | no | 1 | 1 | 0 | 0 | 1 | 1 | 0 | 0 | 0 | 1 | 0 | 0 |
| 182776 | chr16 | 23603358 | 23603608 | - | PALB2 | NM\_001407309.1 | -48.7 | 6 | 23603568 | -48.8 | 6 | paired-unpaired | -0.1 | no | 1 | 1 | 0 | 0 | 1 | 1 | 0 | 0 | 0 | 1 | 0 | 0 |
| 141936 | chr16 | 23603358 | 23603608 | - | PALB2 | NM\_001407304.1 | -48.7 | 6 | 23603571 | -51.1 | 6 | paired-unpaired | -2.4 | no | 1 | 1 | 0 | 0 | 1 | 1 | 0 | 0 | 0 | 1 | 0 | 0 |
| 141936 | chr16 | 23603358 | 23603608 | - | PALB2 | NM\_001407309.1 | -48.7 | 6 | 23603571 | -51.1 | 6 | paired-unpaired | -2.4 | no | 1 | 1 | 0 | 0 | 1 | 1 | 0 | 0 | 0 | 1 | 0 | 0 |
| 484208 | chr16 | 23603358 | 23603608 | - | PALB2 | NM\_001407304.1 | -48.7 | 6 | 23603581 | -49.8 | 6 | paired-unpaired | -1.1 | no | 1 | 1 | 0 | 0 | 1 | 2 | 0 | 0 | 0 | 1 | 0 | 0 |
| 484208 | chr16 | 23603358 | 23603608 | - | PALB2 | NM\_001407309.1 | -48.7 | 6 | 23603581 | -49.8 | 6 | paired-unpaired | -1.1 | no | 1 | 1 | 0 | 0 | 1 | 2 | 0 | 0 | 0 | 1 | 0 | 0 |
| 628361 | chr16 | 23603358 | 23603608 | - | PALB2 | NM\_001407304.1 | -48.7 | 6 | 23603589 | -50.9 | 6 | paired-unpaired | -2.2 | no | 1 | 2 | 0 | 0 | 1 | 1 | 0 | 0 | 0 | 0 | 0 | 0 |
| 628361 | chr16 | 23603358 | 23603608 | - | PALB2 | NM\_001407309.1 | -48.7 | 6 | 23603589 | -50.9 | 6 | paired-unpaired | -2.2 | no | 1 | 2 | 0 | 0 | 1 | 1 | 0 | 0 | 0 | 0 | 0 | 0 |
| 126741 | chr16 | 23603358 | 23603608 | - | PALB2 | NM\_001407304.1 | -48.7 | 6 | 23603592 | -50.2 | 6 | paired-unpaired | -1.5 | no | 1 | 2 | 0 | 0 | 1 | 1 | 0 | 0 | 0 | 0 | 0 | 0 |
| 126741 | chr16 | 23603358 | 23603608 | - | PALB2 | NM\_001407309.1 | -48.7 | 6 | 23603592 | -50.2 | 6 | paired-unpaired | -1.5 | no | 1 | 2 | 0 | 0 | 1 | 1 | 0 | 0 | 0 | 0 | 0 | 0 |
| 530122 | chr16 | 23603554 | 23614091 | - | PALB2 | NM\_001407310.1 | -37.4 | 4 | 23603605 | -36.3 | 4 | unpaired-paired | 1.1 | no | 0 | 0 | 1 | 0 | 1 | 1 | 1 | 0 | 0 | 0 | 0 | 0 |
| 530116 | chr16 | 23603358 | 23603608 | - | PALB2 | NM\_001407304.1 | -48.7 | 6 | 23603605 | -47.0 | 6 | paired-unpaired | 1.7 | no | 1 | 3 | 0 | 0 | 1 | 0 | 0 | 0 | 0 | 0 | 0 | 0 |
| 530116 | chr16 | 23603554 | 23614091 | - | PALB2 | NM\_001407310.1 | -37.4 | 4 | 23603605 | -38.0 | 4 | unpaired-paired | -0.6 | no | 0 | 0 | 1 | 0 | 1 | 1 | 1 | 0 | 0 | 0 | 0 | 0 |
| 530116 | chr16 | 23603358 | 23603608 | - | PALB2 | NM\_001407309.1 | -48.7 | 6 | 23603605 | -47.0 | 6 | paired-unpaired | 1.7 | no | 1 | 3 | 0 | 0 | 1 | 0 | 0 | 0 | 0 | 0 | 0 | 0 |
| 460993 | chr16 | 23603554 | 23614091 | - | PALB2 | NM\_001407310.1 | -37.4 | 4 | 23603626 | -38.0 | 4 | unpaired-paired | -0.6 | yes | 0 | 0 | 1 | 0 | 1 | 1 | 1 | 0 | 0 | 0 | 0 | 0 |
| 229790 | chr16 | 23603554 | 23614091 | - | PALB2 | NM\_001407310.1 | -37.4 | 4 | 23603628 | -37.3 | 4 | unpaired-paired | 0.1 | no | 0 | 0 | 1 | 0 | 1 | 1 | 1 | 0 | 0 | 0 | 0 | 0 |
| 322845 | chr17 | 3659959 | 3660469 | + | CTNS | NM\_001374496.1 | -85.2 | 6 | 3660273 | -87.0 | 6 | paired-unpaired | -1.8 | no | 0 | 0 | 1 | 0 | 1 | 0 | 0 | 0 | 0 | 0 | 0 | 1 |
| 322846 | chr17 | 3660318 | 3660834 | + | CTNS | NM\_001031681.3 | -104.7 | 5 | 3660348 | -104.1 | 5 | unpaired-paired | 0.6 | yes | 0 | 0 | 0 | 0 | 0 | 1 | 1 | 0 | 1 | 1 | 1 | 1 |
| 630954 | chr17 | 35100852 | 35101266 | - | ENSG00000267618 | NM\_001142571.2 | -93.8 | 8 | 35101029 | -94.0 | 8 | unpaired-paired | -0.2 | no | 1 | 2 | 1 | 1 | 2 | 2 | 0 | 0 | 0 | 0 | 0 | 0 |
| 141707 | chr17 | 35100852 | 35101266 | - | ENSG00000267618 | NM\_001142571.2 | -93.8 | 8 | 35101029 | -93.4 | 8 | paired-unpaired | 0.4 | no | 1 | 2 | 1 | 0 | 2 | 2 | 0 | 0 | 0 | 1 | 0 | 0 |
| 492457 | chr18 | 51078317 | 51078567 | + | SMAD4 | NM\_001407041.1 | -50.1 | 7 | 51078468 | -50.4 | 7 | unpaired-paired | -0.3 | no | 1 | 1 | 0 | 0 | 0 | 0 | 0 | 0 | 3 | 3 | 2 | 1 |
| 630655 | chr18 | 51078317 | 51078567 | + | SMAD4 | NM\_001407041.1 | -50.1 | 7 | 51078468 | -52.0 | 7 | unpaired-paired | -1.9 | no | 1 | 1 | 0 | 0 | 0 | 0 | 0 | 0 | 3 | 3 | 2 | 1 |
| 413352 | chr21 | 43053779 | 43056848 | - | CBS | NM\_001321072.1 | -95.6 | 6 | 43053961 | -92.5 | 6 | paired-unpaired | 3.1 | yes | 0 | 1 | 0 | 0 | 1 | 0 | 0 | 0 | 1 | 0 | 1 | 0 |
| 413352 | chr21 | 43053565 | 43056848 | - | CBS | NM\_001178009.3 | -87.3 | 6 | 43053961 | -84.2 | 6 | paired-unpaired | 3.1 | yes | 1 | 1 | 0 | 0 | 0 | 0 | 0 | 1 | 0 | 0 | 0 | 0 |
| 476102 | chr21 | 43416641 | 43416891 | - | SIK1 | NM\_173354.5 | -91.3 | 7 | 43416890 | -88.6 | 7 | paired-unpaired | 2.7 | no | 1 | 1 | 0 | 0 | 1 | 2 | 0 | 0 | 0 | 0 | 1 | 0 |
| 264270 | chr22 | 19895032 | 19895488 | - | TXNRD2 | NM\_001352303.2 | -80.9 | 7 | 19895439 | -81.6 | 7 | paired-unpaired | -0.7 | no | 2 | 1 | 0 | 0 | 1 | 2 | 0 | 0 | 0 | 0 | 1 | 0 |
| 11321 | chrX | 101346398 | 101346648 | - | TIMM8A | NM\_004085.4 | -73.2 | 5 | 101346595 | -76.0 | 5 | unpaired-paired | -2.8 | no | 0 | 0 | 0 | 0 | 0 | 0 | 0 | 0 | 1 | 2 | 1 | 1 |
| 11559 | chrX | 136209376 | 136210054 | + | FHL1 | NM\_001159702.3 | -67.1 | 2 | 136209392 | -66.2 | 2 | unpaired-paired | 0.9 | no | 0 | 0 | 0 | 0 | 0 | 0 | 0 | 0 | 0 | 0 | 1 | 0 |
| 580161 | chrX | 154776243 | 154776967 | + | DKC1 | NM\_001142463.3 | -45.8 | 6 | 154776855 | -44.84 | 6 | unpaired-paired | 0.96 | no | 2 | 2 | 0 | 0 | 1 | 0 | 0 | 0 | 0 | 0 | 1 | 1 |
| ClinVar ID | Chromosome | Start | End | Strand | Gene Symbol | Transcript ID | Minimum free energy (reference) | DRACH sites (reference) | Alternate position | Minimum free energy (alternate) | DRACH sites (alternate) | Structure state | deltaG | Synonymous | Free DRA sites (reference) | Free DRA sites (alternate) | Free RAC sites (reference) | Free RAC sites (alternate) | Free ACH sites (reference) | Free ACH sites (alternate) | Free DRAC sites (reference) | Free DRAC sites (alternate) | Free RACH sites (reference) | Free RACH sites (alternate) | Free DRACH sites (reference) | Free DRACH sites (alternate) |
| --- | --- | --- | --- | --- | --- | --- | --- | --- | --- | --- | --- | --- | --- | --- | --- | --- | --- | --- | --- | --- | --- | --- | --- | --- | --- | --- |
