# Supplementary material for: Homozygous EPRS1 missense variant causing hypomyelinating leukodystrophy-15 alters variant-distal mRNA m6A site accessibility
Source: Nat Commun. 2024 May 20;15:4284. doi: 10.1038/s41467-024-48549-x (PMC11106242; doi:10.1038/s41467-024-48549-x)
Supplement: Supplementary file 4 — Supplementary Software 1 [file 41467_2024_48549_MOESM4_ESM.zip › m6Ad-SNV-prediction/output/index/data/349452_NM_001012763.2.html]

RNAPlot - 349452 - NM\_001012763.2


## Target ID: 349452\_NM\_001012763.2

https://www.ncbi.nlm.nih.gov/clinvar/variation/349452/

https://www.ncbi.nlm.nih.gov/nuccore/NM\_001012763.2

#### Reference

|  |  |
| --- | --- |
| Sequence | CAGGACCCCCACGGACTACAACTGAATCAGTCCAAGAACAATATACCAAGAGCACGGCTGAAGACTCTAAAAATGACGGTTGCATTTGCCACTTCATTTACTGTCTGCTGGACTCCCTACTATGTCCTAGGAATTTGGTATTGGTTTGATCCTGAAATGTTAAACAGGTTGTCAGACCCAGTAAATCACTTCTTCTTTCTCTTTGCCTTTTTAAACCCATGCTTTGATCCACTTATCTATGGATATTTTT |
| Base | T |
| Structure | .(((........(((((...........))))).........((((((((((..((((...((((.....((((((.(((.((....)).)))))))))...))))))))..)))((((........))))...))))))).((((((((((((..........))))..))))))))...........................)))..................(((((........)))))...... |
| Colors | 3-7:green 13-17:green 36-40:green 62-66:green 110-114:green 162-166:green 174-178:green 213-217:green 44:orange |

Show reference structure

#### Alternate

|  |  |
| --- | --- |
| Sequence | CAGGACCCCCACGGACTACAACTGAATCAGTCCAAGAACAATACACCAAGAGCACGGCTGAAGACTCTAAAAATGACGGTTGCATTTGCCACTTCATTTACTGTCTGCTGGACTCCCTACTATGTCCTAGGAATTTGGTATTGGTTTGATCCTGAAATGTTAAACAGGTTGTCAGACCCAGTAAATCACTTCTTCTTTCTCTTTGCCTTTTTAAACCCATGCTTTGATCCACTTATCTATGGATATTTTT |
| Base | C |
| Structure | .(((((......(((((...........)))))................(((..((((...((((.....((((((.(((.((....)).)))))))))...))))))))..)))........)))))(((((..(((((((((((((((((((..........))))..)))))).)))))..)))).)))))................................(((((........)))))...... |
| Colors | 3-7:green 13-17:green 36-40:green 62-66:green 110-114:green 162-166:green 174-178:green 213-217:green 44:orange |

Show alternate structure
