# Supplementary material for: Homozygous EPRS1 missense variant causing hypomyelinating leukodystrophy-15 alters variant-distal mRNA m6A site accessibility
Source: Nat Commun. 2024 May 20;15:4284. doi: 10.1038/s41467-024-48549-x (PMC11106242; doi:10.1038/s41467-024-48549-x)
Supplement: Supplementary file 4 — Supplementary Software 1 [file 41467_2024_48549_MOESM4_ESM.zip › m6Ad-SNV-prediction/output/index/data/254276_NM_001099223.3.html]

RNAPlot - 254276 - NM\_001099223.3


## Target ID: 254276\_NM\_001099223.3

https://www.ncbi.nlm.nih.gov/clinvar/variation/254276/

https://www.ncbi.nlm.nih.gov/nuccore/NM\_001099223.3

#### Reference

|  |  |
| --- | --- |
| Sequence | CCTTGAGCAAAATAATTTTGCGATGAAAGAAGTCATAGCAACCAAGAGTCAAGAGAGTGATTACCAGCCAATTAAGAAAAATGTGACCAAGCAGATTGCAGAGTACAATAAAACCATCGTGGATGCTTTACATAGCACCAGCGGAAACTGAGTTTAAGTCCACTGAAAGTCTCTAAGGAAGTATCCTCTTGCTGCTAAACTTGGTACAAGTTGACTACCAAAAAAAAAAAAAGCTTACTTTTGGAGTTTA |
| Base | G |
| Structure | .....(((((....((((((((((.......((((((.........(((((......)))))...................)))))).......))))))))))................(((((((((....((.(((((.(((..((.......))))).)))...)))).....)))))))))..)))))....((((((...))))))((((.((((((...............)))))))))).. |
| Colors | 84-88:green 111-115:green 145-149:green 197-201:green 212-216:green 32:orange |

Show reference structure

#### Alternate

|  |  |
| --- | --- |
| Sequence | CCTTGAGCAAAATAATTTTGCGATGAAAGAATTCATAGCAACCAAGAGTCAAGAGAGTGATTACCAGCCAATTAAGAAAAATGTGACCAAGCAGATTGCAGAGTACAATAAAACCATCGTGGATGCTTTACATAGCACCAGCGGAAACTGAGTTTAAGTCCACTGAAAGTCTCTAAGGAAGTATCCTCTTGCTGCTAAACTTGGTACAAGTTGACTACCAAAAAAAAAAAAAGCTTACTTTTGGAGTTTA |
| Base | T |
| Structure | .((((.((((((...)))))).(((((....)))))....(((((((((...(((((.(((.((...((.....(((.....(((((...(((...)))...........((((((((((((.((((......))))))).)))....)).))))..)).)))......)))....))..))))))))))...)))...)))))).))))..((((.((((((...............)))))))))).. |
| Colors | 84-88:green 111-115:green 145-149:green 197-201:green 212-216:green 32:orange |

Show alternate structure
