# Supplementary material for: Homozygous EPRS1 missense variant causing hypomyelinating leukodystrophy-15 alters variant-distal mRNA m6A site accessibility
Source: Nat Commun. 2024 May 20;15:4284. doi: 10.1038/s41467-024-48549-x (PMC11106242; doi:10.1038/s41467-024-48549-x)
Supplement: Supplementary file 4 — Supplementary Software 1 [file 41467_2024_48549_MOESM4_ESM.zip › m6Ad-SNV-prediction/output/index/data/65302_NM_001406686.1.html]

RNAPlot - 65302 - NM\_001406686.1


## Target ID: 65302\_NM\_001406686.1

https://www.ncbi.nlm.nih.gov/clinvar/variation/65302/

https://www.ncbi.nlm.nih.gov/nuccore/NM\_001406686.1

#### Reference

|  |  |
| --- | --- |
| Sequence | CGCCTACTCCAACCCCAGCCTACCTCTGGTGCACCCTCCGTCCCATAGCAAAGCCCCTGCACAGACTCCAGCCGAGCCCACACCTGGCTATGAGGTGGGCCAGCGGAAGCGCCTCATCTCCTCGGTGGAGGACTTCACCGAGTTTGTGTGAGGCCGGGGCCCTCCCTCCTGCACTGGCCTTGGACGGTATTGCCTGTCAGTGAAATAAATAAAGTCCTGACCCCAGTGCACAGACATAGAGGCACAGATT |
| Base | A |
| Structure | .................((((...((((.(((((...((((((...............((((((((((.....(((((..((((.((..((((((((..((...))...))))))))..))..))))..)).)))....))))))))))((((((((.((..........)).))))))))))))))........(((((.((...........)))))))....))))))))).....))))....... |
| Colors | 63-67:green 130-134:green 218-222:green 232-236:green 90:orange |

Show reference structure

#### Alternate

|  |  |
| --- | --- |
| Sequence | CGCCTACTCCAACCCCAGCCTACCTCTGGTGCACCCTCCGTCCCATAGCAAAGCCCCTGCACAGACTCCAGCCGAGCCCACACCTGGCTTTGAGGTGGGCCAGCGGAAGCGCCTCATCTCCTCGGTGGAGGACTTCACCGAGTTTGTGTGAGGCCGGGGCCCTCCCTCCTGCACTGGCCTTGGACGGTATTGCCTGTCAGTGAAATAAATAAAGTCCTGACCCCAGTGCACAGACATAGAGGCACAGATT |
| Base | T |
| Structure | .................((((...((((.(((((..((((((((((..(((((((..........(((.....))).........)))))))..)))))...)))))...(((((((...(((((((((....))))))))).....)))))))((((((...........(((((((...((.((....)))).)))))))...........))))))......))))))))).....))))....... |
| Colors | 63-67:green 130-134:green 218-222:green 232-236:green 90:orange |

Show alternate structure
