# Supplementary material for: Homozygous EPRS1 missense variant causing hypomyelinating leukodystrophy-15 alters variant-distal mRNA m6A site accessibility
Source: Nat Commun. 2024 May 20;15:4284. doi: 10.1038/s41467-024-48549-x (PMC11106242; doi:10.1038/s41467-024-48549-x)
Supplement: Supplementary file 4 — Supplementary Software 1 [file 41467_2024_48549_MOESM4_ESM.zip › m6Ad-SNV-prediction/output/index/data/359768_NM_032581.4.html]

RNAPlot - 359768 - NM\_032581.4


## Target ID: 359768\_NM\_032581.4

https://www.ncbi.nlm.nih.gov/clinvar/variation/359768/

https://www.ncbi.nlm.nih.gov/nuccore/NM\_032581.4

#### Reference

|  |  |
| --- | --- |
| Sequence | GGGCTGGGACAGATGCCAATAGGTTTTCCGCTTGTAGTCTCCAAGAAGAAAAGCTTATTTACGTTTCAGAAAGAACTGAACTTCCAATGAAGCATCAATCAGGTCAGCAGAGACCTCCTAGTATTAGCATTACTCTGTCCACAGATTAATTAGTAACATATTTTTCTCCCATAACCTAGTGAACCTGGAAATACAACTTTGCTTCTTTATGAAAGTACCCTGGGTCTTTCATCCGTATTCCTGACAGGAG |
| Base | T |
| Structure | ((((((((((((((((.(((((((((((..((((.......))))..)).))))))))).....(((((......)))))((((....))))........(((((......)))))..........)))....))))))).))).......................))).......((((((..((......))..)))))).....(((((((.(((...))))))))))......((((...)))). |
| Colors | 7-11:green 73-77:green 78-82:green 111-115:green 154-158:green 172-176:green 181-185:green 242-246:green 138:orange |

Show reference structure

#### Alternate

|  |  |
| --- | --- |
| Sequence | GGGCTGGGACAGATGCCAATAGGTTTTCCGCTTGTAGTCTCCAAGAAGAAAAGCTTATTTACGTTTCAGAAAGAACTGAACTTCCAATGAAGCATCAATCAGGTCAGCAGAGACCTCCTAGTATTAGCATTACTCTGCCCACAGATTAATTAGTAACATATTTTTCTCCCATAACCTAGTGAACCTGGAAATACAACTTTGCTTCTTTATGAAAGTACCCTGGGTCTTTCATCCGTATTCCTGACAGGAG |
| Base | C |
| Structure | (((.(((((..((((..(((((((((((..((((.......))))..)).)))))))))..))))((((......)))).((((....))))....((((..((..((((((.....(((....))).....))))))..)).))))...................)))))...)))((((((..((......))..)))))).....(((((((.(((...))))))))))......((((...)))). |
| Colors | 7-11:green 73-77:green 78-82:green 111-115:green 154-158:green 172-176:green 181-185:green 242-246:green 138:orange |

Show alternate structure
