# Supplementary material for: Homozygous EPRS1 missense variant causing hypomyelinating leukodystrophy-15 alters variant-distal mRNA m6A site accessibility
Source: Nat Commun. 2024 May 20;15:4284. doi: 10.1038/s41467-024-48549-x (PMC11106242; doi:10.1038/s41467-024-48549-x)
Supplement: Supplementary file 4 — Supplementary Software 1 [file 41467_2024_48549_MOESM4_ESM.zip › m6Ad-SNV-prediction/output/index/data/318541_NM_001308211.1.html]

RNAPlot - 318541 - NM\_001308211.1


## Target ID: 318541\_NM\_001308211.1

https://www.ncbi.nlm.nih.gov/clinvar/variation/318541/

https://www.ncbi.nlm.nih.gov/nuccore/NM\_001308211.1

#### Reference

|  |  |
| --- | --- |
| Sequence | AACTCCTTCGGATGGCCCTCAGTGGACAGCAGGTGAGGCAGGGACACGGGTTGGATTGTTCCCTGGAGCCCCTCATTGATCCTTTGAACTTACATTTCCTGGCAGGCACTGAGCTCAATATTGAGTATACAAAGGTGAATGAAACATGATCACTCCCCTCATTTAGTCAATCAACAGACACTATTGAGTACCTACTATGTGCAAGCATTGTGTTAGGTACAGTGAATACAATATAGAATTAAACACACAT |
| Base | C |
| Structure | ..((((...(((((....((((.(((.....(((((((((((((.((((......)))))))))).....)))))))..))).)))).....)))))...)).)).(((((.(((((....))))).......((.((.(((.......))).))))((((....(((........))).....))))(((((((.((((....)))).))...)))))))))).......................... |
| Colors | 24-28:green 42-46:green 86-90:green 142-146:green 176-180:green 241-245:green 47:orange |

Show reference structure

#### Alternate

|  |  |
| --- | --- |
| Sequence | AACTCCTTCGGATGGCCCTCAGTGGACAGCAGGTGAGGCAGGGACATGGGTTGGATTGTTCCCTGGAGCCCCTCATTGATCCTTTGAACTTACATTTCCTGGCAGGCACTGAGCTCAATATTGAGTATACAAAGGTGAATGAAACATGATCACTCCCCTCATTTAGTCAATCAACAGACACTATTGAGTACCTACTATGTGCAAGCATTGTGTTAGGTACAGTGAATACAATATAGAATTAAACACACAT |
| Base | T |
| Structure | ...(((...))).((..((((((((.....((((((((..(((((((((((((((....)))....))))..(((((...((((((.(((((..........(((...)))..........)))))...))))))..)))))..)))).)).))..)))))))).(((........))).))))))))..))..((((((.....(((((((.....)))))))......)))))).............. |
| Colors | 24-28:green 42-46:green 86-90:green 142-146:green 176-180:green 241-245:green 47:orange |

Show alternate structure
