# Supplementary material for: Homozygous EPRS1 missense variant causing hypomyelinating leukodystrophy-15 alters variant-distal mRNA m6A site accessibility
Source: Nat Commun. 2024 May 20;15:4284. doi: 10.1038/s41467-024-48549-x (PMC11106242; doi:10.1038/s41467-024-48549-x)
Supplement: Supplementary file 4 — Supplementary Software 1 [file 41467_2024_48549_MOESM4_ESM.zip › m6Ad-SNV-prediction/output/index/data/630655_NM_001407041.1.html]

RNAPlot - 630655 - NM\_001407041.1


## Target ID: 630655\_NM\_001407041.1

https://www.ncbi.nlm.nih.gov/clinvar/variation/630655/

https://www.ncbi.nlm.nih.gov/nuccore/NM\_001407041.1

#### Reference

|  |  |
| --- | --- |
| Sequence | GAGTTTTGTGAAAGGCTGGGGACCGGATTACCCAAGACAGAGCATCAAAGAAACACCTTGCTGGATTGAAATTCACTTACACCGGGCCCTCCAGCTCCTAGACGAAGTACTTCATACCATGCCGATTGCAGACCCACAACCTTTAGACTGAGGTCTTTTACCGTTGGGGCCCTTAACCTTATCAGGATGGTGGACTACAAAATACAATCCTGTTTATAATCTGAAGATATATTTCACTTTTGTTCTGCTT |
| Base | G |
| Structure | .((((((....))))))(((..........)))(((.(((((((..((((.........((.((((((...........((((((((((..((((........(((....)))......(((.....)))........(((((......)))))........))))))))))....(((....)))..))))............)))))).))........(((((.....))))))))))))))))))) |
| Colors | 20-24:green 35-39:green 51-55:green 130-134:green 145-149:green 174-178:green 192-196:green 152:orange |

Show reference structure

#### Alternate

|  |  |
| --- | --- |
| Sequence | GAGTTTTGTGAAAGGCTGGGGACCGGATTACCCAAGACAGAGCATCAAAGAAACACCTTGCTGGATTGAAATTCACTTACACCGGGCCCTCCAGCTCCTAGACGAAGTACTTCATACCATGCCGATTGCAGACCCACAACCTTTAGACTGACGTCTTTTACCGTTGGGGCCCTTAACCTTATCAGGATGGTGGACTACAAAATACAATCCTGTTTATAATCTGAAGATATATTTCACTTTTGTTCTGCTT |
| Base | C |
| Structure | .((((((....))))))(((..........)))(((.(((((((..((((.........((.((((((...........((((((((((..((((....(((((.(((.((........(((.....)))..............)))))..)))))......))))))))))....(((....)))..))))............)))))).))........(((((.....))))))))))))))))))) |
| Colors | 20-24:green 35-39:green 51-55:green 130-134:green 145-149:green 174-178:green 192-196:green 152:orange |

Show alternate structure
