# Supplementary material for: Homozygous EPRS1 missense variant causing hypomyelinating leukodystrophy-15 alters variant-distal mRNA m6A site accessibility
Source: Nat Commun. 2024 May 20;15:4284. doi: 10.1038/s41467-024-48549-x (PMC11106242; doi:10.1038/s41467-024-48549-x)
Supplement: Supplementary file 4 — Supplementary Software 1 [file 41467_2024_48549_MOESM4_ESM.zip › m6Ad-SNV-prediction/output/index/data/229790_NM_001407310.1.html]

RNAPlot - 229790 - NM\_001407310.1


## Target ID: 229790\_NM\_001407310.1

https://www.ncbi.nlm.nih.gov/clinvar/variation/229790/

https://www.ncbi.nlm.nih.gov/nuccore/NM\_001407310.1

#### Reference

|  |  |
| --- | --- |
| Sequence | AATTTAAAAACTGGTCAACTCCTGAAAAAGATGCACATTGATGATTCTTACCAAGCTTCAGTCTGTCACAAAGCCTATTCTGAAATGGTTCCTGGAAGGTGACGTGAAAGATCACTGTGCAGCAGCAATCTTGACTTCTGGAACAATTGCCATTTGGGACTTACTTCTCGGTCAGTGTACTGCCCTCCTCCCACCTGTCTCTG |
| Base | T |
| Structure | ...........((((....(((.(((.((((((((((.((.(((..(((...)))..)))((((.((((...((((.(((.(((....)))..)))))))...)))).)))))).)))))).......))))...))).)))......))))...(((((.........((.(((....))).))...........))))).. |
| Colors | 8-12:green 132-136:green 141-145:green 157-161:green 129:orange |

Show reference structure

#### Alternate

|  |  |
| --- | --- |
| Sequence | AATTTAAAAACTGGTCAACTCCTGAAAAAGATGCACATTGATGATTCTTACCAAGCTTCAGTCTGTCACAAAGCCTATTCTGAAATGGTTCCTGGAAGGTGACGTGAAAGATCACTGTGCAGCAGCAACCTTGACTTCTGGAACAATTGCCATTTGGGACTTACTTCTCGGTCAGTGTACTGCCCTCCTCCCACCTGTCTCTG |
| Base | C |
| Structure | .........((.(((......((.....)).((((((.((.(((..(((...)))..)))((((.((((...((((.(((.(((....)))..)))))))...)))).)))))).))))))((((..((.((((((...((((......((.....))......)))).)))))).)).)))).........))).))..... |
| Colors | 8-12:green 132-136:green 141-145:green 157-161:green 129:orange |

Show alternate structure
