# Supplementary material for: Homozygous EPRS1 missense variant causing hypomyelinating leukodystrophy-15 alters variant-distal mRNA m6A site accessibility
Source: Nat Commun. 2024 May 20;15:4284. doi: 10.1038/s41467-024-48549-x (PMC11106242; doi:10.1038/s41467-024-48549-x)
Supplement: Supplementary file 4 — Supplementary Software 1 [file 41467_2024_48549_MOESM4_ESM.zip › m6Ad-SNV-prediction/output/index/data/458192_NM_001305626.1.html]

RNAPlot - 458192 - NM\_001305626.1


## Target ID: 458192\_NM\_001305626.1

https://www.ncbi.nlm.nih.gov/clinvar/variation/458192/

https://www.ncbi.nlm.nih.gov/nuccore/NM\_001305626.1

#### Reference

|  |  |
| --- | --- |
| Sequence | TTAGTGCTGCAGAACTTCGCCATGTGATGACAAACCTTGGAGAGAAGTTAACAGATGAAGAAGTTGATGAAATGATCAGGGAAGCAGATATTGATGGTGATGGTCAAGTAAACTATGAAGGGTTTGTACAAATGATGACAGCAAAGTGAAGACCTTGTACAGAATGTGTTAAATTTCTTGTACAAAATTGTTTATTTGCCTTTTCTTTGTTTGTAACTTATCTGTAAAAGGTTTCTCCCTACTGTCAAAA |
| Base | G |
| Structure | ......((((..((((((..(((.((.((((...............)))).)).)))..))))))(((......)))......))))...(((((((((..((...((.(((((.....((((..((((((((((.((..(((((.((((....((((((((((..........))).))))))).....)))))))))....)).)))))))).))..))))......))))))).))))))))))).. |
| Colors | 12-16:green 28-32:green 49-53:green 110-114:green 136-140:green 150-154:green 214-218:green 47:orange |

Show reference structure

#### Alternate

|  |  |
| --- | --- |
| Sequence | TTAGTGCTGCAGAACTTCGCCATGTGATGACAAACCTTGGAGAGAAATTAACAGATGAAGAAGTTGATGAAATGATCAGGGAAGCAGATATTGATGGTGATGGTCAAGTAAACTATGAAGGGTTTGTACAAATGATGACAGCAAAGTGAAGACCTTGTACAGAATGTGTTAAATTTCTTGTACAAAATTGTTTATTTGCCTTTTCTTTGTTTGTAACTTATCTGTAAAAGGTTTCTCCCTACTGTCAAAA |
| Base | A |
| Structure | ..(((........)))((((...))))(((((......((.((((((((.((((((((.....(((((...((.(((((...........))))).))....))))).((((((......))))))(((((((((.((..(((((.((((....((((((((((..........))).))))))).....)))))))))....)).)))))))))..))))))))....))))))))))...)))))... |
| Colors | 12-16:green 28-32:green 49-53:green 110-114:green 136-140:green 150-154:green 214-218:green 47:orange |

Show alternate structure
