# Supplementary material for: Homozygous EPRS1 missense variant causing hypomyelinating leukodystrophy-15 alters variant-distal mRNA m6A site accessibility
Source: Nat Commun. 2024 May 20;15:4284. doi: 10.1038/s41467-024-48549-x (PMC11106242; doi:10.1038/s41467-024-48549-x)
Supplement: Supplementary file 4 — Supplementary Software 1 [file 41467_2024_48549_MOESM4_ESM.zip › m6Ad-SNV-prediction/output/index/data/543382_NM_001370298.3.html]

RNAPlot - 543382 - NM\_001370298.3


## Target ID: 543382\_NM\_001370298.3

https://www.ncbi.nlm.nih.gov/clinvar/variation/543382/

https://www.ncbi.nlm.nih.gov/nuccore/NM\_001370298.3

#### Reference

|  |  |
| --- | --- |
| Sequence | GCACAGCTTTGCTGCAGACAGTGAGGAACTGAAGCAGAAGTGGCTGAAAGTCATCCTTTTAGCTGTCACAGGTGAGACACCAGGTGGTCCAAATGAGCATCCAGCCACCTTGGATGATCATCCTGAACCTAAGAAAAAATCAGAATGCTGAACTCCTCCAGGACCAGCCATGGTGTGGAGGTCTCAGGACTTACAGCTCAAGACATTCCCAGCTCTTCTTACACATCTGCTAGCACTTTATGTTGAAAAA |
| Base | A |
| Structure | ...((((..(((((((((..(((((((.(((.........((((((.(((((....((((((..((..(((((((..(((((((((((...............)))))).))).)).))).)))).)))))))).............((((...((((((..((((....)))).))))))..))))))))).)))).)).........)))...)))))))...))))).))))......))))..... |
| Colors | 16-20:green 26-30:green 75-79:green 125-129:green 150-154:green 161-165:green 187-191:green 201-205:green 49:orange |

Show reference structure

#### Alternate

|  |  |
| --- | --- |
| Sequence | GCACAGCTTTGCTGCAGACAGTGAGGAACTGAAGCAGAAGTGGCTGAACGTCATCCTTTTAGCTGTCACAGGTGAGACACCAGGTGGTCCAAATGAGCATCCAGCCACCTTGGATGATCATCCTGAACCTAAGAAAAAATCAGAATGCTGAACTCCTCCAGGACCAGCCATGGTGTGGAGGTCTCAGGACTTACAGCTCAAGACATTCCCAGCTCTTCTTACACATCTGCTAGCACTTTATGTTGAAAAA |
| Base | C |
| Structure | ...((((..(((((((((((((.....))))....(((((.(((((((.(((.....((.(((((((((.((((...))))..)))......((((.(((((((.....))))))).))))(((((.............((((....))))...((((((..((((....)))).))))))..)))))....)))))).))))).)))..)))))))))......))))).))))......))))..... |
| Colors | 16-20:green 26-30:green 75-79:green 125-129:green 150-154:green 161-165:green 187-191:green 201-205:green 49:orange |

Show alternate structure
