# Supplementary material for: Homozygous EPRS1 missense variant causing hypomyelinating leukodystrophy-15 alters variant-distal mRNA m6A site accessibility
Source: Nat Commun. 2024 May 20;15:4284. doi: 10.1038/s41467-024-48549-x (PMC11106242; doi:10.1038/s41467-024-48549-x)
Supplement: Supplementary file 4 — Supplementary Software 1 [file 41467_2024_48549_MOESM4_ESM.zip › m6Ad-SNV-prediction/output/index/data/2888_NM_032531.4.html]

RNAPlot - 2888 - NM\_032531.4


## Target ID: 2888\_NM\_032531.4

https://www.ncbi.nlm.nih.gov/clinvar/variation/2888/

https://www.ncbi.nlm.nih.gov/nuccore/NM\_032531.4

#### Reference

|  |  |
| --- | --- |
| Sequence | GCGTCAGCAGCAGCGGCAAGCAGGATGGCTATGTGCAGTTCGACAAGGCCAGCAAGGCTTCTGCTTCCTCCTCCCACCACTCCCAGTCCTCGTCCCAGAACTCTGACCCCAGTCGACCCCTGCAGCGGCGGATGCAGACTCACGTCTAAGGATCACACACCGCGGGTGGGGACGGGCCAGGGAAGAGGTCAGGGCACGTTCTGGTTGTCCAGGGACGAGGGGTACTTTGCAGAGGACACCAGAATTGGCC |
| Base | G |
| Structure | ((....))...(((((.((((....(((((.(((........))).))))).....))))))))).((((.((((......(((.(((((((.(((.......(((((..((.((....(((((........))))).....)).))..)).)))........)))))))))))))...)))).))))....(((..((((((((.((((.....(((((....)))))....))))))))))))..))) |
| Colors | 98-102:green 104-108:green 136-140:green 234-238:green 3:orange |

Show reference structure

#### Alternate

|  |  |
| --- | --- |
| Sequence | GCTTCAGCAGCAGCGGCAAGCAGGATGGCTATGTGCAGTTCGACAAGGCCAGCAAGGCTTCTGCTTCCTCCTCCCACCACTCCCAGTCCTCGTCCCAGAACTCTGACCCCAGTCGACCCCTGCAGCGGCGGATGCAGACTCACGTCTAAGGATCACACACCGCGGGTGGGGACGGGCCAGGGAAGAGGTCAGGGCACGTTCTGGTTGTCCAGGGACGAGGGGTACTTTGCAGAGGACACCAGAATTGGCC |
| Base | T |
| Structure | .((((.((((.(((((.((((....(((((.(((........))).))))).....)))))))))(((((.((((...((..((((....((((((.((.((((..(((..(((...((((((.((((..(((..((((....))))....))).....))))..))))))...)))..))).)))).)).))).)))..))))..))...)))).))))).....)))).))))...(((....))).. |
| Colors | 98-102:green 104-108:green 136-140:green 234-238:green 3:orange |

Show alternate structure
