# Supplementary material for: Homozygous EPRS1 missense variant causing hypomyelinating leukodystrophy-15 alters variant-distal mRNA m6A site accessibility
Source: Nat Commun. 2024 May 20;15:4284. doi: 10.1038/s41467-024-48549-x (PMC11106242; doi:10.1038/s41467-024-48549-x)
Supplement: Supplementary file 4 — Supplementary Software 1 [file 41467_2024_48549_MOESM4_ESM.zip › m6Ad-SNV-prediction/output/index/data/454834_NM_012144.4.html]

RNAPlot - 454834 - NM\_012144.4


## Target ID: 454834\_NM\_012144.4

https://www.ncbi.nlm.nih.gov/clinvar/variation/454834/

https://www.ncbi.nlm.nih.gov/nuccore/NM\_012144.4

#### Reference

|  |  |
| --- | --- |
| Sequence | TGGGCACATCATCAGCCTCAAGCTCTCACCCAATTTGCGCAAGATGCCAAAGGAAAAGAAGGGGCAGGAGGTGCAGAAGGGTCCAGCTGTGGAGATTGCGAAACTGGACAAACTGCTGAACCTGGTGAGGGAAGTGAAAATCAAGACCTGAGGGGCTGGCCTCAGTCTCTGTCCCATCGCTTGAATACAGTACTCCTAGGGCTTGACCCTGGTACCCAGCCCAGCCTTAGCACCCAGCATGTGACCCCAC |
| Base | T |
| Structure | (((((.....(((((..(((((((((.((((..(((((((....((((..............))))....))))))).))))...((((((......((((....(((((....(((...(((....)))..)))........((((.(((((......))))))))).)))))..)))).....))))))......)))))))))..))))).....)))))......((.....))..(((....))) |
| Colors | 101-105:green 106-110:green 118-122:green 144-148:green 204-208:green 242-246:green 96:orange |

Show reference structure

#### Alternate

|  |  |
| --- | --- |
| Sequence | TGGGCACATCATCAGCCTCAAGCTCTCACCCAATTTGCGCAAGATGCCAAAGGAAAAGAAGGGGCAGGAGGTGCAGAAGGGTCCAGCTGTGGAGACTGCGAAACTGGACAAACTGCTGAACCTGGTGAGGGAAGTGAAAATCAAGACCTGAGGGGCTGGCCTCAGTCTCTGTCCCATCGCTTGAATACAGTACTCCTAGGGCTTGACCCTGGTACCCAGCCCAGCCTTAGCACCCAGCATGTGACCCCAC |
| Base | C |
| Structure | .((((((((...(((.(((.((((...((((..(((((((....((((..............))))....))))))).))))..))))...))).))).....((((......((((((..((((...(((((((((....((((((.(((((......))))))))).)).....)))))).............((((((.....))))))..)))...))))..)))))).)))).))))).)))... |
| Colors | 93-97:green 101-105:green 106-110:green 118-122:green 144-148:green 204-208:green 242-246:green 96:orange |

Show alternate structure
