# Supplementary material for: Homozygous EPRS1 missense variant causing hypomyelinating leukodystrophy-15 alters variant-distal mRNA m6A site accessibility
Source: Nat Commun. 2024 May 20;15:4284. doi: 10.1038/s41467-024-48549-x (PMC11106242; doi:10.1038/s41467-024-48549-x)
Supplement: Supplementary file 4 — Supplementary Software 1 [file 41467_2024_48549_MOESM4_ESM.zip › m6Ad-SNV-prediction/output/index/data/322845_NM_001374496.1.html]

RNAPlot - 322845 - NM\_001374496.1


## Target ID: 322845\_NM\_001374496.1

https://www.ncbi.nlm.nih.gov/clinvar/variation/322845/

https://www.ncbi.nlm.nih.gov/nuccore/NM\_001374496.1

#### Reference

|  |  |
| --- | --- |
| Sequence | CCAGTCCTACAACAACGACCAGTGGACGCTGATCTTCGGAGACCCAACCAAGTTTGGACTCGGGGTCTTCTCCATCGTCTTCGACGTCGTCTTCTTCATCCAGCACTTCTGTTTGTACAGAAAGAGACCGGGGTATGACCAGCTGAACTAGCACCCAGGGACCCAGTGTACCCAGCCTCTGGCCTCGTGCCCTGCTGGGGAAGGCCTCACCCAGCGAAGGCCGGAGAAGCGGTTGGGCCCTGGCACACAG |
| Base | T |
| Structure | ((.(((((......(((((....(((((.((......((((((((..(((....))).....))))))))..)).))))).....)))))................((((((....)))))))).))).))(((...))).((((...))))..((((((.((((....(((..(((((((((((.......(((((((((.....)).))))))).)))))))))..)))))))))))))))....... |
| Colors | 40-44:green 56-60:green 125-129:green 136-140:green 145-149:green 159-163:green 55:orange |

Show reference structure

#### Alternate

|  |  |
| --- | --- |
| Sequence | CCAGTCCTACAACAACGACCAGTGGACGCTGATCTTCGGAGACCCAACCAAGTTGGGACTCGGGGTCTTCTCCATCGTCTTCGACGTCGTCTTCTTCATCCAGCACTTCTGTTTGTACAGAAAGAGACCGGGGTATGACCAGCTGAACTAGCACCCAGGGACCCAGTGTACCCAGCCTCTGGCCTCGTGCCCTGCTGGGGAAGGCCTCACCCAGCGAAGGCCGGAGAAGCGGTTGGGCCCTGGCACACAG |
| Base | G |
| Structure | ...((((.((...........))))))((((.......(((.((((((...)))))).)))((((....)))).(((((((((..(((.((((((.((..(((.....)))..))...)).)))))))))))).)))).))))...........((((((.((((....(((..(((((((((((.......(((((((((.....)).))))))).)))))))))..)))))))))))))))....... |
| Colors | 40-44:green 56-60:green 125-129:green 136-140:green 145-149:green 159-163:green 55:orange |

Show alternate structure
