# Supplementary material for: Homozygous EPRS1 missense variant causing hypomyelinating leukodystrophy-15 alters variant-distal mRNA m6A site accessibility
Source: Nat Commun. 2024 May 20;15:4284. doi: 10.1038/s41467-024-48549-x (PMC11106242; doi:10.1038/s41467-024-48549-x)
Supplement: Supplementary file 4 — Supplementary Software 1 [file 41467_2024_48549_MOESM4_ESM.zip › m6Ad-SNV-prediction/output/index/data/126745_NM_001407304.1.html]

RNAPlot - 126745 - NM\_001407304.1


## Target ID: 126745\_NM\_001407304.1

https://www.ncbi.nlm.nih.gov/clinvar/variation/126745/

https://www.ncbi.nlm.nih.gov/nuccore/NM\_001407304.1

#### Reference

|  |  |
| --- | --- |
| Sequence | CCATTTGGGACTTACTTCTCGGTCAGTGTACTGCCCTCCTCCCACCTGTCTCTGACCAACATTGGTCTTTTGTGAAATGGTCGGGTACAGACTCTCATTTGCTGGCTGGACAAAAAGATGGAAATATATTTGTATACCACTATTCATAAGTTAGGGTAAAGTGAAAACACAATTTTCTGGATATATTGGGCCTCTTAGTATTTTTTGGAGTTTTAAATATAAAGGAGAATATCTGAATGACACTTAAAAT |
| Base | G |
| Structure | .(((((((((((........)))).((((((((((((................(((((....))))).((((((((.((((.(((((((((.((.(((((..((......))...)))))))......))))))).))))))))))))))..)))))..)))....)))).(((((((...((((((....((((...........)))).....))))))..)))))))..)))))))........... |
| Colors | 8-12:green 53-57:green 89-93:green 108-112:green 165-169:green 238-242:green 83:orange |

Show reference structure

#### Alternate

|  |  |
| --- | --- |
| Sequence | CCATTTGGGACTTACTTCTCGGTCAGTGTACTGCCCTCCTCCCACCTGTCTCTGACCAACATTGGTCTTTTGTGAAATGGTCAGGTACAGACTCTCATTTGCTGGCTGGACAAAAAGATGGAAATATATTTGTATACCACTATTCATAAGTTAGGGTAAAGTGAAAACACAATTTTCTGGATATATTGGGCCTCTTAGTATTTTTTGGAGTTTTAAATATAAAGGAGAATATCTGAATGACACTTAAAAT |
| Base | A |
| Structure | .(((((((((......))))((.(((....)))))(((((.....((((.((((((((.(((.........)))...)))))))).))))..((.(((((..((......))...)))))))..((((((((..((((.(((.........)))))))..(((....)))....(((((((.((((((((...))))))))..)))))))...)))))))).))))).......)))))........... |
| Colors | 8-12:green 53-57:green 89-93:green 108-112:green 165-169:green 238-242:green 83:orange |

Show alternate structure
