# Supplementary material for: Homozygous EPRS1 missense variant causing hypomyelinating leukodystrophy-15 alters variant-distal mRNA m6A site accessibility
Source: Nat Commun. 2024 May 20;15:4284. doi: 10.1038/s41467-024-48549-x (PMC11106242; doi:10.1038/s41467-024-48549-x)
Supplement: Supplementary file 4 — Supplementary Software 1 [file 41467_2024_48549_MOESM4_ESM.zip › m6Ad-SNV-prediction/output/index/data/489918_NM_001406657.1.html]

RNAPlot - 489918 - NM\_001406657.1


## Target ID: 489918\_NM\_001406657.1

https://www.ncbi.nlm.nih.gov/clinvar/variation/489918/

https://www.ncbi.nlm.nih.gov/nuccore/NM\_001406657.1

#### Reference

|  |  |
| --- | --- |
| Sequence | TTCCAGTGCACAGTTTGGATATTACTTTCGTGTAACCTGTAAGGAAGAAAAAGTCCTTCGTAACAATAAAAACTTTAGTACTGTAGATATCCAGAAGAATGGTGTTAAATTTACCAACAGGCTATGTAGAACCAATGCAGACACTCAATGATGTGTTAGCTCAGCTAGATGCTGTTGTCAGCTTTGCTCACGTGTCAAATGGAGCACCTGTTCCATATGTACGACCAGCCATTTTGGAGAAAGGACAAGG |
| Base | A |
| Structure | ...((((((..(((((((...((((......))))))(((...((((........))))...)))....)))))...))))))......(((((((...(((((.......)))))...((((.((((.......))))(((((........)))))(((..((((.(((......)))))))..)))..((((.((.(((((((....))))))).))))))...)))).)))))))............ |
| Colors | 33-37:green 61-65:green 70-74:green 129-133:green 139-143:green 243-247:green 12:orange |

Show reference structure

#### Alternate

|  |  |
| --- | --- |
| Sequence | TTCCAGTGCACCGTTTGGATATTACTTTCGTGTAACCTGTAAGGAAGAAAAAGTCCTTCGTAACAATAAAAACTTTAGTACTGTAGATATCCAGAAGAATGGTGTTAAATTTACCAACAGGCTATGTAGAACCAATGCAGACACTCAATGATGTGTTAGCTCAGCTAGATGCTGTTGTCAGCTTTGCTCACGTGTCAAATGGAGCACCTGTTCCATATGTACGACCAGCCATTTTGGAGAAAGGACAAGG |
| Base | C |
| Structure | .(((...(((((((((((((((((((...((......(((...((((........))))...)))......))...)))......))))))))....))))))))........((((..((((.((((.......))))(((((........)))))(((..((((.(((......)))))))..)))..((((.((.(((((((....))))))).))))))...))))...)))).....)))..... |
| Colors | 33-37:green 61-65:green 70-74:green 129-133:green 139-143:green 243-247:green 12:orange |

Show alternate structure
