# Supplementary material for: Homozygous EPRS1 missense variant causing hypomyelinating leukodystrophy-15 alters variant-distal mRNA m6A site accessibility
Source: Nat Commun. 2024 May 20;15:4284. doi: 10.1038/s41467-024-48549-x (PMC11106242; doi:10.1038/s41467-024-48549-x)
Supplement: Supplementary file 4 — Supplementary Software 1 [file 41467_2024_48549_MOESM4_ESM.zip › m6Ad-SNV-prediction/output/index/data/459218_NM_001198963.2.html]

RNAPlot - 459218 - NM\_001198963.2


## Target ID: 459218\_NM\_001198963.2

https://www.ncbi.nlm.nih.gov/clinvar/variation/459218/

https://www.ncbi.nlm.nih.gov/nuccore/NM\_001198963.2

#### Reference

|  |  |
| --- | --- |
| Sequence | GATACCTGTTTCCGAAGTTTACACTGTGCTGGACTGAGTTTGTAGACATGAAGGTCCATGTACCCTGTGAAACCCTCGAATACATTGAAGCCAACTATGGCAAGAATCAGCAGGGTGCATGATGCCATTTTAAGCTGCTTCACATCAGACTGAAATCCTAATTACAGTTCATAAGTGAAACAGACTAATTCAATGGCAATACCTTTTGTATAGGTCCTGTG |
| Base | T |
| Structure | ...((((((.......(((((((....(((......))).)))))))..((((((.((((((((((((((.....((((.....)))).((((....)))).....))).))))))))))).((((((((((..(((.(((((....(((((.(((....))).))))).....))))).)))..)))...)))))))..))))))...))))))...... |
| Colors | 31-35:green 44-48:green 70-74:green 147-151:green 178-182:green 7:orange |

Show reference structure

#### Alternate

|  |  |
| --- | --- |
| Sequence | GATACCCGTTTCCGAAGTTTACACTGTGCTGGACTGAGTTTGTAGACATGAAGGTCCATGTACCCTGTGAAACCCTCGAATACATTGAAGCCAACTATGGCAAGAATCAGCAGGGTGCATGATGCCATTTTAAGCTGCTTCACATCAGACTGAAATCCTAATTACAGTTCATAAGTGAAACAGACTAATTCAATGGCAATACCTTTTGTATAGGTCCTGTG |
| Base | C |
| Structure | ...........................((.(((((..........(((.((((((.((((((((((((((.....((((.....)))).((((....)))).....))).))))))))))).((((((((((..(((.(((((....(((((.(((....))).))))).....))))).)))..)))...)))))))..)))))))))...))))).)). |
| Colors | 31-35:green 44-48:green 70-74:green 147-151:green 178-182:green 7:orange |

Show alternate structure
