# Supplementary material for: Homozygous EPRS1 missense variant causing hypomyelinating leukodystrophy-15 alters variant-distal mRNA m6A site accessibility
Source: Nat Commun. 2024 May 20;15:4284. doi: 10.1038/s41467-024-48549-x (PMC11106242; doi:10.1038/s41467-024-48549-x)
Supplement: Supplementary file 4 — Supplementary Software 1 [file 41467_2024_48549_MOESM4_ESM.zip › m6Ad-SNV-prediction/output/index/data/527741_NM_001366624.2.html]

RNAPlot - 527741 - NM\_001366624.2


## Target ID: 527741\_NM\_001366624.2

https://www.ncbi.nlm.nih.gov/clinvar/variation/527741/

https://www.ncbi.nlm.nih.gov/nuccore/NM\_001366624.2

#### Reference

|  |  |
| --- | --- |
| Sequence | TCTCCGACCTGAAATGGATGCCCCTTTCTGGTGTAATCAAGGCGCTGCCTGCTTTTTTGAGGGAATTGATGATGTTCACTGGAAGGAAAATGGGACATTAGTTCAAGTAGCAACTATATCAGATGGAGAAACTGAGGCACAGAGAGGTTAAAGTTACACAGCTTGGAAACATGTTCAACCAAATGGCAAAGTGGGTGAAACAGGACAATGAAACAGGAATTTATTATGAGACATGGAATGTAAAAGCCAG |
| Base | T |
| Structure | ((((((..((((.((((.(((..(((.(((((((..(((((((...))))..((((((.((.((((.......)))).)).))))))...))).)))))))...)))..))).)))).)))).))))))((((..(((........)))..)))).(((.((((((............)))...)))...)))(((...(((...((.((...((...........))...))))...)))....))).. |
| Colors | 93-97:green 129-133:green 167-171:green 198-202:green 203-207:green 211-215:green 229-233:green 174:orange |

Show reference structure

#### Alternate

|  |  |
| --- | --- |
| Sequence | TCTCCGACCTGAAATGGATGCCCCTTTCTGGTGTAATCAAGGCGCTGCCTGCTTTTTTGAGGGAATTGATGATGTTCACTGGAAGGAAAATGGGACATTAGTTCAAGTAGCAACTATATCAGATGGAGAAACTGAGGCACAGAGAGGTTAAAGTTACACAGCTTGGAAACATGGTCAACCAAATGGCAAAGTGGGTGAAACAGGACAATGAAACAGGAATTTATTATGAGACATGGAATGTAAAAGCCAG |
| Base | G |
| Structure | ((((((..((((.((((.(((..(((.(((((((..(((((((...))))..((((((.((.((((.......)))).)).))))))...))).)))))))...)))..))).)))).)))).))))))......(((........(((...(((.(((.((((.......(((....))).......))))..))).)))..)))((((((.......)))))).....((((...))))....))).. |
| Colors | 93-97:green 129-133:green 167-171:green 198-202:green 203-207:green 211-215:green 229-233:green 174:orange |

Show alternate structure
