# Supplementary material for: Homozygous EPRS1 missense variant causing hypomyelinating leukodystrophy-15 alters variant-distal mRNA m6A site accessibility
Source: Nat Commun. 2024 May 20;15:4284. doi: 10.1038/s41467-024-48549-x (PMC11106242; doi:10.1038/s41467-024-48549-x)
Supplement: Supplementary file 4 — Supplementary Software 1 [file 41467_2024_48549_MOESM4_ESM.zip › m6Ad-SNV-prediction/output/index/data/698890_NM_201597.3.html]

RNAPlot - 698890 - NM\_201597.3


## Target ID: 698890\_NM\_201597.3

https://www.ncbi.nlm.nih.gov/clinvar/variation/698890/

https://www.ncbi.nlm.nih.gov/nuccore/NM\_201597.3

#### Reference

|  |  |
| --- | --- |
| Sequence | CGTGGATCGAGAGCAGGACCACAACGAGTGCAACAAGCAGCGCAGCCGTCATAAATCCAAGGATCGCTACTGTGAAAAGGATGGAGAAGTGATATCAAAAAAACGGAATGAGGCTGGGGAGTGGAACAGGGATGTTTACATCCGCCAATGAGTTTTGCCCGTTTGTGTTTTTTTTTTTTTTTTTTTGAAGTCTTGTATAACTAACAGCATCCCCAAAACAAAGTCTTTGGGGTCTACACTGCAATCATAT |
| Base | T |
| Structure | .(((((......(((((((..(((...((((........))))....(((((...((((....((((....))))......))))...)))))...((((((((((((((.(((((((((...(((((....)))))...))).))).........)))))))).)))))))))..........)))..)))))))..............(((((((........))))))))))))............. |
| Colors | 16-20:green 124-128:green 198-202:green 216-220:green 133:orange |

Show reference structure

#### Alternate

|  |  |
| --- | --- |
| Sequence | CGTGGATCGAGAGCAGGACCACAACGAGTGCAACAAGCAGCGCAGCCGTCATAAATCCAAGGATCGCTACTGTGAAAAGGATGGAGAAGTGATATCAAAAAAACGGAATGAGGCTGGGGAGTGGAACAGGGACGTTTACATCCGCCAATGAGTTTTGCCCGTTTGTGTTTTTTTTTTTTTTTTTTTGAAGTCTTGTATAACTAACAGCATCCCCAAAACAAAGTCTTTGGGGTCTACACTGCAATCATAT |
| Base | C |
| Structure | .(((((......(((((((..(((...((((........))))....(((((...((((....((((....))))......))))...)))))...((((((((((((((.(((((((((...((((......))))...))).))).........)))))))).)))))))))..........)))..)))))))..............(((((((........))))))))))))............. |
| Colors | 16-20:green 124-128:green 198-202:green 216-220:green 133:orange |

Show alternate structure
