# Supplementary material for: Homozygous EPRS1 missense variant causing hypomyelinating leukodystrophy-15 alters variant-distal mRNA m6A site accessibility
Source: Nat Commun. 2024 May 20;15:4284. doi: 10.1038/s41467-024-48549-x (PMC11106242; doi:10.1038/s41467-024-48549-x)
Supplement: Supplementary file 4 — Supplementary Software 1 [file 41467_2024_48549_MOESM4_ESM.zip › m6Ad-SNV-prediction/output/index/data/412459_NM_003000.3.html]

RNAPlot - 412459 - NM\_003000.3


## Target ID: 412459\_NM\_003000.3

https://www.ncbi.nlm.nih.gov/clinvar/variation/412459/

https://www.ncbi.nlm.nih.gov/nuccore/NM\_003000.3

#### Reference

|  |  |
| --- | --- |
| Sequence | CCAAGCTGCAGGACCCATTCTCTCTATACCGCTGCCACACCATCATGAACTGCACAAGGACCTGTCCTAAGGGTCTGAATCCAGGGAAAGCTATTGCAGAGATCAAGAAAATGATGGCAACCTATAAGGAGAAGAAAGCTTCAGTTTAACTGTTTCCATGCTAAACATGATTTATAACCAGCTCAGAGCTGAACATAATTTATATCTAATTTGAGTTCCTTTAAAGATCTTGGTTTTCCATGAATACAGC |
| Base | C |
| Structure | ..((((((.(((.....((((((.((((....(((((((...((.(((.(((((..((...((.((((..((((....)))).)))).))))..)))))...))).))...)).)))))...)))).)))))).....)))))))))..((((...((((..((((..(((((.(((..((((((((...((............))...))))))))...))).)))))...))))..))))...)))). |
| Colors | 11-15:green 47-51:green 58-62:green 147-151:green 163-167:green 175-179:green 191-195:green 67:orange |

Show reference structure

#### Alternate

|  |  |
| --- | --- |
| Sequence | CCAAGCTGCAGGACCCATTCTCTCTATACCGCTGCCACACCATCATGAACTGCACAAGGACCTGTCTTAAGGGTCTGAATCCAGGGAAAGCTATTGCAGAGATCAAGAAAATGATGGCAACCTATAAGGAGAAGAAAGCTTCAGTTTAACTGTTTCCATGCTAAACATGATTTATAACCAGCTCAGAGCTGAACATAATTTATATCTAATTTGAGTTCCTTTAAAGATCTTGGTTTTCCATGAATACAGC |
| Base | T |
| Structure | ..((((((.(((.....((((((.((((....(((((((...((.(((.(((((...((((((.......))))))..................)))))...))).))...)).)))))...)))).)))))).....)))))))))..((((...((((..((((..(((((.(((..((((((((...((............))...))))))))...))).)))))...))))..))))...)))). |
| Colors | 11-15:green 47-51:green 58-62:green 147-151:green 163-167:green 175-179:green 191-195:green 67:orange |

Show alternate structure
