# Supplementary material for: Homozygous EPRS1 missense variant causing hypomyelinating leukodystrophy-15 alters variant-distal mRNA m6A site accessibility
Source: Nat Commun. 2024 May 20;15:4284. doi: 10.1038/s41467-024-48549-x (PMC11106242; doi:10.1038/s41467-024-48549-x)
Supplement: Supplementary file 4 — Supplementary Software 1 [file 41467_2024_48549_MOESM4_ESM.zip › m6Ad-SNV-prediction/output/index/data/331658_NM_006186.4.html]

RNAPlot - 331658 - NM\_006186.4


## Target ID: 331658\_NM\_006186.4

https://www.ncbi.nlm.nih.gov/clinvar/variation/331658/

https://www.ncbi.nlm.nih.gov/nuccore/NM\_006186.4

#### Reference

|  |  |
| --- | --- |
| Sequence | ATTATTTGTCCAAACTGTTGGGGAAGCTCCCAGAACTTCGTACCCTTTGCACACAGGGGCTACAGCGCATTTTCTACCTGAAATTGGAAGACTTGGTGCCACCGCCAGCAATAATTGACAAACTTTTCCTGGACACTTTACCTTTCTAAGACCTCCTCCCAAGCACTTCAAAGGAACTGGAATGATAATGGAAACTGTCAAGAGGGGGCAAGTCACATGGGCAGAGATAGCCGTGTGAGCAGTCTCAGCT |
| Base | C |
| Structure | ....((((((.....((((((((..((..((((..(((((((.((((((....)))))).)))....((.((((.....)))).))))))..)))).))..)).))))))......))))))......((((((.(((..(((((((..(((....(((....((.((((.......)))).)).....)))....))).)))))))..)))((((((((..........))))))))...))).))).. |
| Colors | 12-16:green 33-37:green 89-93:green 116-120:green 131-135:green 149-153:green 174-178:green 192-196:green 11:orange |

Show reference structure

#### Alternate

|  |  |
| --- | --- |
| Sequence | ATTATTTGTCGAAACTGTTGGGGAAGCTCCCAGAACTTCGTACCCTTTGCACACAGGGGCTACAGCGCATTTTCTACCTGAAATTGGAAGACTTGGTGCCACCGCCAGCAATAATTGACAAACTTTTCCTGGACACTTTACCTTTCTAAGACCTCCTCCCAAGCACTTCAAAGGAACTGGAATGATAATGGAAACTGTCAAGAGGGGGCAAGTCACATGGGCAGAGATAGCCGTGTGAGCAGTCTCAGCT |
| Base | G |
| Structure | ....((((((((...((((((((..((..((((..(((((((.((((((....)))))).)))....((.((((.....)))).))))))..)))).))..)).))))))....))))))))......((((((.(((..(((((((..(((....(((....((.((((.......)))).)).....)))....))).)))))))..)))((((((((..........))))))))...))).))).. |
| Colors | 12-16:green 33-37:green 89-93:green 116-120:green 131-135:green 149-153:green 174-178:green 192-196:green 11:orange |

Show alternate structure
