# Supplementary material for: Homozygous EPRS1 missense variant causing hypomyelinating leukodystrophy-15 alters variant-distal mRNA m6A site accessibility
Source: Nat Commun. 2024 May 20;15:4284. doi: 10.1038/s41467-024-48549-x (PMC11106242; doi:10.1038/s41467-024-48549-x)
Supplement: Supplementary file 4 — Supplementary Software 1 [file 41467_2024_48549_MOESM4_ESM.zip › m6Ad-SNV-prediction/output/index/data/460345_NM_000143.4.html]

RNAPlot - 460345 - NM\_000143.4


## Target ID: 460345\_NM\_000143.4

https://www.ncbi.nlm.nih.gov/clinvar/variation/460345/

https://www.ncbi.nlm.nih.gov/nuccore/NM\_000143.4

#### Reference

|  |  |
| --- | --- |
| Sequence | ATATAGGGTATGACAAGGCAGCAAAGATTGCTAAGACAGCACACAAAAATGGATCAACCTTAAAGGAAACTGCTATCGAACTTGGCTATCTCACAGCAGAGCAGTTTGACGAATGGGTAAAACCTAAGGACATGCTGGGTCCAAAGTGATTTACATAAATTTATAATGAAAATAAACATGTATAAAATTTAAAAAAACAGACTCCCATTTCTTAAAAACGGATAAGTTTGAAAGGAAACTGCTATTGAAC |
| Base | T |
| Structure | .............((((((((.......((((.....))))........(((((((.(((...)))((((((((..........(((.......)))..))))))))......(((((...))))).((((.......))))....)))))))..(((((((((((((........))).))).)))))))................((((((..((((......))))..)))))).))))).)))... |
| Colors | 11-15:green 34-38:green 67-71:green 78-82:green 120-124:green 128-132:green 174-178:green 195-199:green 236-240:green 87:orange |

Show reference structure

#### Alternate

|  |  |
| --- | --- |
| Sequence | ATATAGGGTATGACAAGGCAGCAAAGATTGCTAAGACAGCACACAAAAATGGATCAACCTTAAAGGAAACTGCTATCGAACTTGGCCATCTCACAGCAGAGCAGTTTGACGAATGGGTAAAACCTAAGGACATGCTGGGTCCAAAGTGATTTACATAAATTTATAATGAAAATAAACATGTATAAAATTTAAAAAAACAGACTCCCATTTCTTAAAAACGGATAAGTTTGAAAGGAAACTGCTATTGAAC |
| Base | C |
| Structure | .....(((.(((.((((..(((((...)))))..((.((((.........((.....))...........)))).))...))))..))))))....(((((((((((...(((((((..........((((.......)))).((((....(((((...(((((.......))))).)))))....)))).............)))))))(((..((((......))))..))).)))))))).)))... |
| Colors | 11-15:green 34-38:green 67-71:green 78-82:green 120-124:green 128-132:green 174-178:green 195-199:green 236-240:green 87:orange |

Show alternate structure
