# Supplementary material for: Homozygous EPRS1 missense variant causing hypomyelinating leukodystrophy-15 alters variant-distal mRNA m6A site accessibility
Source: Nat Commun. 2024 May 20;15:4284. doi: 10.1038/s41467-024-48549-x (PMC11106242; doi:10.1038/s41467-024-48549-x)
Supplement: Supplementary file 4 — Supplementary Software 1 [file 41467_2024_48549_MOESM4_ESM.zip › m6Ad-SNV-prediction/output/index/data/11321_NM_004085.4.html]

RNAPlot - 11321 - NM\_004085.4


## Target ID: 11321\_NM\_004085.4

https://www.ncbi.nlm.nih.gov/clinvar/variation/11321/

https://www.ncbi.nlm.nih.gov/nuccore/NM\_004085.4

#### Reference

|  |  |
| --- | --- |
| Sequence | ACAAGCCTGGGCCAAAGTTGGACAGTCGGGCTGAGGCCTGTTTTGTGAACTGCGTTGAGCGCTTCATTGATACAAGCCAGTTCATCTTGAATCGACTGGAACAGACCCAGAAATCCAAGCCAGTTTTCTCAGAAAGCCTTTCTGACTGATCTCAGCATTACCTCTTTGGAAAAGGAAGGTAGTTCAAGAAATGAAGAGCTGTTGATGGGATGATTGAAGAAACAGCTATGAGAGGATTGGCTCCCATCTT |
| Base | C |
| Structure | ......((((((((....))).((((((......(((.(((...(((((.(((.....))).)))))....))).))).(((((...))))))))))).......))))).......((((((((((((((...(((.(((((.....((((((.((.((.(((((((......(((.....)))......))))))).)).)).))))))......)))))..))).))))))))))))))........ |
| Colors | 20-24:green 47-51:green 99-103:green 143-147:green 220-224:green 53:orange |

Show reference structure

#### Alternate

|  |  |
| --- | --- |
| Sequence | ACAAGCCTGGGCCAAAGTTGGACAGTCGGGCTGAGGCCTGTTTTGTGAACTGGGTTGAGCGCTTCATTGATACAAGCCAGTTCATCTTGAATCGACTGGAACAGACCCAGAAATCCAAGCCAGTTTTCTCAGAAAGCCTTTCTGACTGATCTCAGCATTACCTCTTTGGAAAAGGAAGGTAGTTCAAGAAATGAAGAGCTGTTGATGGGATGATTGAAGAAACAGCTATGAGAGGATTGGCTCCCATCTT |
| Base | G |
| Structure | (((.((((.((((...............)))).)))).)))...(((((((((.(((...............))).)))))))))..........((((.......)))).......((((((((((((((...(((.(((((.....((((((.((.((.(((((((......(((.....)))......))))))).)).)).))))))......)))))..))).))))))))))))))........ |
| Colors | 20-24:green 47-51:green 99-103:green 143-147:green 220-224:green 53:orange |

Show alternate structure
