# Supplementary material for: Homozygous EPRS1 missense variant causing hypomyelinating leukodystrophy-15 alters variant-distal mRNA m6A site accessibility
Source: Nat Commun. 2024 May 20;15:4284. doi: 10.1038/s41467-024-48549-x (PMC11106242; doi:10.1038/s41467-024-48549-x)
Supplement: Supplementary file 4 — Supplementary Software 1 [file 41467_2024_48549_MOESM4_ESM.zip › m6Ad-SNV-prediction/output/index/data/92588_NM_000181.4.html]

RNAPlot - 92588 - NM\_000181.4


## Target ID: 92588\_NM\_000181.4

https://www.ncbi.nlm.nih.gov/clinvar/variation/92588/

https://www.ncbi.nlm.nih.gov/nuccore/NM\_000181.4

#### Reference

|  |  |
| --- | --- |
| Sequence | GGAATAAAAAGGGGATCTTCACTCGGCAGAGACAACCAAAAAGTGCAGCGTTCCTTTTGCGAGAGAGATACTGGAAGATTGCCAATGAAACCAGGTATCCCCACTCAGTAGCCAAGTCACAATGTTTGGAAAACAGCCTGTTTACTTGAGCAAGACTGATACCACCTGCGTGTCCCTTCCTCCCCGAGTCAGGGCGACTTCCACAGCAGCAGAACAAGTGCCTCCTGGACTGTTCACGGCAGACCAGAAC |
| Base | T |
| Structure | ((((......((.(((((((.(((....)))..............(((.(((.((((....)))).))).)))))))))).))........(((((........(((((.(((((((.(((.(((((...)))))...)))..))))).))...)))))....))))).((..((((..(((...)))..))))..))))))......(((.......)))...((((.(((((...))))).))))... |
| Colors | 30-34:green 88-92:green 131-135:green 153-157:green 212-216:green 227-231:green 241-245:green 139:orange |

Show reference structure

#### Alternate

|  |  |
| --- | --- |
| Sequence | GGAATAAAAAGGGGATCTTCACTCGGCAGAGACAACCAAAAAGTGCAGCGTTCCTTTTGCGAGAGAGATACTGGAAGATTGCCAATGAAACCAGGTATCCCCACTCAGTAGCCAAGTCACAATGTTTGGAAAACAGCCCGTTTACTTGAGCAAGACTGATACCACCTGCGTGTCCCTTCCTCCCCGAGTCAGGGCGACTTCCACAGCAGCAGAACAAGTGCCTCCTGGACTGTTCACGGCAGACCAGAAC |
| Base | C |
| Structure | ((((......((.(((((((.(((....)))..............(((.(((.((((....)))).))).)))))))))).))........(((((........(((((.(((((((...((((.(((.....)))..)))).))))).))...)))))....))))).((..((((..(((...)))..))))..))))))......(((.......)))...((((.(((((...))))).))))... |
| Colors | 30-34:green 88-92:green 131-135:green 153-157:green 212-216:green 227-231:green 241-245:green 139:orange |

Show alternate structure
