# Supplementary material for: Homozygous EPRS1 missense variant causing hypomyelinating leukodystrophy-15 alters variant-distal mRNA m6A site accessibility
Source: Nat Commun. 2024 May 20;15:4284. doi: 10.1038/s41467-024-48549-x (PMC11106242; doi:10.1038/s41467-024-48549-x)
Supplement: Supplementary file 4 — Supplementary Software 1 [file 41467_2024_48549_MOESM4_ESM.zip › m6Ad-SNV-prediction/output/index/data/581116_NM_001265594.3.html]

RNAPlot - 581116 - NM\_001265594.3


## Target ID: 581116\_NM\_001265594.3

https://www.ncbi.nlm.nih.gov/clinvar/variation/581116/

https://www.ncbi.nlm.nih.gov/nuccore/NM\_001265594.3

#### Reference

|  |  |
| --- | --- |
| Sequence | TCCTCCAGCTGCTGGCAGGGGCTGGCACCCATGGGACACCCTCTGCCCCCAGCCGCAGCCTGTCAGAGCTCTGCCTGGCTGTTCCAGCCCCAGGACAGGAAGCTGACCCTGGCCCAGCTCTACCGAATCAGGACCACCCTGCTGCTTAACTCCACGCTCACTGCCTCGGAGGTCTGAGCAGAGGGAGGCCCCCAAGAGTGCCATTGACCAAGAGACAGCAGACAGCCTGCCTCCTGGGGCGTGCCGGCAC |
| Base | C |
| Structure | ......((((.((((((((((((((.......(((...))).......))))))....)))))))))))).((((.(((.......(((((((((((((..((((....((((...(((((.........((.((.((((.(((((((.((((..((.....))...))))...))))))))))).)).))...)))))))))............))))......))))..)))))))))..))))))). |
| Colors | 34-38:green 93-97:green 104-108:green 131-135:green 147-151:green 205-209:green 213-217:green 220-224:green 39:orange |

Show reference structure

#### Alternate

|  |  |
| --- | --- |
| Sequence | TCCTCCAGCTGCTGGCAGGGGCTGGCACCCATGGGACAGCCTCTGCCCCCAGCCGCAGCCTGTCAGAGCTCTGCCTGGCTGTTCCAGCCCCAGGACAGGAAGCTGACCCTGGCCCAGCTCTACCGAATCAGGACCACCCTGCTGCTTAACTCCACGCTCACTGCCTCGGAGGTCTGAGCAGAGGGAGGCCCCCAAGAGTGCCATTGACCAAGAGACAGCAGACAGCCTGCCTCCTGGGGCGTGCCGGCAC |
| Base | G |
| Structure | .......((((..(((((((((((...((...))..)))))))))))..)))).((((((.(((((...((((((((((((...)))))..))).))))...)))))..((((...(((((.........((.((.((((.(((((((.((((..((.....))...))))...))))))))))).)).))...)))))))))....(((.(((...((((.....))))))).)))))).)))...... |
| Colors | 34-38:green 93-97:green 104-108:green 131-135:green 147-151:green 205-209:green 213-217:green 220-224:green 39:orange |

Show alternate structure
