# Supplementary material for: Homozygous EPRS1 missense variant causing hypomyelinating leukodystrophy-15 alters variant-distal mRNA m6A site accessibility
Source: Nat Commun. 2024 May 20;15:4284. doi: 10.1038/s41467-024-48549-x (PMC11106242; doi:10.1038/s41467-024-48549-x)
Supplement: Supplementary file 4 — Supplementary Software 1 [file 41467_2024_48549_MOESM4_ESM.zip › m6Ad-SNV-prediction/output/index/data/476102_NM_173354.5.html]

RNAPlot - 476102 - NM\_173354.5


## Target ID: 476102\_NM\_173354.5

https://www.ncbi.nlm.nih.gov/clinvar/variation/476102/

https://www.ncbi.nlm.nih.gov/nuccore/NM\_173354.5

#### Reference

|  |  |
| --- | --- |
| Sequence | GCACCGGCCCCACCGCCCTCCCCGCTGTGCCCCCACCACGCCTGGCCAGGCTGGCCCCAGGTTGTGAGCCCCTGGGGCTGCTGCAGGGGGACTGTGAGATGGAGGACCTGATGCCCTGCTCCCTAGGCACGTTTGTCCTGGTGCAGTGAGGGCAGCCCTGCATCCTGGCACGGACACTGACTCTTACAGCAATAACTTCAGAGGAGGTGAAGACATCTGGCCTCAAAGCCAAGAACTTTCTAGAAGCGAA |
| Base | G |
| Structure | (((((....(((.(((..(((((.((((((.........((((....)))).(((((((((.........))))))))))).)))))))))..)))...)))(((((..((((.((((.....))))..)))).)))))))))).((((((((((.(((((......))).))...))).)))))))........((((..(((((((.......((((((......))).)))))))))).)))).... |
| Colors | 89-93:green 104-108:green 172-176:green 178-182:green 193-197:green 211-215:green 233-237:green 1:orange |

Show reference structure

#### Alternate

|  |  |
| --- | --- |
| Sequence | ACACCGGCCCCACCGCCCTCCCCGCTGTGCCCCCACCACGCCTGGCCAGGCTGGCCCCAGGTTGTGAGCCCCTGGGGCTGCTGCAGGGGGACTGTGAGATGGAGGACCTGATGCCCTGCTCCCTAGGCACGTTTGTCCTGGTGCAGTGAGGGCAGCCCTGCATCCTGGCACGGACACTGACTCTTACAGCAATAACTTCAGAGGAGGTGAAGACATCTGGCCTCAAAGCCAAGAACTTTCTAGAAGCGAA |
| Base | A |
| Structure | ...((((((....(((..(((((.((((((.........((((....)))).(((((((((.........))))))))))).)))))))))..))).((((.(((......(((((((((..(((((.((....)))))))...))).))))))..))).))))..))).))).((((..((((...((......))..))))..)))).(((..((((((......))).)))....)))......... |
| Colors | 89-93:green 104-108:green 172-176:green 178-182:green 193-197:green 211-215:green 233-237:green 1:orange |

Show alternate structure
