# Supplementary material for: Homozygous EPRS1 missense variant causing hypomyelinating leukodystrophy-15 alters variant-distal mRNA m6A site accessibility
Source: Nat Commun. 2024 May 20;15:4284. doi: 10.1038/s41467-024-48549-x (PMC11106242; doi:10.1038/s41467-024-48549-x)
Supplement: Supplementary file 4 — Supplementary Software 1 [file 41467_2024_48549_MOESM4_ESM.zip › m6Ad-SNV-prediction/output/index/data/49370_NM_001370405.1.html]

RNAPlot - 49370 - NM\_001370405.1


## Target ID: 49370\_NM\_001370405.1

https://www.ncbi.nlm.nih.gov/clinvar/variation/49370/

https://www.ncbi.nlm.nih.gov/nuccore/NM\_001370405.1

#### Reference

|  |  |
| --- | --- |
| Sequence | CGCCTACTCCAACCCCAGCCTACCTCTGGTGCACCCTCCGTCCCATAGCAAAGCCCCTGCACAGACTCCAGCCGAGCCCACACCTGGCTATGAGGTGGGCCAGCGGAAGCGCCTCATCTCCTCGGTGGAGGACTTCACCGAGTTTGTGTGAGGCCGGGGCCCTCCCTCCTGCACTGGCCTTGGACGGTATTGCCTGTCAGTGAAATAAATAAAGTCCTGACCCCAGTGCACAGACATAGAGGCACAGATT |
| Base | C |
| Structure | .................((((...((((.(((((...((((((...............((((((((((.....(((((..((((.((..((((((((..((...))...))))))))..))..))))..)).)))....))))))))))((((((((.((..........)).))))))))))))))........(((((.((...........)))))))....))))))))).....))))....... |
| Colors | 63-67:green 130-134:green 218-222:green 232-236:green 39:orange |

Show reference structure

#### Alternate

|  |  |
| --- | --- |
| Sequence | CGCCTACTCCAACCCCAGCCTACCTCTGGTGCACCCTCTGTCCCATAGCAAAGCCCCTGCACAGACTCCAGCCGAGCCCACACCTGGCTATGAGGTGGGCCAGCGGAAGCGCCTCATCTCCTCGGTGGAGGACTTCACCGAGTTTGTGTGAGGCCGGGGCCCTCCCTCCTGCACTGGCCTTGGACGGTATTGCCTGTCAGTGAAATAAATAAAGTCCTGACCCCAGTGCACAGACATAGAGGCACAGATT |
| Base | T |
| Structure | .((((.((....((((.(((((((((((((......(((((......(((.......)))))))).....))))((((.......))))..)))))))))..((....))(((((((...(((((((((....))))))))).....))))))).))))..........((((((((....((((..((((................))))..)))).....)))))))).......))))))....... |
| Colors | 63-67:green 130-134:green 218-222:green 232-236:green 39:orange |

Show alternate structure
