# Supplementary material for: Homozygous EPRS1 missense variant causing hypomyelinating leukodystrophy-15 alters variant-distal mRNA m6A site accessibility
Source: Nat Commun. 2024 May 20;15:4284. doi: 10.1038/s41467-024-48549-x (PMC11106242; doi:10.1038/s41467-024-48549-x)
Supplement: Supplementary file 4 — Supplementary Software 1 [file 41467_2024_48549_MOESM4_ESM.zip › m6Ad-SNV-prediction/output/index/data/336092_NM_001357321.2.html]

RNAPlot - 336092 - NM\_001357321.2


## Target ID: 336092\_NM\_001357321.2

https://www.ncbi.nlm.nih.gov/clinvar/variation/336092/

https://www.ncbi.nlm.nih.gov/nuccore/NM\_001357321.2

#### Reference

|  |  |
| --- | --- |
| Sequence | CTCAGGAGATAAGATCCTCAGTGTCATGGAGCTGGACTCGTACCCTCTCTACGCCATCTACCTCATCGTCATTGGCCTCAGCGGTGGCTTCATGGTCCTGTACTACGTGTCCTTAAGGTTCATCAAACAGAAACCAAGTCAAGACTGGTGATTCACGCCAGACGTCTGCCCGCTGGTGGGGGACCTGAGCAGACCCTTCAACTGCACTCCCTCCTCAGGAGCCCCTTCCTGGGGACAGTGAGGACAATGA |
| Base | T |
| Structure | ..............((((((.((((.(((((..((........)).))))).((((...((......))...)))).((((((((((((...((((.((((.....(((.((....))..)))...))))..)))))))))(((((((((.....)))))...))))..))))))).((((((.....((((.........))))..))))))((((((((....)))))))))))).))))))...... |
| Colors | 34-38:green 125-129:green 131-135:green 142-146:green 181-185:green 191-195:green 233-237:green 242-246:green 24:orange |

Show reference structure

#### Alternate

|  |  |
| --- | --- |
| Sequence | CTCAGGAGATAAGATCCTCAGTGCCATGGAGCTGGACTCGTACCCTCTCTACGCCATCTACCTCATCGTCATTGGCCTCAGCGGTGGCTTCATGGTCCTGTACTACGTGTCCTTAAGGTTCATCAAACAGAAACCAAGTCAAGACTGGTGATTCACGCCAGACGTCTGCCCGCTGGTGGGGGACCTGAGCAGACCCTTCAACTGCACTCCCTCCTCAGGAGCCCCTTCCTGGGGACAGTGAGGACAATGA |
| Base | C |
| Structure | ...((((.......))))(((.(((((((((((........(((((......((((...((......))...))))...)).)))))))))))))).))).......((((((...(((((........)).))).........((((((.....))))))..((((((.((..(((.....))))).))))))..........((((...((((((((((....)))))))))).)))))))))).... |
| Colors | 34-38:green 125-129:green 131-135:green 142-146:green 181-185:green 191-195:green 233-237:green 242-246:green 24:orange |

Show alternate structure
