# Supplementary material for: Homozygous EPRS1 missense variant causing hypomyelinating leukodystrophy-15 alters variant-distal mRNA m6A site accessibility
Source: Nat Commun. 2024 May 20;15:4284. doi: 10.1038/s41467-024-48549-x (PMC11106242; doi:10.1038/s41467-024-48549-x)
Supplement: Supplementary file 4 — Supplementary Software 1 [file 41467_2024_48549_MOESM4_ESM.zip › m6Ad-SNV-prediction/output/index/data/12564_NM_001252634.2.html]

RNAPlot - 12564 - NM\_001252634.2


## Target ID: 12564\_NM\_001252634.2

https://www.ncbi.nlm.nih.gov/clinvar/variation/12564/

https://www.ncbi.nlm.nih.gov/nuccore/NM\_001252634.2

#### Reference

|  |  |
| --- | --- |
| Sequence | ACGTGACACACTTTTGGCCAAAACTCCTGATGAAGGTGACAGATCTGCGGATGATAGGAGCCTGCCATGCCAGCCGCTTCCTGCACATGAAGGTGGAATGCCCCACAGAACTCTTCCCCCCTTTGTTCTTGGAAGTGTTCGAGGATTAGACTGACTGGATTCATTCTCATAATTCCTACAGCACTACTGGGTGTCATTTCATTCCATTGCCTAGCTCTTTTTTGTTTGTTTCTTTGTGTTGGGAGGGATT |
| Base | T |
| Structure | ..(((((((.....((((.....((((((((..((((.....))))....))..))))))...))))..(((((((((((........))).))))......(((.(((((.............)))))))).((((((..((((..(((.(((......))).)))......))))..)))))).))))))))))).....(((....((((((......................))))))..))).. |
| Colors | 4-8:green 21-25:green 37-41:green 108-112:green 148-152:green 136:orange |

Show reference structure

#### Alternate

|  |  |
| --- | --- |
| Sequence | ACGTGACACACTTTTGGCCAAAACTCCTGATGAAGGTGACAGATCTGCGGATGATAGGAGCCTGCCATGCCAGCCGCTTCCTGCACATGAAGGTGGAATGCCCCACAGAACTCTTCCCCCCTTTGTTCTTGGAAGCGTTCGAGGATTAGACTGACTGGATTCATTCTCATAATTCCTACAGCACTACTGGGTGTCATTTCATTCCATTGCCTAGCTCTTTTTTGTTTGTTTCTTTGTGTTGGGAGGGATT |
| Base | C |
| Structure | ..(((...)))............((((((((.((((.(((((((..((((.((.((((......)).)).)).))))..(((........)))..((((((.(((.(((((.............))))))))..)))))).((((..(((.(((......))).)))......)))).........(((((((..............))))))).........))))))).))))..))))))))..... |
| Colors | 4-8:green 21-25:green 37-41:green 108-112:green 148-152:green 136:orange |

Show alternate structure
