# Supplementary material for: Homozygous EPRS1 missense variant causing hypomyelinating leukodystrophy-15 alters variant-distal mRNA m6A site accessibility
Source: Nat Commun. 2024 May 20;15:4284. doi: 10.1038/s41467-024-48549-x (PMC11106242; doi:10.1038/s41467-024-48549-x)
Supplement: Supplementary file 4 — Supplementary Software 1 [file 41467_2024_48549_MOESM4_ESM.zip › m6Ad-SNV-prediction/output/index/data/213777_NM_001407012.1.html]

RNAPlot - 213777 - NM\_001407012.1


## Target ID: 213777\_NM\_001407012.1

https://www.ncbi.nlm.nih.gov/clinvar/variation/213777/

https://www.ncbi.nlm.nih.gov/nuccore/NM\_001407012.1

#### Reference

|  |  |
| --- | --- |
| Sequence | CGTCAAAGGCTGGGGAGCGGAGTACAGGAGACAGACTGTGACCAGTACCCCCTGCTGGATTGAGCTGCACCTGAATGGGCCTTTGCAGTGGCTTGACAAGGTCCTCACCCAGATGGGCTCCCCAAGCATCCGCTGTTCCAGTGTGTCTTAGAGACATCAAGTATGGTAGGGGAGGGCAGGCTTGGGGAAAATGGCCATGCAGGAGGTGGAGAAAATTGGAACTCTACTCAACCCATTGTTGTCAAGGAAG |
| Base | G |
| Structure | .......((((((((((.(((((((((........))))..((((((((.((((((((..((((....((((...((((((........))))))...)))).))))(((....))).(((((((((.(((.((.(((((..((((((...))))))......))).)).)).)))...)))))))))......))).))))).))).......))))).))))).)))..)))).....)))....... |
| Colors | 29-33:green 39-43:green 94-98:green 152-156:green 219-223:green 101:orange |

Show reference structure

#### Alternate

|  |  |
| --- | --- |
| Sequence | CGTCAAAGGCTGGGGAGCGGAGTACAGGAGACAGACTGTGACCAGTACCCCCTGCTGGATTGAGCTGCACCTGAATGGGCCTTTGCAGTGGCTTGACAAGTTCCTCACCCAGATGGGCTCCCCAAGCATCCGCTGTTCCAGTGTGTCTTAGAGACATCAAGTATGGTAGGGGAGGGCAGGCTTGGGGAAAATGGCCATGCAGGAGGTGGAGAAAATTGGAACTCTACTCAACCCATTGTTGTCAAGGAAG |
| Base | T |
| Structure | ...(((((((((((..((((..(((((........))))).((((((.....))))))......)))).))).....))))))))((((((.((((..(((((((((((......((((((((((((.(((.((.(((((..((((((...))))))......))).)).)).)))...))))))))).....)))........)))))........))))))....)))).))))))............ |
| Colors | 29-33:green 39-43:green 94-98:green 152-156:green 219-223:green 101:orange |

Show alternate structure
