# Supplementary material for: Homozygous EPRS1 missense variant causing hypomyelinating leukodystrophy-15 alters variant-distal mRNA m6A site accessibility
Source: Nat Commun. 2024 May 20;15:4284. doi: 10.1038/s41467-024-48549-x (PMC11106242; doi:10.1038/s41467-024-48549-x)
Supplement: Supplementary file 4 — Supplementary Software 1 [file 41467_2024_48549_MOESM4_ESM.zip › m6Ad-SNV-prediction/output/index/data/322846_NM_001031681.3.html]

RNAPlot - 322846 - NM\_001031681.3


## Target ID: 322846\_NM\_001031681.3

https://www.ncbi.nlm.nih.gov/clinvar/variation/322846/

https://www.ncbi.nlm.nih.gov/nuccore/NM\_001031681.3

#### Reference

|  |  |
| --- | --- |
| Sequence | CCAGCACTTCTGTTTGTACAGAAAGAGACCGGGGCTTCAGGCAGCGCGCACAGGCTCTGGCAGCCGTCTCAGGCAGGACTGGGCACCAAGCTTGCAGCCGAAGGCCTTGCCCCAAACTACCAGCGTTTCTGCAAGCAGCTTGAAGGGCTGACCTTGCAGCCGGGTGAGCCAAGGGCACTTTGCTGCCACCGCTGCATTCCCAGAGATCAAGCAGCCCGGTGCCGTGGCCAGTGAACTCAGAGGTGCTGGT |
| Base | G |
| Structure | ((((((((((((....(((...........(((((...((((..((.((.((((((.(((..(((((((......))))..))).)))))))))..))))...)))).)))))....((((..((...(((((((((((((...))))))..))))))).)))))).((((..((((((..(((((.....(((......))).......)))))..)))))).))))..)))....)))))))))))). |
| Colors | 26-30:green 76-80:green 114-118:green 149-153:green 233-237:green 31:orange |

Show reference structure

#### Alternate

|  |  |
| --- | --- |
| Sequence | CCAGCACTTCTGTTTGTACAGAAAGAGACCAGGGCTTCAGGCAGCGCGCACAGGCTCTGGCAGCCGTCTCAGGCAGGACTGGGCACCAAGCTTGCAGCCGAAGGCCTTGCCCCAAACTACCAGCGTTTCTGCAAGCAGCTTGAAGGGCTGACCTTGCAGCCGGGTGAGCCAAGGGCACTTTGCTGCCACCGCTGCATTCCCAGAGATCAAGCAGCCCGGTGCCGTGGCCAGTGAACTCAGAGGTGCTGGT |
| Base | A |
| Structure | ((((((((((((....(((.....(((((...((((((((..(((........))))))).))))))))).(((.(((((((((.....((((((((..(((.((.................)).))))))))))).((((((....(((....((((((..(((((((.(((....))).)))..))))))))))....)))...)))))).))))))).))...))).)))....)))))))))))). |
| Colors | 26-30:green 76-80:green 114-118:green 149-153:green 233-237:green 31:orange |

Show alternate structure
