# Supplementary material for: Homozygous EPRS1 missense variant causing hypomyelinating leukodystrophy-15 alters variant-distal mRNA m6A site accessibility
Source: Nat Commun. 2024 May 20;15:4284. doi: 10.1038/s41467-024-48549-x (PMC11106242; doi:10.1038/s41467-024-48549-x)
Supplement: Supplementary file 4 — Supplementary Software 1 [file 41467_2024_48549_MOESM4_ESM.zip › m6Ad-SNV-prediction/output/index/data/519710_NM_001406716.1.html]

RNAPlot - 519710 - NM\_001406716.1


## Target ID: 519710\_NM\_001406716.1

https://www.ncbi.nlm.nih.gov/clinvar/variation/519710/

https://www.ncbi.nlm.nih.gov/nuccore/NM\_001406716.1

#### Reference

|  |  |
| --- | --- |
| Sequence | TGGCTGGAACCTATTCATTACAAATCAGTAGTACTCCACTTTATAAAAAGAAAGAACTTAACCAACTAGAAGACAAATATGACAAAGACTACCTCAGTGGTGAACTGGGTGATAATCTGAAGATGAAAATCCAGGTTTTGCTTCATTAATTCACCATCCAGAGACCAAATAATTAAAAGAAAAACAAATATAGATAGGTAGAACTATATTTTCCCCCAATCAGAATCATCATATCATAGGTACAATCTTT |
| Base | T |
| Structure | .((.((((..(((((.(((...))).)))))...))))))...........................................(((((.(((((..(((((((.((((((((.....(((((..(((........)))..))))).....)))))..............................((((((((..........)))))))).........)))..))))))).....)))))...))))) |
| Colors | 7-11:green 54-58:green 59-63:green 71-75:green 80-84:green 86-90:green 102-106:green 162-166:green 182-186:green 201-205:green 136:orange |

Show reference structure

#### Alternate

|  |  |
| --- | --- |
| Sequence | TGGCTGGAACCTATTCATTACAAATCAGTAGTACTCCACTTTATAAAAAGAAAGAACTTAACCAACTAGAAGACAAATATGACAAAGACTACCTCAGTGGTGAACTGGGTGATAATCTGAAGATGAAAATCCAGGATTTGCTTCATTAATTCACCATCCAGAGACCAAATAATTAAAAGAAAAACAAATATAGATAGGTAGAACTATATTTTCCCCCAATCAGAATCATCATATCATAGGTACAATCTTT |
| Base | A |
| Structure | .((.((..((((((..(((((......)))))......((((........))))......................((((((..........(((.((((((((((((((....(((....)))....))))))((......)).....))))))))...))).............................(((.((..(((.......)))..)).))).......)))))).)))))).)).))... |
| Colors | 7-11:green 54-58:green 59-63:green 71-75:green 80-84:green 86-90:green 102-106:green 162-166:green 182-186:green 201-205:green 136:orange |

Show alternate structure
