# Supplementary material for: Homozygous EPRS1 missense variant causing hypomyelinating leukodystrophy-15 alters variant-distal mRNA m6A site accessibility
Source: Nat Commun. 2024 May 20;15:4284. doi: 10.1038/s41467-024-48549-x (PMC11106242; doi:10.1038/s41467-024-48549-x)
Supplement: Supplementary file 4 — Supplementary Software 1 [file 41467_2024_48549_MOESM4_ESM.zip › m6Ad-SNV-prediction/output/index/data/413352_NM_001321072.1.html]

RNAPlot - 413352 - NM\_001321072.1


## Target ID: 413352\_NM\_001321072.1

https://www.ncbi.nlm.nih.gov/clinvar/variation/413352/

https://www.ncbi.nlm.nih.gov/nuccore/NM\_001321072.1

#### Reference

|  |  |
| --- | --- |
| Sequence | TGGAGATGGACCACTTCGCCCTGGTGGTGCACGAGCAGATCCAGTGCCACAGCACCGGGAAGTCCAGTCAGCGGCAGATGGTGTTCGGGGTGGTCACCGCCATTGACTTGCTGAACTTCGTGGCCGCCCAGGAGCGGGACCAGAAGTGAAGTCCGGAGCGCTGGGCGGTGCGGAGCGGGCCCGCCACCCTTGCCCACTTCTCCTTCGCTTTCCTGAGCCCTAAACACACGCGTGATTGGTAACTGCCTGG |
| Base | T |
| Structure | ((((..((.((((((.......)))))).))........))))((((....))))((((.(((((((((((((......((.(((((((.(((((.((((..(((....((((.......))))....)))..)))))))))((((((((....((((...((((((((((((.......))).))))...)))))...)))))))))))))))))))))........))).)))))))..))).)))). |
| Colors | 8-12:green 104-108:green 113-117:green 137-141:green 222-226:green 240-244:green 68:orange |

Show reference structure

#### Alternate

|  |  |
| --- | --- |
| Sequence | TGGAGATGGACCACTTCGCCCTGGTGGTGCACGAGCAGATCCAGTGCCACAGCACCGGGAAGTCCAGCCAGCGGCAGATGGTGTTCGGGGTGGTCACCGCCATTGACTTGCTGAACTTCGTGGCCGCCCAGGAGCGGGACCAGAAGTGAAGTCCGGAGCGCTGGGCGGTGCGGAGCGGGCCCGCCACCCTTGCCCACTTCTCCTTCGCTTTCCTGAGCCCTAAACACACGCGTGATTGGTAACTGCCTGG |
| Base | C |
| Structure | (((...(((((........(((((((((((((...........))).)))..)))))))..))))).)))(((((..((((.((((((...(((((.......)))))..)))))).)))).)))))(((((((.(((..((((((((((....((((...((((((((((((.......))).))))...)))))...)))))))))))..)))..)))(((.(((....))).)))....)).))))) |
| Colors | 8-12:green 104-108:green 113-117:green 137-141:green 222-226:green 240-244:green 68:orange |

Show alternate structure
