# Supplementary material for: Homozygous EPRS1 missense variant causing hypomyelinating leukodystrophy-15 alters variant-distal mRNA m6A site accessibility
Source: Nat Commun. 2024 May 20;15:4284. doi: 10.1038/s41467-024-48549-x (PMC11106242; doi:10.1038/s41467-024-48549-x)
Supplement: Supplementary file 4 — Supplementary Software 1 [file 41467_2024_48549_MOESM4_ESM.zip › m6Ad-SNV-prediction/output/index/data/409527_NM_001406722.1.html]

RNAPlot - 409527 - NM\_001406722.1


## Target ID: 409527\_NM\_001406722.1

https://www.ncbi.nlm.nih.gov/clinvar/variation/409527/

https://www.ncbi.nlm.nih.gov/nuccore/NM\_001406722.1

#### Reference

|  |  |
| --- | --- |
| Sequence | TAGGACTGCTCCCACCAGTTCAGAAGATTATCTCAGACTGAAACGACGTTGTACTACATCTCTGATCAAAGAACAGGAGAGTTCCCAGGCCAGTACGGAAGAATGTGAGAAAAATAAGCAGGACACAATTACAACTAAAAAATATATCTAAGCATTTGCAAAGGCGACAATAAATTATTGACGCTTAACCTTTCCAGTTTATAAGACTGGAATATAATTTCAAACCACACATTAGTACTTATGTTGCACA |
| Base | G |
| Structure | ..(((....)))...(((((.(((......)))..)))))...((((((.((((((..(((((............))))).((((...........))))...(((((....................)))))..................((....))..(((((.(((((...))))).))))).....(((((((((...)))))))))....................))))))..)))))).... |
| Colors | 3-7:green 35-39:green 71-75:green 121-125:green 186-190:green 204-208:green 222-226:green 18:orange |

Show reference structure

#### Alternate

|  |  |
| --- | --- |
| Sequence | TAGGACTGCTCCCACCAATTCAGAAGATTATCTCAGACTGAAACGACGTTGTACTACATCTCTGATCAAAGAACAGGAGAGTTCCCAGGCCAGTACGGAAGAATGTGAGAAAAATAAGCAGGACACAATTACAACTAAAAAATATATCTAAGCATTTGCAAAGGCGACAATAAATTATTGACGCTTAACCTTTCCAGTTTATAAGACTGGAATATAATTTCAAACCACACATTAGTACTTATGTTGCACA |
| Base | A |
| Structure | ..(((((.((((.......(((((.(((.......(((.........))).......))))))))..........))))))))).(((...(((((.....((((((............................................((....))..(((((.(((((...))))).))))).....(((((((((...)))))))))...............)))))).)))))....))).... |
| Colors | 3-7:green 35-39:green 71-75:green 121-125:green 186-190:green 204-208:green 222-226:green 18:orange |

Show alternate structure
