# Supplementary material for: Homozygous EPRS1 missense variant causing hypomyelinating leukodystrophy-15 alters variant-distal mRNA m6A site accessibility
Source: Nat Commun. 2024 May 20;15:4284. doi: 10.1038/s41467-024-48549-x (PMC11106242; doi:10.1038/s41467-024-48549-x)
Supplement: Supplementary file 4 — Supplementary Software 1 [file 41467_2024_48549_MOESM4_ESM.zip › m6Ad-SNV-prediction/output/index/data/480318_NM_001406871.1.html]

RNAPlot - 480318 - NM\_001406871.1


## Target ID: 480318\_NM\_001406871.1

https://www.ncbi.nlm.nih.gov/clinvar/variation/480318/

https://www.ncbi.nlm.nih.gov/nuccore/NM\_001406871.1

#### Reference

|  |  |
| --- | --- |
| Sequence | AGTCGGTGATGATTGGGACTGCTCTTAACACAAGCGAGATGAAGAAACTGATCACCCACATGGGGGAGATGGACCACCCCTGGAACTGTCCCCATGGAAGGCCAACCATGAGACACATCGCCAACCTGGGTGTCATTTCTCAGAACTGACCGTAGTCACTGTATGGAATAATTGGTTTTATCGCAGATTTTTATGTTTTGAAAGACAGAGTCTTCACTAACCTTTTTTGTTTTAAAATGAACCTGCTACT |
| Base | C |
| Structure | (((.(((.....(((((((((((.........))))((.((((((..(((.((.(((....))).(((((((..(((((.(((...((((..(((((........))))).)))).....)))....))))))))))))(((((((((((....))))(((((((((((.....))))))).))))........)))))))..)))))..))))))))..........))))))).....))).)))... |
| Colors | 16-20:green 26-30:green 45-49:green 71-75:green 83-87:green 111-115:green 143-147:green 203-207:green 218-222:green 239-243:green 77:orange |

Show reference structure

#### Alternate

|  |  |
| --- | --- |
| Sequence | AGTCGGTGATGATTGGGACTGCTCTTAACACAAGCGAGATGAAGAAACTGATCACCCACATGGGGGAGATGGACCATCCCTGGAACTGTCCCCATGGAAGGCCAACCATGAGACACATCGCCAACCTGGGTGTCATTTCTCAGAACTGACCGTAGTCACTGTATGGAATAATTGGTTTTATCGCAGATTTTTATGTTTTGAAAGACAGAGTCTTCACTAACCTTTTTTGTTTTAAAATGAACCTGCTACT |
| Base | T |
| Structure | (((.(((.....(((((((((((.........))))((.((((((..(((.((.(((....)))((...(((.((.(((.(((........))).))).))))).)).(((((.....((((......)))).....)))))....((((....))))(((((((((((.....))))))).))))..(((((.....))))))))))..))))))))..........))))))).....))).)))... |
| Colors | 16-20:green 26-30:green 45-49:green 71-75:green 83-87:green 111-115:green 143-147:green 203-207:green 218-222:green 239-243:green 77:orange |

Show alternate structure
