# Supplementary material for: Homozygous EPRS1 missense variant causing hypomyelinating leukodystrophy-15 alters variant-distal mRNA m6A site accessibility
Source: Nat Commun. 2024 May 20;15:4284. doi: 10.1038/s41467-024-48549-x (PMC11106242; doi:10.1038/s41467-024-48549-x)
Supplement: Supplementary file 4 — Supplementary Software 1 [file 41467_2024_48549_MOESM4_ESM.zip › m6Ad-SNV-prediction/output/index/data/135603_NM_002136.4.html]

RNAPlot - 135603 - NM\_002136.4


## Target ID: 135603\_NM\_002136.4

https://www.ncbi.nlm.nih.gov/clinvar/variation/135603/

https://www.ncbi.nlm.nih.gov/nuccore/NM\_002136.4

#### Reference

|  |  |
| --- | --- |
| Sequence | AAATTTTGGACCCATGAAGGGAGGAAATTTTGGAGGCAGAAGCTCTGGCCCCTATGGCGGTGGAGGCCAATACTTTGCAAAACCACGAAACCAAGGTGGCTATGGCGGTTCCAGCAGCAGCAGTAGCTATGGCAGTGGCAGAAGATTTTAATTAGGAAACAAAGCTTAGCAGGAGAGGAGAGCCAGAGAAGTGACAGGGAAGCTACAGGTTACAACAGATTTGTGAACTCAGCCAAGCACAGTGGTGGCA |
| Base | A |
| Structure | (((((((...(((.....)))..)))))))(((((......(((.((((((((.(((..((((..((.((....))))....))))....)))))).))))).)))..)))))((.((.((.((.(((.((((..((((......((((..(((((........)))))...)))).....))))...........(((....((((((((......))))))))..))).))))))))).)).)).)). |
| Colors | 8-12:green 80-84:green 88-92:green 157-161:green 192-196:green 225-229:green 72:orange |

Show reference structure

#### Alternate

|  |  |
| --- | --- |
| Sequence | AAATTTTGGACCCATGAAGGGAGGAAATTTTGGAGGCAGAAGCTCTGGCCCCTATGGCGGTGGAGGCCAATGCTTTGCAAAACCACGAAACCAAGGTGGCTATGGCGGTTCCAGCAGCAGCAGTAGCTATGGCAGTGGCAGAAGATTTTAATTAGGAAACAAAGCTTAGCAGGAGAGGAGAGCCAGAGAAGTGACAGGGAAGCTACAGGTTACAACAGATTTGTGAACTCAGCCAAGCACAGTGGTGGCA |
| Base | G |
| Structure | (((((((...(((.....)))..)))))))(((..(((((.((..(((((.((((....)))).)))))..)))))))....)))....((((..(((.((.(((((((((((((...(((....)))...))..((((......((((..(((((........)))))...)))).....)))).....(((((.............)))))........)).)))))..)))))))))..)))).... |
| Colors | 8-12:green 80-84:green 88-92:green 157-161:green 192-196:green 225-229:green 72:orange |

Show alternate structure
