# Supplementary material for: Homozygous EPRS1 missense variant causing hypomyelinating leukodystrophy-15 alters variant-distal mRNA m6A site accessibility
Source: Nat Commun. 2024 May 20;15:4284. doi: 10.1038/s41467-024-48549-x (PMC11106242; doi:10.1038/s41467-024-48549-x)
Supplement: Supplementary file 4 — Supplementary Software 1 [file 41467_2024_48549_MOESM4_ESM.zip › m6Ad-SNV-prediction/output/index/data/36137_NM_001351498.2.html]

RNAPlot - 36137 - NM\_001351498.2


## Target ID: 36137\_NM\_001351498.2

https://www.ncbi.nlm.nih.gov/clinvar/variation/36137/

https://www.ncbi.nlm.nih.gov/nuccore/NM\_001351498.2

#### Reference

|  |  |
| --- | --- |
| Sequence | AAAACTTGATGTTTTTTTCTTCTATGAAGAAACTGATCACATGTGGAATGGAGGCACTCAGGCCAAAACAGGAAAAAAATTCAAGTATGAATCAAATAAGTACTTATTTATAAAGATACCTGTTTCCGAAGTTTACACTGTGCTGGACTGAGTTTGTAGACATGAAGGTCCATGTACCCTGTGAAACCCTCGAATACATTGAAGCCAACTATGGTAAGACCTGGAAGATTCCTGTAAAGACGTGGGACTG |
| Base | A |
| Structure | ......((((..(((((((......)))))))...))))(((((.....((((....(((((...(((((((......(((((....)))))...((((((....)))))).......))))))).(((..((((((..((((((((((.....((....))....)))))).))))..))))))....)))...........((((....))))....)))))....)))).......)))))...... |
| Colors | 2-6:green 30-34:green 66-70:green 145-149:green 158-162:green 184-188:green 217-221:green 245-249:green 240:orange |

Show reference structure

#### Alternate

|  |  |
| --- | --- |
| Sequence | AAAACTTGATGTTTTTTTCTTCTATGAAGAAACTGATCACATGTGGAATGGAGGCACTCAGGCCAAAACAGGAAAAAAATTCAAGTATGAATCAAATAAGTACTTATTTATAAAGATACCTGTTTCCGAAGTTTACACTGTGCTGGACTGAGTTTGTAGACATGAAGGTCCATGTACCCTGTGAAACCCTCGAATACATTGAAGCCAACTATGGTAAGACCTGGAAGATTCCTGTAAAGGCGTGGGACTG |
| Base | G |
| Structure | ......((((..(((((((......)))))))...))))(((.....)))......((((.(((...(((((((.....(((((((((.....(((((.((((((.......))).))).))))).(((..((((((..((((((((((.....((....))....)))))).))))..))))))....))).)))).)))))((((....)))).............)))))))...))).)))).... |
| Colors | 2-6:green 30-34:green 66-70:green 145-149:green 158-162:green 184-188:green 217-221:green 245-249:green 240:orange |

Show alternate structure
