# Supplementary material for: Homozygous EPRS1 missense variant causing hypomyelinating leukodystrophy-15 alters variant-distal mRNA m6A site accessibility
Source: Nat Commun. 2024 May 20;15:4284. doi: 10.1038/s41467-024-48549-x (PMC11106242; doi:10.1038/s41467-024-48549-x)
Supplement: Supplementary file 4 — Supplementary Software 1 [file 41467_2024_48549_MOESM4_ESM.zip › m6Ad-SNV-prediction/output/index/data/2224_NM_198156.3.html]

RNAPlot - 2224 - NM\_198156.3


## Target ID: 2224\_NM\_198156.3

https://www.ncbi.nlm.nih.gov/clinvar/variation/2224/

https://www.ncbi.nlm.nih.gov/nuccore/NM\_198156.3

#### Reference

|  |  |
| --- | --- |
| Sequence | GGTTGTCCGGAGCCTAGTCAAGCCTGAGAATTACAGGAGACTGGACATCGTCAGGTCGCTCTACGAAGATCTGGAAGACCACCCAAATGTGCAGAAAGACCTGGAGCGGCTGACACAGGAGCGCATTGCACATCAACGGATGGGAGATTGAAGATTTCTGTTGAAACTTACACTGTTTCATCTCAGCTTTTGATGGTACTGATGAGTCTTGATCTAGATACAGGACTGGTTCCTTCCTTAGTTTCAAAGT |
| Base | G |
| Structure | ((....))(((((((((((...((((.......)))).)))))).....(((((.((((((((......((((......(((......)))))))......)))))))))))))..((((((.((.(((.((((((.((.(((((................((((((.......))))))))))).)).))))))))).))...(((((((.........))))))).))))))......)))))..... |
| Colors | 38-42:green 43-47:green 76-80:green 97-101:green 111-115:green 164-168:green 223-227:green 5:orange |

Show reference structure

#### Alternate

|  |  |
| --- | --- |
| Sequence | GGTTTTCCGGAGCCTAGTCAAGCCTGAGAATTACAGGAGACTGGACATCGTCAGGTCGCTCTACGAAGATCTGGAAGACCACCCAAATGTGCAGAAAGACCTGGAGCGGCTGACACAGGAGCGCATTGCACATCAACGGATGGGAGATTGAAGATTTCTGTTGAAACTTACACTGTTTCATCTCAGCTTTTGATGGTACTGATGAGTCTTGATCTAGATACAGGACTGGTTCCTTCCTTAGTTTCAAAGT |
| Base | T |
| Structure | (((((((((((...(((....((((((((....(((....)))....)).))))))....)))......)))))))))))......((((((((.....((((............))))......))))))))....((((((((...(((((.((((....))))))).))...)))))))).((((...((.((....(((.(((((((.........))))))).))))).))...))))....... |
| Colors | 38-42:green 43-47:green 76-80:green 97-101:green 111-115:green 164-168:green 223-227:green 5:orange |

Show alternate structure
