# Supplementary material for: Homozygous EPRS1 missense variant causing hypomyelinating leukodystrophy-15 alters variant-distal mRNA m6A site accessibility
Source: Nat Commun. 2024 May 20;15:4284. doi: 10.1038/s41467-024-48549-x (PMC11106242; doi:10.1038/s41467-024-48549-x)
Supplement: Supplementary file 4 — Supplementary Software 1 [file 41467_2024_48549_MOESM4_ESM.zip › m6Ad-SNV-prediction/output/index/data/580161_NM_001142463.3.html]

RNAPlot - 580161 - NM\_001142463.3


## Target ID: 580161\_NM\_001142463.3

https://www.ncbi.nlm.nih.gov/clinvar/variation/580161/

https://www.ncbi.nlm.nih.gov/nuccore/NM\_001142463.3

#### Reference

|  |  |
| --- | --- |
| Sequence | GATCAAGAAGGAAAAGAAGAAGAGTAAGAAGGACAAGAAGGCCAAAGCTGGTCTGGAGAGCGGGGCCGAGCCTGGAGATGGGGACAGTGATACCACCAAGAAGAAGAAGAAGAAGAAGAAAGCAAAAGAGGTAGAATTGGTTTCTGAGTAGTGAAGGCCACTTGAAGCTGGAGGAGAAACTAAAGCCTTATTGAGAAAACATGTTATAGATCCTTTTGTTGCTGAGAGAGTGGAACATAGGTCCTAGACA |
| Base | G |
| Structure | ......................((((((.....((((..((((...((((.((.......((((......))))....(((((.........)).)))..................(((((.(((...........))).))))))).))))...)))).))))..(((..((......))..))))))))).........((((.(((..(((..(((((((.....)))..)))).)))..))))))) |
| Colors | 31-35:green 82-86:green 177-181:green 197-201:green 233-237:green 246-250:green 139:orange |

Show reference structure

#### Alternate

|  |  |
| --- | --- |
| Sequence | GATCAAGAAGGAAAAGAAGAAGAGTAAGAAGGACAAGAAGGCCAAAGCTGGTCTGGAGAGCGGGGCCGAGCCTGGAGATGGGGACAGTGATACCACCAAGAAGAAGAAGAAGAAGAAGAAAGCAAAAGAGGTAGAATTCGTTTCTGAGTAGTGAAGGCCACTTGAAGCTGGAGGAGAAACTAAAGCCTTATTGAGAAAACATGTTATAGATCCTTTTGTTGCTGAGAGAGTGGAACATAGGTCCTAGACA |
| Base | C |
| Structure | ......................((((((.....((((..((((...((((.((.......((((......)))).....(((((((((..((((...................................))))..))).)))))))).))))...)))).))))..(((..((......))..))))))))).........((((.(((..(((..(((((((.....)))..)))).)))..))))))) |
| Colors | 31-35:green 82-86:green 177-181:green 197-201:green 233-237:green 246-250:green 139:orange |

Show alternate structure
