# Supplementary material for: Homozygous EPRS1 missense variant causing hypomyelinating leukodystrophy-15 alters variant-distal mRNA m6A site accessibility
Source: Nat Commun. 2024 May 20;15:4284. doi: 10.1038/s41467-024-48549-x (PMC11106242; doi:10.1038/s41467-024-48549-x)
Supplement: Supplementary file 4 — Supplementary Software 1 [file 41467_2024_48549_MOESM4_ESM.zip › m6Ad-SNV-prediction/output/index/data/446216_NM_182972.3.html]

RNAPlot - 446216 - NM\_182972.3


## Target ID: 446216\_NM\_182972.3

https://www.ncbi.nlm.nih.gov/clinvar/variation/446216/

https://www.ncbi.nlm.nih.gov/nuccore/NM\_182972.3

#### Reference

|  |  |
| --- | --- |
| Sequence | AACAGCAGGGAGCTAGTGGAGAGGTCTATTGTCCCAGTGGGGAAAAATGCCCTCTTGTGGGCTCCAATGTCCCCTGGGCCTTTATGCAAGGGGAAATTGCAACCATCCTTGCTGGAGATGTGAAAGTGAAAAAAGAGAGAGACTCGTGACTTTTCCGGTTTCAGAAAAACCCAATGATTACCCTTAATTAAAACTGCTTGAATTGTATATATATCTCCATATATATATATATCCAAGACAAGGGAAATGT |
| Base | G |
| Structure | .(((((.....))).))(((((((((....(((((((((((((....(((((((((((((((.(((........)))))))....)))))))).....)))....)))))))))).)))...............(((.....)))..)))))))))(((((.....))))).....(((.((((..........((((((...(((((((((((....)))))))))))....)))).)))))).))).. |
| Colors | 140-144:green 147-151:green 167-171:green 191-195:green 236-240:green 37:orange |

Show reference structure

#### Alternate

|  |  |
| --- | --- |
| Sequence | AACAGCAGGGAGCTAGTGGAGAGGTCTATTGTCCCAATGGGGAAAAATGCCCTCTTGTGGGCTCCAATGTCCCCTGGGCCTTTATGCAAGGGGAAATTGCAACCATCCTTGCTGGAGATGTGAAAGTGAAAAAAGAGAGAGACTCGTGACTTTTCCGGTTTCAGAAAAACCCAATGATTACCCTTAATTAAAACTGCTTGAATTGTATATATATCTCCATATATATATATATCCAAGACAAGGGAAATGT |
| Base | A |
| Structure | ..(((((((((..(((((((....)))))))((((....)))).....((((......))))..((((.((((((..((......)).)))))).))))......)))))))))(((((..((((((.......(((.....)))...))))))...)))))..............(((.((((..........((((((...(((((((((((....)))))))))))....)))).)))))).))).. |
| Colors | 140-144:green 147-151:green 167-171:green 191-195:green 236-240:green 37:orange |

Show alternate structure
