# Supplementary material for: Homozygous EPRS1 missense variant causing hypomyelinating leukodystrophy-15 alters variant-distal mRNA m6A site accessibility
Source: Nat Commun. 2024 May 20;15:4284. doi: 10.1038/s41467-024-48549-x (PMC11106242; doi:10.1038/s41467-024-48549-x)
Supplement: Supplementary file 4 — Supplementary Software 1 [file 41467_2024_48549_MOESM4_ESM.zip › m6Ad-SNV-prediction/output/index/data/1291049_NM_001144881.2.html]

RNAPlot - 1291049 - NM\_001144881.2


## Target ID: 1291049\_NM\_001144881.2

https://www.ncbi.nlm.nih.gov/clinvar/variation/1291049/

https://www.ncbi.nlm.nih.gov/nuccore/NM\_001144881.2

#### Reference

|  |  |
| --- | --- |
| Sequence | TCCCTCAACCCCGGCCACAGAGATTTGCCTACTATACAGATGACCTTTCTAGTCCACCATCTGCACTTCCCACCCCTCAGTTTGGTCAGAGGACAACAAAATCCAAGAAGAAAAAGGGACACAAGGGCAAAAATTGTATTATTTCTTAACCAAGTAGTGATGCAGAGCATTTGTTTAAAACTTAGCCATTAACCGTCTGAATCGTTTCCTTTTCTTCCGTAGGAAAGTTGTGAAAATAGTTTAAAGTGCT |
| Base | T |
| Structure | ............(((...........)))........(((((...............)))))((((((......((((..........))))(((((....(((..((((((((.(((((....((((......(((((((((.(((....))).)))))))))((((....))))..................)))).....))))).))))))))....)))..)))))............)))))). |
| Colors | 41-45:green 91-95:green 117-121:green 147-151:green 178-182:green 190-194:green 240:orange |

Show reference structure

#### Alternate

|  |  |
| --- | --- |
| Sequence | TCCCTCAACCCCGGCCACAGAGATTTGCCTACTATACAGATGACCTTTCTAGTCCACCATCTGCACTTCCCACCCCTCAGTTTGGTCAGAGGACAACAAAATCCAAGAAGAAAAAGGGACACAAGGGCAAAAATTGTATTATTTCTTAACCAAGTAGTGATGCAGAGCATTTGTTTAAAACTTAGCCATTAACCGTCTGAATCGTTTCCTTTTCTTCCGTAGGAAAGTTGTGAAAATAGCTTAAAGTGCT |
| Base | C |
| Structure | ............(((...........)))........(((((...............)))))((((((......((((..........)))).........(((..((((((((.(((((....((((......(((((((((.(((....))).)))))))))((((....))))..................)))).....))))).))))))))....)))(((((((....))))))).)))))). |
| Colors | 41-45:green 91-95:green 117-121:green 147-151:green 178-182:green 190-194:green 240:orange |

Show alternate structure
