# Supplementary material for: Homozygous EPRS1 missense variant causing hypomyelinating leukodystrophy-15 alters variant-distal mRNA m6A site accessibility
Source: Nat Commun. 2024 May 20;15:4284. doi: 10.1038/s41467-024-48549-x (PMC11106242; doi:10.1038/s41467-024-48549-x)
Supplement: Supplementary file 4 — Supplementary Software 1 [file 41467_2024_48549_MOESM4_ESM.zip › m6Ad-SNV-prediction/output/index/data/387732_NM_001406648.1.html]

RNAPlot - 387732 - NM\_001406648.1


## Target ID: 387732\_NM\_001406648.1

https://www.ncbi.nlm.nih.gov/clinvar/variation/387732/

https://www.ncbi.nlm.nih.gov/nuccore/NM\_001406648.1

#### Reference

|  |  |
| --- | --- |
| Sequence | CCCTAAGCATGTAATAGAGTGTGCTAAACAGAAAGCCCTGGAACTTGAGGAGTTTCAGTATATTGGAGAATCGCAAGGATATGATATCATGGAACCAGCAGCAAAGAAGTGCTATCTGGAAAGAGAGATGATTAAAGTTTATCAATTGTAAAGGTGGAGGAATTTGGGAACTAGACAGTGCACACATAAATAATAAATATGTTCTTCAAATATTGGGTGGGCTAATGTGGGAGGAGTTTGAGACCAGCCT |
| Base | G |
| Structure | ...(((((..........((((((.............(((((((((...)))))))))............((.((..((((...)))).)))).((((.((((......))))..)))).......(((((.......))))).(((((..((.................))..))))))))))).................(((((..(((((((.....)))))))..)))))))))).......... |
| Colors | 26-30:green 41-45:green 92-96:green 168-172:green 173-177:green 241-245:green 52:orange |

Show reference structure

#### Alternate

|  |  |
| --- | --- |
| Sequence | CCCTAAGCATGTAATAGAGTGTGCTAAACAGAAAGCCCTGGAACTTGAGGAATTTCAGTATATTGGAGAATCGCAAGGATATGATATCATGGAACCAGCAGCAAAGAAGTGCTATCTGGAAAGAGAGATGATTAAAGTTTATCAATTGTAAAGGTGGAGGAATTTGGGAACTAGACAGTGCACACATAAATAATAAATATGTTCTTCAAATATTGGGTGGGCTAATGTGGGAGGAGTTTGAGACCAGCCT |
| Base | A |
| Structure | ...(((((..........((((((....(((......)))...((((..((.((((.........)))).)).))))((((...))))......((((.((((......))))..)))).......(((((.......))))).(((((..((.................))..))))))))))).................(((((..(((((((.....)))))))..)))))))))).......... |
| Colors | 26-30:green 41-45:green 92-96:green 168-172:green 173-177:green 241-245:green 52:orange |

Show alternate structure
