# Supplementary material for: Homozygous EPRS1 missense variant causing hypomyelinating leukodystrophy-15 alters variant-distal mRNA m6A site accessibility
Source: Nat Commun. 2024 May 20;15:4284. doi: 10.1038/s41467-024-48549-x (PMC11106242; doi:10.1038/s41467-024-48549-x)
Supplement: Supplementary file 4 — Supplementary Software 1 [file 41467_2024_48549_MOESM4_ESM.zip › m6Ad-SNV-prediction/output/index/data/485069_NM_003977.4.html]

RNAPlot - 485069 - NM\_003977.4


## Target ID: 485069\_NM\_003977.4

https://www.ncbi.nlm.nih.gov/clinvar/variation/485069/

https://www.ncbi.nlm.nih.gov/nuccore/NM\_003977.4

#### Reference

|  |  |
| --- | --- |
| Sequence | CCAGGAGGCCCAGGCTGACTTTGCCAAAGTGCTGGAGCTGGACCCAGCCCTGGCGCCTGTGGTGAGCCGAGAGCTGCGGGCCCTGGAGGCACGGATCCGGCAGAAGGACGAAGAGGACAAAGCCCGGTTCCGGGGGATCTTCTCCCATTGACAGGAGCACTTGGCCCTGCCTTACCTGCCAAGCCCACTGCTGCAGCTGCCAGCCCCCCTGCCCGTGCTGCGTCATGCTTCTGTGTATATAAAGGCCTTT |
| Base | A |
| Structure | ...(((((((..(((((.(((((((...((.((...((((((((..(((((((.(((((((((.........))))))))).)))).)))..)).))))))...)).)).....)).)))))..)))))..((((((...))))))....(((((((((((((((............)))))).....((.((((((.((..((......))..)))))))).))))))))))).........))))))) |
| Colors | 16-20:green 40-44:green 115-119:green 149-153:green 121:orange |

Show reference structure

#### Alternate

|  |  |
| --- | --- |
| Sequence | CCAGGAGGCCCAGGCTGACTTTGCCAAAGTGCTGGAGCTGGACCCAGCCCTGGCGCCTGTGGTGAGCCGAGAGCTGCGGGCCCTGGAGGCACGGATCCGGCAGAAGGACGAAGAGGACAATGCCCGGTTCCGGGGGATCTTCTCCCATTGACAGGAGCACTTGGCCCTGCCTTACCTGCCAAGCCCACTGCTGCAGCTGCCAGCCCCCCTGCCCGTGCTGCGTCATGCTTCTGTGTATATAAAGGCCTTT |
| Base | T |
| Structure | (((((..((((.((((...(((((((..(((((((.((((....)))).)))))))...))))))).....))))..)))))))))((((..(((.((((((..................)).)))).)))((((((...))))))....(((((((((((((((............)))))).....((.((((((.((..((......))..)))))))).)))))))))))..........)))).. |
| Colors | 16-20:green 40-44:green 115-119:green 149-153:green 121:orange |

Show alternate structure
