# Supplementary material for: Homozygous EPRS1 missense variant causing hypomyelinating leukodystrophy-15 alters variant-distal mRNA m6A site accessibility
Source: Nat Commun. 2024 May 20;15:4284. doi: 10.1038/s41467-024-48549-x (PMC11106242; doi:10.1038/s41467-024-48549-x)
Supplement: Supplementary file 4 — Supplementary Software 1 [file 41467_2024_48549_MOESM4_ESM.zip › m6Ad-SNV-prediction/output/index/data/404269_NM_004064.5.html]

RNAPlot - 404269 - NM\_004064.5


## Target ID: 404269\_NM\_004064.5

https://www.ncbi.nlm.nih.gov/clinvar/variation/404269/

https://www.ncbi.nlm.nih.gov/nuccore/NM\_004064.5

#### Reference

|  |  |
| --- | --- |
| Sequence | AGGAATAAGGAAGCGACCTGCAACCGACGATTCTTCTACTCAAAACAAAAGAGCCAACAGAACAGAAGAAAATGTTTCAGACGGTTCCCCAAATGCCGGTTCTGTGGAGCAGACGCCCAAGAAGCCTGGCCTCAGAAGACGTCAAACGTAAACAGCTCGAATTAAGAATATGTTTCCTTGTTTATCAGATACATCACTGCTTGATGAAGCAAGGAAGATATACATGAAAATTTTAAAAATACATATCGCT |
| Base | G |
| Structure | ...........(((((..((.....((((..((((((.............(((((....(((((........))))).....))))).......(((((((((.(((.((....))))))).)))).)))...)))))))))).............((.......))((((.((((((((((((((((.((......)).)))))).))))))))))))))....................))..))))) |
| Colors | 43-47:green 60-64:green 150-154:green 84:orange |

Show reference structure

#### Alternate

|  |  |
| --- | --- |
| Sequence | AGGAATAAGGAAGCGACCTGCAACCGACGATTCTTCTACTCAAAACAAAAGAGCCAACAGAACAGAAGAAAATGTTTCAGACGCTTCCCCAAATGCCGGTTCTGTGGAGCAGACGCCCAAGAAGCCTGGCCTCAGAAGACGTCAAACGTAAACAGCTCGAATTAAGAATATGTTTCCTTGTTTATCAGATACATCACTGCTTGATGAAGCAAGGAAGATATACATGAAAATTTTAAAAATACATATCGCT |
| Base | C |
| Structure | ...........(((((..((..........(((((((..((..................))..))))))).((((((..((((((((.......(((((((((.(((.((....))))))).)))).)))....)))).)))))))))).......((.......))((((.((((((((((((((((.((......)).)))))).))))))))))))))....................))..))))) |
| Colors | 43-47:green 60-64:green 150-154:green 84:orange |

Show alternate structure
