# Supplementary material for: Homozygous EPRS1 missense variant causing hypomyelinating leukodystrophy-15 alters variant-distal mRNA m6A site accessibility
Source: Nat Commun. 2024 May 20;15:4284. doi: 10.1038/s41467-024-48549-x (PMC11106242; doi:10.1038/s41467-024-48549-x)
Supplement: Supplementary file 4 — Supplementary Software 1 [file 41467_2024_48549_MOESM4_ESM.zip › m6Ad-SNV-prediction/output/index/data/630954_NM_001142571.2.html]

RNAPlot - 630954 - NM\_001142571.2


## Target ID: 630954\_NM\_001142571.2

https://www.ncbi.nlm.nih.gov/clinvar/variation/630954/

https://www.ncbi.nlm.nih.gov/nuccore/NM\_001142571.2

#### Reference

|  |  |
| --- | --- |
| Sequence | CCATCGAGGGAGCAGGAGCATCAGGCGGCCGGCGCATGGCGTGTCTGGCCAAATCTTCCCGACAGGCAACAGGTTTCCAGGAGATGGTAGACATTGGGACCTGGGGGACCTCAGAGCAGAGTGCCACATTACAGGGTGATCAGACATGACCTGTGCTGTTGTTTGGGAAACAGGGAAGCATTGGGGACCCCTCCCAACTTTTCTTCCCAGTAACGCCTGCTGTTTACTGCCACCTGGCACTGGTGACTAC |
| Base | G |
| Structure | .((((((((..(((((((((.((((((..(((((((.(((((((((((.((.....((((..((((...((((((((((.....))).)))).)))...)))).)))).(((......)))..((........)).)).))))))))).)))))))))....((((((...(((((((..((((((.....)))))))))))))))))))...)))))).))))).))))..))).....)))))..... |
| Colors | 89-93:green 97-101:green 106-110:green 142-146:green 147-151:green 168-172:green 185-189:green 244-248:green 73:orange |

Show reference structure

#### Alternate

|  |  |
| --- | --- |
| Sequence | CCATCGAGGGAGCAGGAGCATCAGGCGGCCGGCGCATGGCGTGTCTGGCCAAATCTTCCCGACAGGCAACAGTTTTCCAGGAGATGGTAGACATTGGGACCTGGGGGACCTCAGAGCAGAGTGCCACATTACAGGGTGATCAGACATGACCTGTGCTGTTGTTTGGGAAACAGGGAAGCATTGGGGACCCCTCCCAACTTTTCTTCCCAGTAACGCCTGCTGTTTACTGCCACCTGGCACTGGTGACTAC |
| Base | T |
| Structure | .((((((((..(((((((((.((((((..(((((((.(((((((((((.((......(((....((((...((((((((((.((((.....))))....))))))))))(((......)))))))........))))).))))))))).)))))))))....((((((...(((((((..((((((.....)))))))))))))))))))...)))))).))))).))))..))).....)))))..... |
| Colors | 89-93:green 97-101:green 106-110:green 142-146:green 147-151:green 168-172:green 185-189:green 244-248:green 73:orange |

Show alternate structure
