# Supplementary material for: Homozygous EPRS1 missense variant causing hypomyelinating leukodystrophy-15 alters variant-distal mRNA m6A site accessibility
Source: Nat Commun. 2024 May 20;15:4284. doi: 10.1038/s41467-024-48549-x (PMC11106242; doi:10.1038/s41467-024-48549-x)
Supplement: Supplementary file 4 — Supplementary Software 1 [file 41467_2024_48549_MOESM4_ESM.zip › m6Ad-SNV-prediction/output/index/data/540173_NM_001350626.2.html]

RNAPlot - 540173 - NM\_001350626.2


## Target ID: 540173\_NM\_001350626.2

https://www.ncbi.nlm.nih.gov/clinvar/variation/540173/

https://www.ncbi.nlm.nih.gov/nuccore/NM\_001350626.2

#### Reference

|  |  |
| --- | --- |
| Sequence | TGGCCATGCTCAGGTCCCTAGACCTGGAGGGCCTGCGGAGCACGCTGGCCGAGAGCAGCGACCACGTGGAAAAGAGTCCCCAGTCCCTCCTGCAGGACATGCTGGCCACGGGAGGCTTCCTGCAGGGGGACGAGGCCGACTGCTACTGAGCAGAACCAGAGTCTGCCACTGGGGCTCAGGACCAAGGGAGGCAGCACCATGTCCTTCTGTGGGACACTGCCAGCCCCAGGGCTCCAGCCCAGCCCGGTGG |
| Base | C |
| Structure | .((((....(((((((....)))))))..))))..(((.((.(((((........))))).....((((...(((.((((..(((((.(((((((((......((((......))))))))))))))))))..))....(((((....))))).....)).))).))))((((((((((..(((.((((((((......)).)))))).)))....)))..)))))))((((....)))).))))).... |
| Colors | 20-24:green 95-99:green 153-157:green 179-183:green 212-216:green 78:orange |

Show reference structure

#### Alternate

|  |  |
| --- | --- |
| Sequence | TGGCCATGCTCAGGTCCCTAGACCTGGAGGGCCTGCGGAGCACGCTGGCCGAGAGCAGCGACCACGTGGAAAAGAGTTCCCAGTCCCTCCTGCAGGACATGCTGGCCACGGGAGGCTTCCTGCAGGGGGACGAGGCCGACTGCTACTGAGCAGAACCAGAGTCTGCCACTGGGGCTCAGGACCAAGGGAGGCAGCACCATGTCCTTCTGTGGGACACTGCCAGCCCCAGGGCTCCAGCCCAGCCCGGTGG |
| Base | T |
| Structure | ((((((((((((((((((((....))).))))))...)))))...))))))(((((((((.((....((((.....))))..(((((.(((((((((......((((......))))))))))))))))))..)).)).))))).))..(((((.......)))))((((((((((..(((((..(((.(((((.....((((((.....)))))))))))...)))..)).))))))....))))))). |
| Colors | 20-24:green 95-99:green 153-157:green 179-183:green 212-216:green 78:orange |

Show alternate structure
