# Supplementary material for: Homozygous EPRS1 missense variant causing hypomyelinating leukodystrophy-15 alters variant-distal mRNA m6A site accessibility
Source: Nat Commun. 2024 May 20;15:4284. doi: 10.1038/s41467-024-48549-x (PMC11106242; doi:10.1038/s41467-024-48549-x)
Supplement: Supplementary file 4 — Supplementary Software 1 [file 41467_2024_48549_MOESM4_ESM.zip › m6Ad-SNV-prediction/output/index/data/264270_NM_001352303.2.html]

RNAPlot - 264270 - NM\_001352303.2


## Target ID: 264270\_NM\_001352303.2

https://www.ncbi.nlm.nih.gov/clinvar/variation/264270/

https://www.ncbi.nlm.nih.gov/nuccore/NM\_001352303.2

#### Reference

|  |  |
| --- | --- |
| Sequence | GCCAGCTGCAGGTCACCTGGGAGGACAGCACCACCGGCAAGGAGGACACGGGCACCTTTGACACCGTCCTGTGGGCCATAGGACCTTGCATCTCTGCGTGTCTCCCCACCACCGTGGGACATGCTGGAAAAAACCAGAGAAGAGACTGAGACGGCATCAGCCAGGTGTCCTCATCGAGGATCAACTAGGCAATCATCCTCGCCTTCCCTGGCCCTTGAGCAATTGCTTATTAAGGTTTCAGCACATAAAT |
| Base | C |
| Structure | ((..((((..(((...(((......))).)))..))))...((((((((.(((............((((((((...))))))))..(((.((((.....((((((((((....))))).....((((......))))....))))).))))..)))...)))..))))))))...((((((.............))))))(((......)))((((((((....)))))...))).....))........ |
| Colors | 23-27:green 44-48:green 59-63:green 81-85:green 117-121:green 131-135:green 143-147:green 49:orange |

Show reference structure

#### Alternate

|  |  |
| --- | --- |
| Sequence | GCCAGCTGCAGGTCACCTGGGAGGACAGCACCACCGGCAAGGAGGACATGGGCACCTTTGACACCGTCCTGTGGGCCATAGGACCTTGCATCTCTGCGTGTCTCCCCACCACCGTGGGACATGCTGGAAAAAACCAGAGAAGAGACTGAGACGGCATCAGCCAGGTGTCCTCATCGAGGATCAACTAGGCAATCATCCTCGCCTTCCCTGGCCCTTGAGCAATTGCTTATTAAGGTTTCAGCACATAAAT |
| Base | T |
| Structure | ....((((.(((.(((((((((((...((.(((((........))...))))).))))((.((..((((((((...))))))))..))))(((((((((((...(((((....)))))))))))(((......)))....)))))((((.......))))))))))).)))....((((((.............))))))(((......)))((((((((....)))))...)))...))))........ |
| Colors | 23-27:green 44-48:green 59-63:green 81-85:green 117-121:green 131-135:green 143-147:green 49:orange |

Show alternate structure
