# Supplementary material for: Homozygous EPRS1 missense variant causing hypomyelinating leukodystrophy-15 alters variant-distal mRNA m6A site accessibility
Source: Nat Commun. 2024 May 20;15:4284. doi: 10.1038/s41467-024-48549-x (PMC11106242; doi:10.1038/s41467-024-48549-x)
Supplement: Supplementary file 4 — Supplementary Software 1 [file 41467_2024_48549_MOESM4_ESM.zip › m6Ad-SNV-prediction/output/index/data/31194_NM_177965.4.html]

RNAPlot - 31194 - NM\_177965.4


## Target ID: 31194\_NM\_177965.4

https://www.ncbi.nlm.nih.gov/clinvar/variation/31194/

https://www.ncbi.nlm.nih.gov/nuccore/NM\_177965.4

#### Reference

|  |  |
| --- | --- |
| Sequence | ACATGCCAGAATTTCACAAATTAAAAGCAAAGTTGATAAAGAAGAAAGGAACACGGGCATATGCCTGCCAGTGTAGCTGGAGAACTATTGAAGAAGTGACTGACCTTCAGACAGATCATCAGCTTCGCTGGGTTTGTGGTAAACATTAAGAATGCAGACTGCACATTCGGACAGATGCATCCATGAGAGTGGTCTCCATGAATCATCATCATGTTTAGACAATAGTCCCTAGCAATACCATTCCCTGTGG |
| Base | C |
| Structure | ...(((.((....((((((((..........(((...............))).(((((....)))))((((((.((((((.((.((.((((((..((.....))))))))..)).)).)))))).))))))))))))))..........(((((.........)))))(((((((((.((.((((.(((....))))))).))...)))).))))).(((....))).)).)))...((((.....)))) |
| Colors | 49-53:green 82-86:green 97-101:green 109-113:green 141-145:green 156-160:green 169-173:green 217-221:green 54:orange |

Show reference structure

#### Alternate

|  |  |
| --- | --- |
| Sequence | ACATGCCAGAATTTCACAAATTAAAAGCAAAGTTGATAAAGAAGAAAGGAACATGGGCATATGCCTGCCAGTGTAGCTGGAGAACTATTGAAGAAGTGACTGACCTTCAGACAGATCATCAGCTTCGCTGGGTTTGTGGTAAACATTAAGAATGCAGACTGCACATTCGGACAGATGCATCCATGAGAGTGGTCTCCATGAATCATCATCATGTTTAGACAATAGTCCCTAGCAATACCATTCCCTGTGG |
| Base | T |
| Structure | ...(((.((..........(((((........)))))...........((((((((((....)))..((((((.((((((.((.((.((((((..((.....))))))))..)).)).)))))).))))))...((((((...((((...))))...))))))..........((((.((.((((.(((....))))))).))))))..))))))).(((....))).)).)))...((((.....)))) |
| Colors | 49-53:green 82-86:green 97-101:green 109-113:green 141-145:green 156-160:green 169-173:green 217-221:green 54:orange |

Show alternate structure
