# Supplementary material for: Homozygous EPRS1 missense variant causing hypomyelinating leukodystrophy-15 alters variant-distal mRNA m6A site accessibility
Source: Nat Commun. 2024 May 20;15:4284. doi: 10.1038/s41467-024-48549-x (PMC11106242; doi:10.1038/s41467-024-48549-x)
Supplement: Supplementary file 4 — Supplementary Software 1 [file 41467_2024_48549_MOESM4_ESM.zip › m6Ad-SNV-prediction/output/index/data/623464_NM_182763.3.html]

RNAPlot - 623464 - NM\_182763.3


## Target ID: 623464\_NM\_182763.3

https://www.ncbi.nlm.nih.gov/clinvar/variation/623464/

https://www.ncbi.nlm.nih.gov/nuccore/NM\_182763.3

#### Reference

|  |  |
| --- | --- |
| Sequence | ACCACGAGACGGCCTTCCAAGGATGGGTTTGTGGAGTTCTTCCATGTAGAGGACCTAGAAGGTGGCATCAGGAATGTGCTGCTGGCTTTTGCAGGTGTTGCTGGAGTAGGAGCTGGTTTGGCATATCTAATAAGATAGCCTTACTGTAAGTGCAATAGTTGACTTTTAACCAACCACCACCACCACCAAAACCAGTTTATGCAGTTGGACTCCAAGCTGTAACTTCCTAGAGTTGCACCCTAGCAACCTA |
| Base | G |
| Structure | .((((((....((((((....)).)))))))))).......((.((((((((.((.....(((((((.((....))))))))))))))))))))).((((((((.(((.(((((((((((((.(((((....)))))))).........(((.....((((.........))))......))).....)))))))))).)))..((((...))))..((((((((....))))))))..))))))))... |
| Colors | 51-55:green 160-164:green 167-171:green 189-193:green 207-211:green 220-224:green 116:orange |

Show reference structure

#### Alternate

|  |  |
| --- | --- |
| Sequence | ACCACGAGACGGCCTTCCAAGGATGGGTTTGTGGAGTTCTTCCATGTAGAGGACCTAGAAGGTGGCATCAGGAATGTGCTGCTGGCTTTTGCAGGTGTTGCTGGAGTAGGAGCTGTTTTGGCATATCTAATAAGATAGCCTTACTGTAAGTGCAATAGTTGACTTTTAACCAACCACCACCACCACCAAAACCAGTTTATGCAGTTGGACTCCAAGCTGTAACTTCCTAGAGTTGCACCCTAGCAACCTA |
| Base | T |
| Structure | .((((((....((((((....)).)))))))))).((((((.......))))))((((.((((((((.((....))))))(((((.(((((..((((.((.(((((((((.(((((((((..........)))))))))))))))..((((.(((...)))))))........))).)).))))..))))))))))...(((((((((...)).))))))))))).)))).(((((......)))))... |
| Colors | 51-55:green 160-164:green 167-171:green 189-193:green 207-211:green 220-224:green 116:orange |

Show alternate structure
