# Supplementary material for: Homozygous EPRS1 missense variant causing hypomyelinating leukodystrophy-15 alters variant-distal mRNA m6A site accessibility
Source: Nat Commun. 2024 May 20;15:4284. doi: 10.1038/s41467-024-48549-x (PMC11106242; doi:10.1038/s41467-024-48549-x)
Supplement: Supplementary file 4 — Supplementary Software 1 [file 41467_2024_48549_MOESM4_ESM.zip › m6Ad-SNV-prediction/output/index/data/6465_NM_001003722.2.html]

RNAPlot - 6465 - NM\_001003722.2


## Target ID: 6465\_NM\_001003722.2

https://www.ncbi.nlm.nih.gov/clinvar/variation/6465/

https://www.ncbi.nlm.nih.gov/nuccore/NM\_001003722.2

#### Reference

|  |  |
| --- | --- |
| Sequence | GAATTGAAGCTATCACAAGCTCAGGACAGATGGGCTCCTTCATACGCCTCAAGCAGTTCTTGGAGAAATGTTTGCAACACAAGGACATTCCTGTCCCCAAGGGCTTTCTGACTTCCTCCTTCTGGCGCTCCTGATGTCACTCCATCACCCACCATCACCGCTGCTGCAAAGAGGCAATAATAAAGGAACTGAAGACAGCTGTATTTGGGAGAAGTCATGTCAGATTCAGAAAT |
| Base | T |
| Structure | ...((((.(((......)))))))(((((((...(((((..((((((.....))(((((((((.(((((((((.........)))))))....)).)))))))))..(((.((((.(((((.(((......(((((......)))))....))).....(((.((....)).))).......)))))...)))).)))..))))..)))))..))).))))............ |
| Colors | 24-28:green 83-87:green 109-113:green 186-190:green 193-197:green 88:orange |

Show reference structure

#### Alternate

|  |  |
| --- | --- |
| Sequence | GAATTGAAGCTATCACAAGCTCAGGACAGATGGGCTCCTTCATACGCCTCAAGCAGTTCTTGGAGAAATGTTTGCAACACAAGGACACTCCTGTCCCCAAGGGCTTTCTGACTTCCTCCTTCTGGCGCTCCTGATGTCACTCCATCACCCACCATCACCGCTGCTGCAAAGAGGCAATAATAAAGGAACTGAAGACAGCTGTATTTGGGAGAAGTCATGTCAGATTCAGAAAT |
| Base | C |
| Structure | ...((((.(((......)))))))(((((((...(((((..((((((......((((((((((((...(((((.........))))))))).((((....))))..........((((.((.((((((...(((((................)))))..)).)))).)).)))).........))))))))......)).))))..)))))..))).))))............ |
| Colors | 24-28:green 83-87:green 109-113:green 186-190:green 193-197:green 88:orange |

Show alternate structure
