# Supplementary material for: Homozygous EPRS1 missense variant causing hypomyelinating leukodystrophy-15 alters variant-distal mRNA m6A site accessibility
Source: Nat Commun. 2024 May 20;15:4284. doi: 10.1038/s41467-024-48549-x (PMC11106242; doi:10.1038/s41467-024-48549-x)
Supplement: Supplementary file 4 — Supplementary Software 1 [file 41467_2024_48549_MOESM4_ESM.zip › m6Ad-SNV-prediction/output/index/data/486801_NM_001406566.1.html]

RNAPlot - 486801 - NM\_001406566.1


## Target ID: 486801\_NM\_001406566.1

https://www.ncbi.nlm.nih.gov/clinvar/variation/486801/

https://www.ncbi.nlm.nih.gov/nuccore/NM\_001406566.1

#### Reference

|  |  |
| --- | --- |
| Sequence | GTCTAATCGGTGGAACAGTGATGAGTGTCTACGAGCAGTTTTGAAGCTAATGTCAGAATGCTGGGCCCACAATCCAGCCTCCAGACTCACAGCATTGAGAATTAAGAAGACGCTTGCCAAGATGGTTGAATCCCAAGATGTAAAAATCTGATGGTTAAACCATCGGAGGAGAAACTCTAGACTGCAAGAACTGTTTTTACCCATGGCATGGGTGGAATTAGAGTGGAATAAGGATGTTAACTTGGTTCTC |
| Base | G |
| Structure | (((((((((.((...)).))))((((((((......((((((.(((((...(((.....((((((.......)))))).....)))....))).)).))))))....)))))))).....(((((((.((((...((((......))))...)))).))))))).(((......))))))))........(((.((((((((((...)))))))))).)))...((((((((........))).))))). |
| Colors | 13-17:green 83-87:green 157-161:green 172-176:green 179-183:green 188-192:green 238-242:green 27:orange |

Show reference structure

#### Alternate

|  |  |
| --- | --- |
| Sequence | GTCTAATCGGTGGAACAGTGATGAGTTTCTACGAGCAGTTTTGAAGCTAATGTCAGAATGCTGGGCCCACAATCCAGCCTCCAGACTCACAGCATTGAGAATTAAGAAGACGCTTGCCAAGATGGTTGAATCCCAAGATGTAAAAATCTGATGGTTAAACCATCGGAGGAGAAACTCTAGACTGCAAGAACTGTTTTTACCCATGGCATGGGTGGAATTAGAGTGGAATAAGGATGTTAACTTGGTTCTC |
| Base | T |
| Structure | .((((((((((....((((...((((((((.(((((((((((.(((((...(((.....((((((.......)))))).....)))....))).)).))))))........)))))((..(((((((.((((...((((......))))...)))).)))))))...))))))))))...)))).....))))..(((((((((...)))))))))))))))..((((((((........))).))))). |
| Colors | 13-17:green 83-87:green 157-161:green 172-176:green 179-183:green 188-192:green 238-242:green 27:orange |

Show alternate structure
