# Supplementary material for: Homozygous EPRS1 missense variant causing hypomyelinating leukodystrophy-15 alters variant-distal mRNA m6A site accessibility
Source: Nat Commun. 2024 May 20;15:4284. doi: 10.1038/s41467-024-48549-x (PMC11106242; doi:10.1038/s41467-024-48549-x)
Supplement: Supplementary file 4 — Supplementary Software 1 [file 41467_2024_48549_MOESM4_ESM.zip › m6Ad-SNV-prediction/output/index/data/222976_NM_001017369.3.html]

RNAPlot - 222976 - NM\_001017369.3


## Target ID: 222976\_NM\_001017369.3

https://www.ncbi.nlm.nih.gov/clinvar/variation/222976/

https://www.ncbi.nlm.nih.gov/nuccore/NM\_001017369.3

#### Reference

|  |  |
| --- | --- |
| Sequence | TGCTGGTTCTCGGCATCATGATTTCCACCACATGAACTTCATTGGAAACTATGCTTCAACATTTACATGGTGGGATCGAATTTTTGGAACAGACTCTCAGTATAATGCCTATAATGAAAAGAGGAAGAAGTTTGAGAAAAAGACTGAATAAATATCTCACGTAAACCTTCCTGAAAGATAAACGTTTTCCTGAATTCAGAAACTAGTAGCTAACATTGCTTCTGGAGAGCAGAAATAAGCATGTCTTCTG |
| Base | G |
| Structure | ((((((((.((........((((.((((((..((((.....(((((........)))))..))))..))))))))))((((((..((((..(((..(((.((((.....)))).)))....((((((..((((((((.................)))))....)))))))))...........))))))).)))))).))))))))))....((((.(((((((.....))))....)))))))...... |
| Colors | 34-38:green 46-50:green 87-91:green 141-145:green 163-167:green 200-204:green 211-215:green 5:orange |

Show reference structure

#### Alternate

|  |  |
| --- | --- |
| Sequence | TGCTCGTTCTCGGCATCATGATTTCCACCACATGAACTTCATTGGAAACTATGCTTCAACATTTACATGGTGGGATCGAATTTTTGGAACAGACTCTCAGTATAATGCCTATAATGAAAAGAGGAAGAAGTTTGAGAAAAAGACTGAATAAATATCTCACGTAAACCTTCCTGAAAGATAAACGTTTTCCTGAATTCAGAAACTAGTAGCTAACATTGCTTCTGGAGAGCAGAAATAAGCATGTCTTCTG |
| Base | C |
| Structure | (((((((((.(((.....(((((.((((((..((((.....(((((........)))))..))))..))))))))))).....)))))))......(((.((((.....)))).)))....((((((..((((((((.................)))))....)))))))))(((((.......)))))(((....)))...((((.(((.......))).)))).)))))................... |
| Colors | 34-38:green 46-50:green 87-91:green 141-145:green 163-167:green 200-204:green 211-215:green 5:orange |

Show alternate structure
