# Supplementary material for: Homozygous EPRS1 missense variant causing hypomyelinating leukodystrophy-15 alters variant-distal mRNA m6A site accessibility
Source: Nat Commun. 2024 May 20;15:4284. doi: 10.1038/s41467-024-48549-x (PMC11106242; doi:10.1038/s41467-024-48549-x)
Supplement: Supplementary file 4 — Supplementary Software 1 [file 41467_2024_48549_MOESM4_ESM.zip › m6Ad-SNV-prediction/output/index/data/334890_NM_001378446.1.html]

RNAPlot - 334890 - NM\_001378446.1


## Target ID: 334890\_NM\_001378446.1

https://www.ncbi.nlm.nih.gov/clinvar/variation/334890/

https://www.ncbi.nlm.nih.gov/nuccore/NM\_001378446.1

#### Reference

|  |  |
| --- | --- |
| Sequence | CCCTGGAGAGGCAGATGCAGCCTCAGGACCAGCTGGCTTCTGACTTTGGCCAGGTAGGACTTGACTTAGAGGCAGAATTTGAAAAAGATCTCAAAGACGTGCTCGGTTTTCATGAAGCCAATGACGGCGGTTTCTGGACTCTTCCTTGACCCTGTTCTGTAAAGACTGAAGCATCCCCACCTCAGGATTCAGCTGATGGGACCCTGGCTTGGACTGTTGATTGCCAGTGAGTCTGGGATGTAATTGGCTG |
| Base | G |
| Structure | ........((.(((.((((.(((..((((..((((((.((.(((...(((((((((((......))))..((((...(((((........)))))....)))).((((((...))))))....((((.((((...(((....)))..))))))))(((....)))..........((((..((((.......))))))))..)))))))......)))))..))))))..))))))).)))).))).)). |
| Colors | 26-30:green 41-45:green 57-61:green 62-66:green 136-140:green 147-151:green 163-167:green 199-203:green 211-215:green 129:orange |

Show reference structure

#### Alternate

|  |  |
| --- | --- |
| Sequence | CCCTGGAGAGGCAGATGCAGCCTCAGGACCAGCTGGCTTCTGACTTTGGCCAGGTAGGACTTGACTTAGAGGCAGAATTTGAAAAAGATCTCAAAGACGTGCTCGGTTTTCATGAAGCCAATGACGGCAGTTTCTGGACTCTTCCTTGACCCTGTTCTGTAAAGACTGAAGCATCCCCACCTCAGGATTCAGCTGATGGGACCCTGGCTTGGACTGTTGATTGCCAGTGAGTCTGGGATGTAATTGGCTG |
| Base | A |
| Structure | ........((.(((.((((.(((..((((..((((((.((.(((...(((((((((((......))))..((((...(((((........)))))....)))).((((.....((..(((..(((....)))..)))..))......))))..(((((((..((.((((...((((........)))))))))).))))))))))))))......)))))..))))))..))))))).)))).))).)). |
| Colors | 26-30:green 41-45:green 57-61:green 62-66:green 136-140:green 147-151:green 163-167:green 199-203:green 211-215:green 129:orange |

Show alternate structure
