# Supplementary material for: Homozygous EPRS1 missense variant causing hypomyelinating leukodystrophy-15 alters variant-distal mRNA m6A site accessibility
Source: Nat Commun. 2024 May 20;15:4284. doi: 10.1038/s41467-024-48549-x (PMC11106242; doi:10.1038/s41467-024-48549-x)
Supplement: Supplementary file 4 — Supplementary Software 1 [file 41467_2024_48549_MOESM4_ESM.zip › m6Ad-SNV-prediction/output/index/data/310487_NM_024685.4.html]

RNAPlot - 310487 - NM\_024685.4


## Target ID: 310487\_NM\_024685.4

https://www.ncbi.nlm.nih.gov/clinvar/variation/310487/

https://www.ncbi.nlm.nih.gov/nuccore/NM\_024685.4

#### Reference

|  |  |
| --- | --- |
| Sequence | AGACAGGTTTGGAATCAGTAATGGGTAAATACCAGCTACTAACTTCAGTTCTTCAGTGTTTGACAAAAATATTAACCATTGACATGGTAATCACTGTTAAGAGACACCCTCAGAAAGTTCACAATCAAGATTCAGAAGATGAACTATAACATCAGAAGTTTTTAATTAACCAAACTTTTCATCTAACTCAAGCCAAGTAAAGCAGTCATGTGACCACTGGTTCTAAAGTCAGTTCAGTCTACTTAGGAAA |
| Base | A |
| Structure | .((.((((((((....((((.(((.......)))..))))((((((..((((...((((((((((.....((((.((((....))))))))...)))))...)))))....))))((((((...((........))...)))))).........)))))).........)))))))).))...........((((.((...(((....)))....))))))(((.((((.((......)))))).))).. |
| Colors | 1-5:green 40-44:green 61-65:green 73-77:green 80-84:green 102-106:green 141-145:green 147-151:green 167-171:green 172-176:green 184-188:green 211-215:green 236:orange |

Show reference structure

#### Alternate

|  |  |
| --- | --- |
| Sequence | AGACAGGTTTGGAATCAGTAATGGGTAAATACCAGCTACTAACTTCAGTTCTTCAGTGTTTGACAAAAATATTAACCATTGACATGGTAATCACTGTTAAGAGACACCCTCAGAAAGTTCACAATCAAGATTCAGAAGATGAACTATAACATCAGAAGTTTTTAATTAACCAAACTTTTCATCTAACTCAAGCCAAGTAAAGCAGTCATGTGACCACTGGTTCTAAAGTCAGTTCCGTCTACTTAGGAAA |
| Base | C |
| Structure | .(((...((((((((((((..(((.......)))((((((..(((.((((....(((((((.....))))))).(((((....)))))............(((.....)))....((((((...((........))...))))))........((((((((..........)))))))).....)))).)))...)))..))).(((....))).)))))))))))))))..((((.........)))). |
| Colors | 1-5:green 40-44:green 61-65:green 73-77:green 80-84:green 102-106:green 141-145:green 147-151:green 167-171:green 172-176:green 184-188:green 211-215:green 236:orange |

Show alternate structure
