# Supplementary material for: Homozygous EPRS1 missense variant causing hypomyelinating leukodystrophy-15 alters variant-distal mRNA m6A site accessibility
Source: Nat Commun. 2024 May 20;15:4284. doi: 10.1038/s41467-024-48549-x (PMC11106242; doi:10.1038/s41467-024-48549-x)
Supplement: Supplementary file 5 — Reporting Summary [file 41467_2024_48549_MOESM5_ESM.pdf]

Corresponding author(s): Paul L. Fox
Last updated by author(s): Apr 15, 2024

Reporting Summary

Nature Portfolio wishes to improve the reproducibility of the work that we publish. This form provides structure for consistency and transparency in reporting. For further information on Nature Portfolio policies, see our Editorial Policies and the Editorial Policy Checklist.

Statistics

For all statistical analyses, confirm that the following items are present in the figure legend, table legend, main text, or Methods section.

n/a	Confirmed	
☐	☒	The exact sample size (*n*) for each experimental group/condition, given as a discrete number and unit of measurement
☐	☒	A statement on whether measurements were taken from distinct samples or whether the same sample was measured repeatedly
☐	☒	The statistical test(s) used AND whether they are one- or two-sided<br>*Only common tests should be described solely by name; describe more complex techniques in the Methods section.*
☒	☐	A description of all covariates tested
☒	☐	A description of any assumptions or corrections, such as tests of normality and adjustment for multiple comparisons
☐	☒	A full description of the statistical parameters including central tendency (e.g. means) or other basic estimates (e.g. regression coefficient) AND variation (e.g. standard deviation) or associated estimates of uncertainty (e.g. confidence intervals)
☐	☒	For null hypothesis testing, the test statistic (e.g. *F*, *t*, *r*) with confidence intervals, effect sizes, degrees of freedom and *P* value noted<br>*Give P values as exact values whenever suitable.*
☒	☐	For Bayesian analysis, information on the choice of priors and Markov chain Monte Carlo settings
☒	☐	For hierarchical and complex designs, identification of the appropriate level for tests and full reporting of outcomes
☒	☐	Estimates of effect sizes (e.g. Cohen's *d*, Pearson's *r*), indicating how they were calculated

Our web collection on statistics for biologists contains articles on many of the points above.

Software and code

Policy information about availability of computer code

Data collection	Spectramax i3X system and SoftMax Pro v6.5.1 (Molecular Devices)<br>Wallac Victor3 1420 multilabel counter system and Wallac 1420 Manager v3 (Perkin Elmer)<br>Step One Plus Real Time PCR sytem and StepOne v2.3 (Applied Biosystems)<br>NGC Chromatography System with ChromLab v5.0.2.11 (BioRad)
Data analysis	Graphpad Prism 9 and 10 (https://www.graphpad.com/scientific-software/prism/)<br>ImageJ v1.51k (https://imagej.nih.gov/ij/)<br>Fiji v2.0.0-rc-69/1.52n (https://fiji.sc/)<br>MFOLD RNA Folding Form v2.3 (http://www.unafold.org/mfold/applications/rna-folding-form.php)<br>RNAstructure v6.4 (https://rna.urmc.rochester.edu/RNAstructure.html)<br>RNAFold ViennaRNA Package 2.0 (https://rna.tbi.univie.ac.at/cgi-bin/RNAWebSuite/RNAfold.cgi)<br>Clustal Omega v1.2.4 (https://www.ebi.ac.uk/Tools/msa/clustalo/)<br>WebLogo3 v2.8.2 (https://weblogo.berkeley.edu/logo.cgi)<br>SWISS-MODEL webserver with ProMod3 modelling engine v3.4.0 (https://swissmodel.expasy.org/interactive)<br>GROMACS v2023.1 (https://www.gromacs.org)<br>Pymol v2.5.0 (https://pymol.org/2/)<br>PANTHER 17.0 (https://www.pantherdb.org/)<br>DAVID Bioinformatics Resource- DAVID 2021 with DAVID Knowlegebase v2023q4 (https://david.ncifcrf.gov/summary.jsp)

The data analysis pipeline for m6Ad-SNV prediction is described in Methods. Reiterated below:
We extracted a BED file from the UCSC Genome Browser (https://genome.ucsc.edu/) containing the genomic coordinates of whole gene regions with transcripts from NCBI RefSeq for the Human Genome version GRCh38. We retrieved the Human Genome version GRCh38 fasta file also from the Genome Browser at https://hgdownload.soe.ucsc.edu/goldenPath/hg38/bigZips/hg38.fa.gz. We focused on protein-coding regions only by looking at the GENCODE (release 43) annotations available at https://ftp.ebi.ac.uk/pub/databases/gencode/Gencode_human/release_43/gencode.v43.annotation.gtf.gz. We also extracted the whole set of variations from the ClinVar database as a VCF file (version 20230619 available at https://ftp.ncbi.nlm.nih.gov/pub/clinvar/vcf_GRCh38/weekly/clinvar_20230617.vcf.gz) together with the curated set of m6A modifications from the RMVar database (https://rmvar.renlab.org/download.html) to focus on m6A variations only. Our pipeline for the analysis of variant-dependent m6A modifications within the human genome has been implemented from scratch in Python. It makes use of the following Python packages: biopython v1.78, pysam v0.16.0.1, pandas v1.3.5, wget v3.2, tqdm v4.42.1.It also requires RNAFold as part of the ViennaRNA package v2.6.4 (https://github.com/ViennaRNA/ViennaRNA/releases/tag/v2.6.4). The source code is hosted on GitHub in a non-public repository that can be inspected upon request. As per your guidelines, we attach a zip archive with the software, inputs, license, and a description about how to use the software. Results produced by the execution of our software are available at the same non-public GitHub repository where the software is hosted, and can be inspected upon request. Results are also available in the attached zip archive with the python pipeline, input data, and a description about how to run the software.

The zip archive 'm6Ad-SNV-prediction.zip' contains:
1. a README.txt file containing a short description of the content of the archive and a brief instruction about how to run the pipeline;
an "input" folder with the BED file retrieved from the UCSC Genome Browser with whole gene regions;
2. an "output" folder with the results of the pipeline;
3. a "src" folder with the actual software pipeline.

Please note that:

A. the "input" folder contains just the BED file, but the pipeline automatically retrieves (i) the GENCODE annotation to extract the protein-coding regions, (ii) the ClinVar VCF file, (iii) the RMVar information about the m6A modifications, and (iv) the human genome version GRCh38. All these files will be automatically downloaded in the "input" folder;

B. the "src" folder contains a "pipeline.sh" file for an automatic execution of the pipeline, from the retrieval of the files listed above to the actual algorithm described in the manuscript. It may be easier for a reader to just execute this single file. IThe README.txt explains what the "pipeline.sh" does in particular, including a list of software requirements that a reader must install for its execution.

For manuscripts utilizing custom algorithms or software that are central to the research but not yet described in published literature, software must be made available to editors and reviewers. We strongly encourage code deposition in a community repository (e.g. GitHub). See the Nature Portfolio guidelines for submitting code & software for further information.

Data

Policy information about availability of data
All manuscripts must include a data availability statement. This statement should provide the following information, where applicable:
- Accession codes, unique identifiers, or web links for publicly available datasets
- A description of any restrictions on data availability
- For clinical datasets or third party data, please ensure that the statement adheres to our policy

DATA AVAILABILITY STATEMENT:
Source data are provided with this paper. All graph data used in this study are available in the accompanying Source Data file. All raw micrographs used in Figures and Supplementary Figures are available in the accompanying Source Data file. All oligonucleotide sequences (PMOs, primers, SELECT-qPCR probes) and plasmid-based CRISPR gRNA spacer sequences are detailed in Supplementary Table 4 in Supplementary Information file. EPRS1 variant studied is ClinVar ID xxx [link:xxx]. EPRS1 gene IDs used for alignment in Supplementary Fig. 10b are as follows: Homo sapiens [https://www.ncbi.nlm.nih.gov/gene/2058], Mus musculus [https://www.ncbi.nlm.nih.gov/gene/107508], Rattus norvegicus [https://www.ncbi.nlm.nih.gov/gene/289352], Otolemur garnettii [https://www.ncbi.nlm.nih.gov/gene/100966256], Lemur catta [https://www.ncbi.nlm.nih.gov/gene/123627005], Microcebus murinus [https://www.ncbi.nlm.nih.gov/gene/103257617], Carlito syrichta [https://www.ncbi.nlm.nih.gov/gene/105886059], Sapajus apella [https://www.ncbi.nlm.nih.gov/gene/116558954], Macaca mulatta [https://www.ncbi.nlm.nih.gov/gene/706899], Pan troglodytes [https://www.ncbi.nlm.nih.gov/gene/457746], Gorilla gorilla gorilla [https://www.ncbi.nlm.nih.gov/gene/101132255], Eptesicus fuscus [https://www.ncbi.nlm.nih.gov/gene/103293465], Tupaia chinensis [https://www.ncbi.nlm.nih.gov/gene/102472024], Galeopterus variegatus [https://www.ncbi.nlm.nih.gov/gene/103603328], Castor canadensis [https://www.ncbi.nlm.nih.gov/gene/109682484], Sciurus carolinensis [https://www.ncbi.nlm.nih.gov/gene/124962055], Oryctolagus cuniculus [https://www.ncbi.nlm.nih.gov/gene/100340173], Canis lupus familiaris [https://www.ncbi.nlm.nih.gov/gene/478962], Delphinapterus leucas [https://www.ncbi.nlm.nih.gov/gene/111177482], Tursiops truncates [https://www.ncbi.nlm.nih.gov/gene/101332069], Bos taurus [https://www.ncbi.nlm.nih.gov/gene/538357], Ovis aries [https://www.ncbi.nlm.nih.gov/gene/101104590], Desmodus rotundus [https://www.ncbi.nlm.nih.gov/gene/112318748], Crotalus tigris [https://www.ncbi.nlm.nih.gov/gene/120307188], Notechis scutatus [https://www.ncbi.nlm.nih.gov/gene/113421898], Thamnophis elegans [https://www.ncbi.nlm.nih.gov/gene/116507382], Thamnophis sirtalis [https://www.ncbi.nlm.nih.gov/gene/106547182], Alligator sinensis [https://www.ncbi.nlm.nih.gov/gene/102381974], Alligator mississippiensis [https://www.ncbi.nlm.nih.gov/gene/102565153], Mauremys mutica [https://www.ncbi.nlm.nih.gov/gene/120400723]. Rabbit beta globin intron 2 sequence was from HBB2 [https://www.ncbi.nlm.nih.gov/gene/100009084] and chimeric intron was from pCI vector [https://www.ncbi.nlm.nih.gov/nuccore/U47119.2]. EPRS1 PDB structures used for MDS are 4HVC [https://pdbj.org/mine/summary/4hvc] and 5VAD [https://pdbj.org/mine/summary/5vad]. The m6A RNA methylation data used in m6Ad-SNVs prediction is available in RMVar database [https://rmvar.renlab.org/] and used as detailed in Methods. The ClinVar variants data used in m6Ad-SNVs prediction is available in ClinVar database [https://www.ncbi.nlm.nih.gov/clinvar/] and used as detailed in Methods. Human reference sequence transcripts used in m6Ad-SNVs prediction are available in [https://www.ncbi.nlm.nih.gov/refseq/] and used as detailed in Methods. Detailed superset of Supplementary Table 3 is available at [https://doi.org/10.5281/zenodo.10905850] (interactive and machine-readable results produced by the m6Ad-SNVs prediction tool, contains transcript IDs, gene symbols, ClinVar ID, predicted RNA structures and available DRACH sites). Any additional information, if needed, is available from the lead contact Paul Fox (foxp@ccf.org).

CODE AVAILABILITY STATEMENT:
The code is available at [https://doi.org/10.5281/zenodo.10905411] linked to GitHub at [https://github.com/cumbof/m6Ad-SNVs] under an MIT license.

Research involving human participants, their data, or biological material

Policy information about studies with human participants or human data. See also policy information about sex, gender (identity/presentation), and sexual orientation and race, ethnicity and racism.

Reporting on sex and gender	This is a case-level report where gender and sex were not significant determinants of outcome. We describe an autosomal recessive condition in two siblings, one male and one female. No sex or gender-based analyses were performed based on a sample size of one male and one female participant. Disaggregated data for sex is shown by symbols in all relevant figures and have been tabulated separately in the Source Data file.
Reporting on race, ethnicity, or other socially relevant groupings	No socially relevant groupings were carried out in this report of a single family.
Population characteristics	This is a report of a single family consisting of two siblings; an 18 year old male and 16 year old female with severe cognitive and motor impairment and progressive hypomyelination due to the same homozygous missense variant c.4444C>A; p.Pro1482Thr in EPRS1. As this variant is unique to this family, we are not able to stratify our analyses based on population-based co-variates (such as environmental factors) and have focused our analyses on the biological consequences of the variant.
Recruitment	Both participants were followed in a specialized Neurogenetics Clinic and written informed consent was obtained to participate in this research study, with the goal of clarifying the results of clinical genetic testing. The EPRS1 variant is classified as being of uncertain significance by the clinical laboratory, and further studies are required to provide definitive evidence of pathogenicity. There are no elements of the recruitment process which would impact the results of the functional studies of the biology of the variant and its role in causing the clinical symptoms observed in the participants.
Ethics oversight	This study was approved by the Research Ethics Board of the Hospital for Sick Children

Note that full information on the approval of the study protocol must also be provided in the manuscript.

Field-specific reporting

Please select the one below that is the best fit for your research. If you are not sure, read the appropriate sections before making your selection.

☒ Life sciences ☐ Behavioural & social sciences ☐ Ecological, evolutionary & environmental sciences

For a reference copy of the document with all sections, see nature.com/documents/nr-reporting-summary-flat.pdf

Life sciences study design

All studies must disclose on these points even when the disclosure is negative.

Sample size	Appropriate sample sizes were determined by a heuristic approach, based on previous experience in protein expression and post-transcriptional gene regulation (e.g. PMID: 12588972, PMID: 17611605; PMID: 19098893 and PMID: 37296097), and experimental results showing statistical significance.
Data exclusions	Data points were not excluded, except in a quantitation of YTHDC1 in Supplementary Figure 7b necessitated by a variation in immunoblot results in 1 of 4 replicates that was deemed as a technical error in film exposure based on combined experience of authors, where outlier correction was done by Grubbs' test as detailed in the Methods and in the Figure Legend.
Replication	For quantitative assays, biological replicates were employed and results were reliably replicated across at least two independent biological replicates over at least two independent experiments. No quantitative experiment reported is solely from technical replicates. All non-quantitative results were replicated at least twice, except Supplementary Figure 13a that was done once (an in-gel protein staining to determine FTO enzyme integrity and concentration for downstream assay, where a limited amount of enzyme was sourced commercially).
Randomization	Randomization was not necessary as we employed a controlled before-and-after quasi-experimental design, where one group is exposed to a change (genetic/environmental), and measured against a control group not exposed to that change. Groups are similar on all estimable known and unknown factors except for exposure to the change.
Blinding	Blinding during collection was not needed because conditions were in vitro and well-controlled. Blinding is also not necessary because the results are quantitative and did not require subjective judgment or interpretation.

Reporting for specific materials, systems and methods

We require information from authors about some types of materials, experimental systems and methods used in many studies. Here, indicate whether each material, system or method listed is relevant to your study. If you are not sure if a list item applies to your research, read the appropriate section before selecting a response.

Materials & experimental systems

n/a	Involved in the study
☐	☒ Antibodies
☐	☒ Eukaryotic cell lines
☒	☐ Palaeontology and archaeology
☒	☐ Animals and other organisms
☒	☐ Clinical data
☒	☐ Dual use research of concern
☒	☐ Plants

Methods

n/a	Involved in the study
☒	☐ ChIP-seq
☒	☐ Flow cytometry
☐	☒ MRI-based neuroimaging

Antibodies

Antibodies used	Refer to Supplementary Table 4 for antibody RRID, clone number [monoclonals], dilutions and vendors.
Validation	Refer to Supplementary Table 4 for antibody validation details, vendor statements and references. All antibodies used are validated in some form.

Eukaryotic cell lines

Policy information about cell lines and Sex and Gender in Research

Cell line source(s)	Refer to Methods and Supplementary Table 4 for details. All cell lines used from LRI Cell Culture Core are sourced from ATCC.<br>Human: 293 F (HEK293F). Possibly female.<br>Human: HEK293T. Possibly female.<br>Human: U87-MG. Male.<br>Control LCL 1: De-identified, from Genome Medicine Biorepository, Genome Medicine Institute, Cleveland Clinic LRI. Adult male, Indian ancestry.<br>Control LCL 2 De-identified, from Genome Medicine Biorepository, Genome Medicine Institute, Cleveland Clinic LRI. Adult male, Indian ancestry.<br>Control LCL 3: De-identified, from Hospital for Sick Children's, Toronto. Adult female, East Indian descent.<br>Parent LCLs: Generated in this study. One male, one female.<br>Sibling LCLs: Generated in this study. One male, one female.
Authentication	None of the cell lines used were authenticated as these were either generated in-house or sourced primarily from ATCC or Thermo Fisher.
Mycoplasma contamination	Cell lines tested negative for mycoplasma. Tested monthly with Lonza Mycoalert Plus detection kit (Catalog #: LT07-710).
Commonly misidentified lines<br>(See ICLAC register)	It has been pointed out that ATCC stocks of U87-MG come from a different donor to the Uppsala stocks (PMID: 27582061 and Version 12 Table 2, misidentified cell lines, https://iclac.org/databases/cross-contaminations/). The ATCC stocks have CNS origin and is likely a bona fide human glioblastoma cell line, similar to Uppsala stocks, allowing its limited use in a role that is only secondary to patient-derived LCLs and HEK293T in this study. Since sourced from ATCC, these cells were not authenticated further.<br>This information has now been included in Methods Section.

Plants

Seed stocks	N/A
Novel plant genotypes	N/A
Authentication	N/A

Magnetic resonance imaging

Experimental design

Design type	case report - description of structural anomalies of two patients

Design specifications | N/A

Behavioral performance measures | N/A

Acquisition

Imaging type(s) | structural

Field strength | 1.5 Tesla

Sequence & imaging parameters | T1: TR 9.9, TE 4.6, ST 1mm, matrix 200x200, FOV 20 cm; multiplanar reformatted images performed
T2: TR 2475, TE 120, ST 5 mm, matrix 400x304, FOV 20 cm
FLAIR: TR 5000, TE 140, ST 5 mm, matrix 268x234, FOV 20 cm

Area of acquisition | whole brain

Diffusion MRI ☐ Used ☒ Not used

Preprocessing

Preprocessing software | N/A

Normalization | N/A

Normalization template | N/A

Noise and artifact removal | N/A

Volume censoring | N/A

Statistical modeling & inference

Model type and settings | N/A

Effect(s) tested | N/A

Specify type of analysis: ☐ Whole brain ☐ ROI-based ☐ Both

Statistic type for inference | N/A

(See Eklund et al. 2016)

Correction | N/A

Models & analysis

n/a	Involved in the study
☒	☐ Functional and/or effective connectivity
☒	☐ Graph analysis
☒	☐ Multivariate modeling or predictive analysis
